# Photoinduced Reductive C–C and C–Heteroatom Couplings from Bis-cyclometalated Pt(IV) Alkynyl Complexes

**DOI:** 10.1021/acs.inorgchem.3c02162

**Published:** 2023-08-24

**Authors:** Juan Carlos López-López, Delia Bautista, Pablo González-Herrero

**Affiliations:** † Departamento de Química Inorgánica, Facultad de Química, Universidad de Murcia, Campus de Espinardo, 19, 30100 Murcia, Spain; ‡ Área Científica y Técnica de Investigación, Universidad de Murcia, Campus de Espinardo, 21, 30100 Murcia, Spain

## Abstract

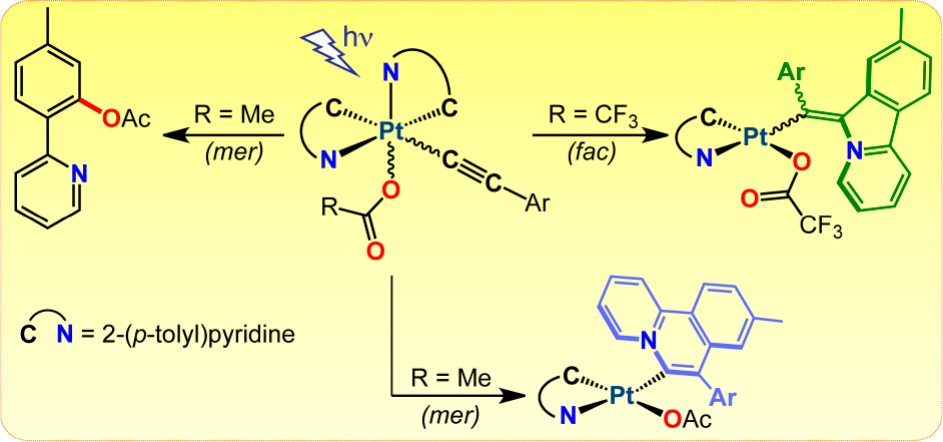

Unsymmetrical dicarboxylato complexes [Pt(tpy)_2_(O_2_CR)_2_] [tpy = cyclometalated 2-(*p*-tolyl)pyridine, R = Me, CF_3_] react with the terminal alkynes 4-methoxyphenylacetylene, phenylacetylene, 4-(trifluoromethyl)phenylacetylene or 3,5-difluorophenylacetylene in the presence of a base to produce complexes *mer*-[Pt(tpy)_2_(O_2_CR)(CCAr)], in which the metalated carbon atoms are in a meridional arrangement. Irradiation of the trifluoroacetato derivatives with a 365 nm LED source leads to isomerization to the facial complexes, which can be converted to chlorido derivatives upon reaction with NH_4_Cl. In contrast, irradiation of the acetato derivatives leads to four different processes, namely, reduction to *cis*-[Pt(tpy)_2_], annulations involving one of the tpy ligands and the C_α_ and C_β_ atoms of the alkynyl to give benzoquinolizinium derivatives, isomerization to the facial geometry, or C–O couplings between the acetato ligand and one tpy. The first two processes are favored by the presence of electron-donating groups on the alkynyl, whereas electron-withdrawing groups favor the last two. Irradiation of complexes *fac*-[Pt(tpy)_2_(O_2_CCF_3_)(CCAr)] with a medium-pressure Hg UV lamp leads to a reductive C–C coupling involving the alkynyl C_α_ atom and one of the tpy ligands to give pyridoisoindolium derivatives, except for the methoxyphenylacetylide derivative, which is photostable. On the basis of TDDFT calculations, the photoreactivity of the *mer* complexes is attributed to ^3^LLCT [π(alkynyl) → π*(tpy)] excited states for annulations or ^3^LMCT [π(alkynyl) → dσ*] excited states for the rest of the processes, which are accessible through thermal population from ^3^LC(tpy) states. The C–C couplings from the *fac* complexes are attributed to photoreactive pentacoordinate intermediates.

## Abstract 2

Different types of photoinduced reductive C−C and C−heteroatom couplings are demonstrated to occur from bis-cyclometalated alkynyl(carboxylato) Pt(IV) complexes, depending on the electronic properties and arrangement of the ligands. The possible reactive excited states are examined through TDDFT.

## Introduction

There is a growing interest in development of photochemical processes mediated by transition-metal complexes with applications in synthesis and catalysis.^1−3^ The formation of C–C and C–heteroatom bonds via reductive elimination are crucial steps,^4^ and considerable efforts are currently being devoted to implement photochemical strategies that promote them under mild conditions. Thus, diverse reductive couplings have been recently discovered to occur from transition metal complexes in the excited state, most often with nickel and palladium, and have been incorporated into useful catalytic cycles.^5−10^ In this context, the photochemistry of coordination and organometallic complexes has gained renewed interest.

The photoreactivity of Pt(IV) complexes has been investigated mostly in connection with the development of photoactivatable prodrugs for the treatment of cancer.^11−19^ Typically, Pt(IV) coordination complexes exhibit low-lying ligand-to-metal charge-transfer (LMCT) excited states, whose population via photoexcitation promotes ligand dissociation and reduction of the metal because they involve electronic transitions to strongly antibonding dσ* orbitals.^20^ Photoreductive eliminations from Pt(IV) complexes have also been the subject of significant studies dealing with the halogenation of aromatic compounds,^21,22^ the elimination of halogen atoms associated with the development of photoinduced hydrogen halide splitting processes,^23^ and the photogeneration of hydroxyl radicals.^24,25^

**Chart 1:**
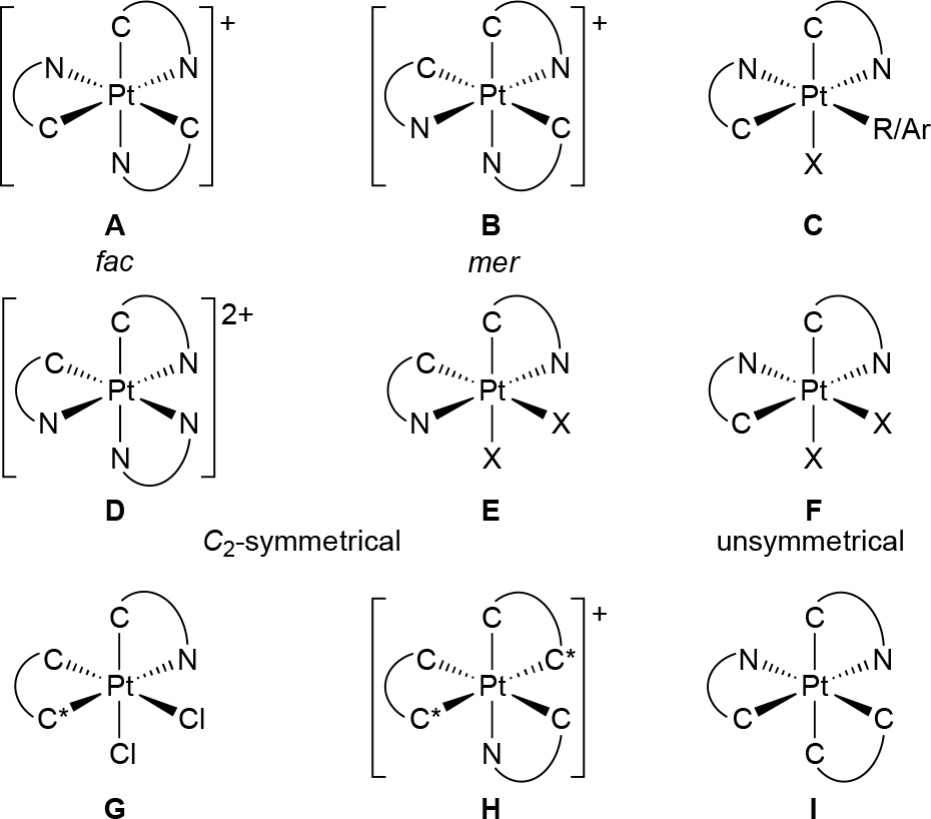
Reported Classes of Pt(IV) Complexes with Cyclometalated 2-Arylpyridinesa

Previous works from ours and other laboratories have been devoted to the study of the photophysics of Pt(IV) complexes with cyclometalated 2-arylpyridines (C^∧^N) and related ligands.^26−41^ Most of the reported examples can be classified into the structural types shown in Chart 1. They may exhibit long-lived phosphorescent emissions arising from triplet ligand-centered (^3^LC or ^3^π–π*) excited states mainly localized on the C^∧^N ligands, with a very small metal-to-ligand charge-transfer (MLCT) contribution. Very high emission quantum yields can be attained from structural types **A** and **C**, featuring a facial (*fac*) arrangement of C-donor moieties that induce a strong ligand field.^26,29,30,34−37^ Similarly, types **G**, **H**, and **I** display strong emissions thanks to the presence strongly σ-donating cyclometalated aryl-*N*-heterocyclic carbenes^40,41^ or dimetalated biaryls^31^ as ancillary, nonchromophoric ligands. In contrast, the majority of *C*_2_-symmetrical bis-cyclometalated Pt(IV) complexes that lack strong-field ancillary ligands (**D**, **E**) are weak emitters because the lower energies of their dσ* orbitals result in the presence of thermally accessible ^3^LMCT states [π*(C^∧^N) → dσ*] that provide a pathway for nonradiative deactivation.^27,32,36^ Additionally, the presence of anionic ancillary ligands (X) with available lone pairs on the donor atom, such as heavier halides, originate ^3^LMCT [p(X) → dσ*] and ligand-to-ligand charge-transfer [^3^LLCT; p(X) → π*(C^∧^N)] states that have also a detrimental effect on luminescence.^36^ Tris-cyclometalated complexes with a meridional (*mer*) geometry (**B**)^34,35^ and unsymmetrical bis-cyclometalated complexes (**F**)^36,42^ may present photochemical reactivity and some of them undergo isomerization reactions under irradiation with UV light to give the *fac* or *C*_2_-symmetrical isomers, respectively, because of the population of dissociative ^3^LMCT states.

a C^∧^N = Cyclometalated 2-arylpyridine. C^∧^C* = Cyclometalated aryl-NHC. C^∧^C = dimetalated biaryl. X = Cl^–^, Br^–^, I^–^, RCOO^–^. R/Ar = Alkyl or aryl.

Based on these precedents, we envisioned that the combination of an unsymmetrical arrangement of C^∧^N ligands, which usually leads to low energies of dσ* orbitals, with the introduction of an alkynyl ligand, having π orbitals at relatively high energies from which charge-transfer transitions could take place, would lead to photochemical reactivity. In this paper, we report the synthesis and photophysical characterization of bis-cyclometalated alkynyl(carboxylato) Pt(IV) complexes and show that they can perform different types of light-induced reactions, including reductive C–C and C–heteroatom couplings. We also provide an assignment of the reactive excited states based on time-dependent density functional theory (TDDFT) calculations and the influence of the electronic properties of the alkynyl and carboxylato ligands on their photoreactivity.

## Results and Discussion

### Synthesis and Characterization of Bis-cyclometalated Pt(IV) Alkynyl Complexes

**Scheme 1:**
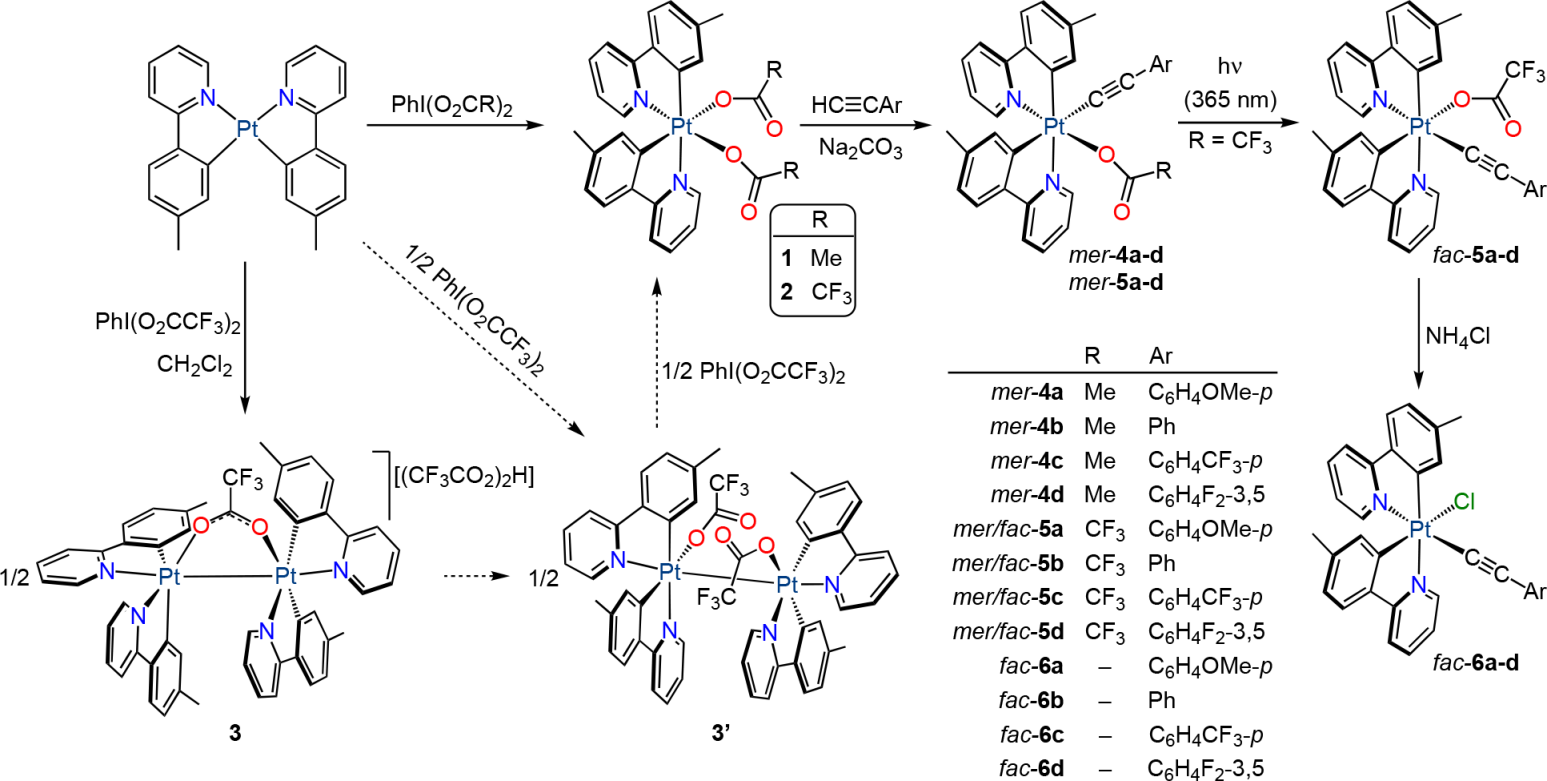
Synthesis of Bis-cyclometalated Pt(IV) Alkynyl Complexes

We initially sought to obtain unsymmetrical dicarboxylato complexes [Pt(tpy)_2_(O_2_CR)_2_], where tpy = cyclometalated 2-(*p*-tolyl)pyridine and R = Me (**1**), CF_3_ (**2**) (Scheme 1), which we hypothesized as suitable precursors for the synthesis of Pt(IV) alkynyls through the substitution of carboxylato ligands. The preparation of the analogous dicarboxylato complexes bearing cyclometalated 2-phenylpyridine (ppy) was described by Whitfield and Sanford,^43,44^ who employed the oxidation of *cis*-[Pt(ppy)_2_] with the appropriate hypervalent iodine reagent PhI(O_2_CR)_2_ in CH_2_Cl_2_; however, the bis(trifluoroacetato) complex was obtained together with the Pt(III) complex [{Pt(ppy)_2_(O_2_CCF_3_-κ*O*)}_2_] and could only be isolated from the mixture by manual separation of the crystals. Based on this precedent, we explored the reactions of *cis*-[Pt(tpy)_2_] with PhI(O_2_CR)_2_ (R = Me, CF_3_) in a 1:1 molar ratio. Bis(acetato) complex **1** could be obtained in pure form by performing oxidation in CH_2_Cl_2_ at room temperature, requiring a minimum of 16 h to get a good yield (83%). The oxidation of *cis*-[Pt(tpy)_2_]^45^ with PhI(O_2_CCF_3_)_2_ in CH_2_Cl_2_ was much faster, but it produced a mixture of the desired bis(trifluoroacetato) complex, **2**, and the Pt(III) complex [{Pt(tpy)_2_}_2_(μ-O_2_CCF_3_-κ*O*:κ*O*′)][(CF_3_CO_2_)_2_H] (**3**) in ca. 1:1 ratio. Although this behavior seems to mimic that of *cis*-[Pt(ppy)_2_], the obtained Pt(III) dimer is different because it is cationic and contains a bridging CF_3_CO_2_^–^ ligand instead of one monodentate ligand per metal. In addition, the presence of the [(CF_3_CO_2_)_2_H]^−^ counterion suggests that part of the liberated CF_3_CO_2_· radicals abstract a hydrogen atom from the solvent instead of further oxidizing the Pt(III) centers. Complex **3** could be obtained in pure form by performing the reaction between *cis*-[Pt(tpy)_2_] and PhI(O_2_CCF_3_)_2_ (1:1) in CH_2_Cl_2_ at −90 °C, but upon recrystallization from CH_2_Cl_2_/Et_2_O at room temperature, it partially transformed into the isomeric compound [{Pt(tpy)_2_(O_2_CCF_3_-κ*O*)}_2_] (**3′**), which is analogous to the Pt(III) complex observed by Whitfield and Sanford. In addition, we verified that the addition of PhI(O_2_CCF_3_)_2_ to mixtures of **3** and **3′** resulted in oxidation of **3′** to produce **2**, whereas **3** remained unreacted, which is consistent with the outcome of the reaction between *cis*-[Pt(tpy)_2_] and PhI(O_2_CCF_3_)_2_ in CH_2_Cl_2_ at room temperature and indicates that **3′** is an intermediate in the formation of **2**. Gratifyingly, complex **2** could be obtained in good yield (87%) by adding a solution of PhI(O_2_CCF_3_)_2_ in Et_2_O to a suspension of *cis*-[Pt(tpy)_2_] in the same solvent.

The introduction of alkynyl ligands was attempted by reacting **1** and **2** with an excess of the terminal alkynes 4-methoxyphenylacetylene, phenylacetylene, 4-(trifluoromethyl)phenylacetylene, or 3,5-difluorophenylacetylene and a base (Na_2_CO_3_) in CH_2_Cl_2_. To maximize the yields, complexes **1** and **2** were prepared and used in situ. These reactions afforded derivatives of the type *mer*-[Pt(tpy)_2_(O_2_CR)(CCAr)] (*mer*-**4a**–**d**, *mer*-**5a**–**d**; Scheme 1), which were isolated in moderate to good yields with respect to *cis*-[Pt(tpy)_2_] (46–89%). These products result from the selective substitution of the carboxylato ligand trans to the metalated aryl of one of the tpy ligands for an alkynyl and therefore present a meridional arrangement of metalated carbon atoms. Such selectivity indicates that the substitution proceeds through a dissociative mechanism facilitated by the strong trans effect of the aryl group. The trifluoroacetato complexes *mer*-**5a**–**d** underwent photoisomerization to the respective facial complexes *fac*-**5a**–**d** upon irradiation with UV light (LED source, λ_max_ = 365 nm) in MeCN at room temperature with little decomposition. Substitution of the trifluoroacetato ligand in these isomers by a chloride was carried out through treatment with NH_4_Cl in acetone, affording the corresponding complexes *fac*-[Pt(tpy)_2_Cl(CCAr)] (*fac*-**6a**–**d**). The structure of these derivatives is similar to that of previously reported highly luminescent Pt(IV) complexes of the type [Pt(C^∧^N)_2_Cl(R)], with R = alkyl.^26,37^

**Figure 1:**
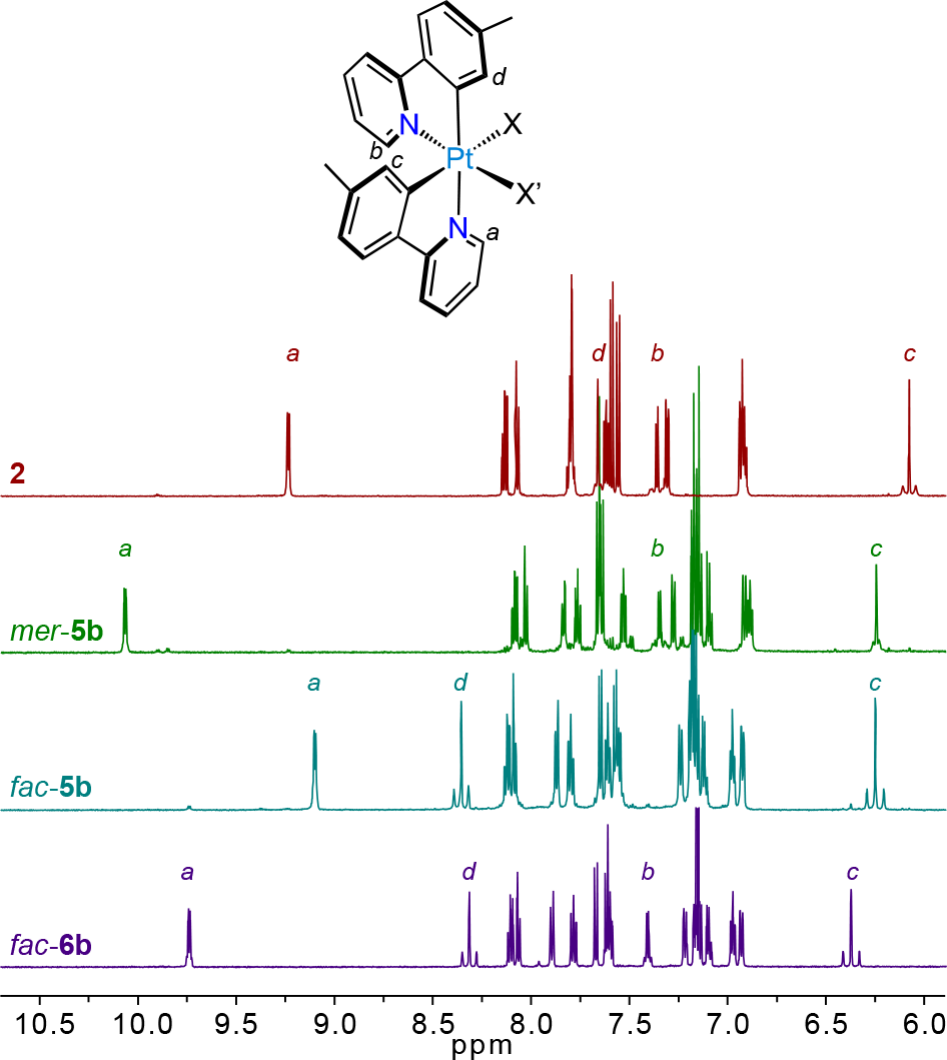
^1^H NMR spectra of complexes **2**, *mer*-**5b**, *fac*-**5b**, and *fac*-**6b** (aromatic region).

The ^1^H NMR spectra of **1**, **2**, *mer*-**4a**–**d**, *mer*/*fac*-**5a**–**d**, and *fac*-**6a**–**d** show two sets of resonances corresponding to unsymmetrically disposed tpy ligands. The resonances arising from the aromatic protons ortho to the metalated C atoms or coordinated N atoms are particularly informative on ligand arrangement because they present satellites or a broadening at the base due to the coupling with the ^195^Pt isotope and can be affected by the magnetic anisotropy of other ligands, as observed for previously reported bis-cyclometalated Pt(IV) complexes.^30,36,37,46^ To illustrate this, the ^1^H NMR spectra of **2**, *mer*-**5b**, *fac*-**5b**, and *fac*-**6b** are compared in Figure 1. The protons *b* and *c* are significantly shielded relative to the homologous *a* and *d*, respectively, because they are affected by the diamagnetic current of orthogonal aromatic rings. Upon introduction of the phenylacetylide ligand, the resonance of *a* in *mer*-**5b** is significantly shifted downfield because this proton is directed toward the deshielding region generated by the triple bond, whereas in *fac*-**5b** and *fac*-**6b**, the proton affected by the triple bond is *d*. Proton *a* in *fac*-**6b** is shifted downfield with respect to *fac*-**5b** because it is directed toward the Pt–Cl bond, which contributes to deshielding to a greater extent compared to the Pt–O bond in *fac*-**5b**; a similar effect has been observed in other bis-cyclometalated Pt(IV) complexes.^30,36^

Complex **2** gives two resonances from the inequivalent trifluoroacetato ligands in the ^19^F NMR spectrum, one of which presents ^195^Pt satellites (*J*_PtF_ = 11.3 Hz). Reasonably, this corresponds to the trifluoroacetato trans to the coordinated pyridyl ring of one of the tpy ligands because the Pt–O bond is shorter in comparison with the one trans to the metalated aryl (see below). Consistently, ^195^Pt satellites are also observed for the ^19^F resonances of *mer*-**5a**–**d**, but not for *fac*-**5a**–**d**.

**Figure 2:**
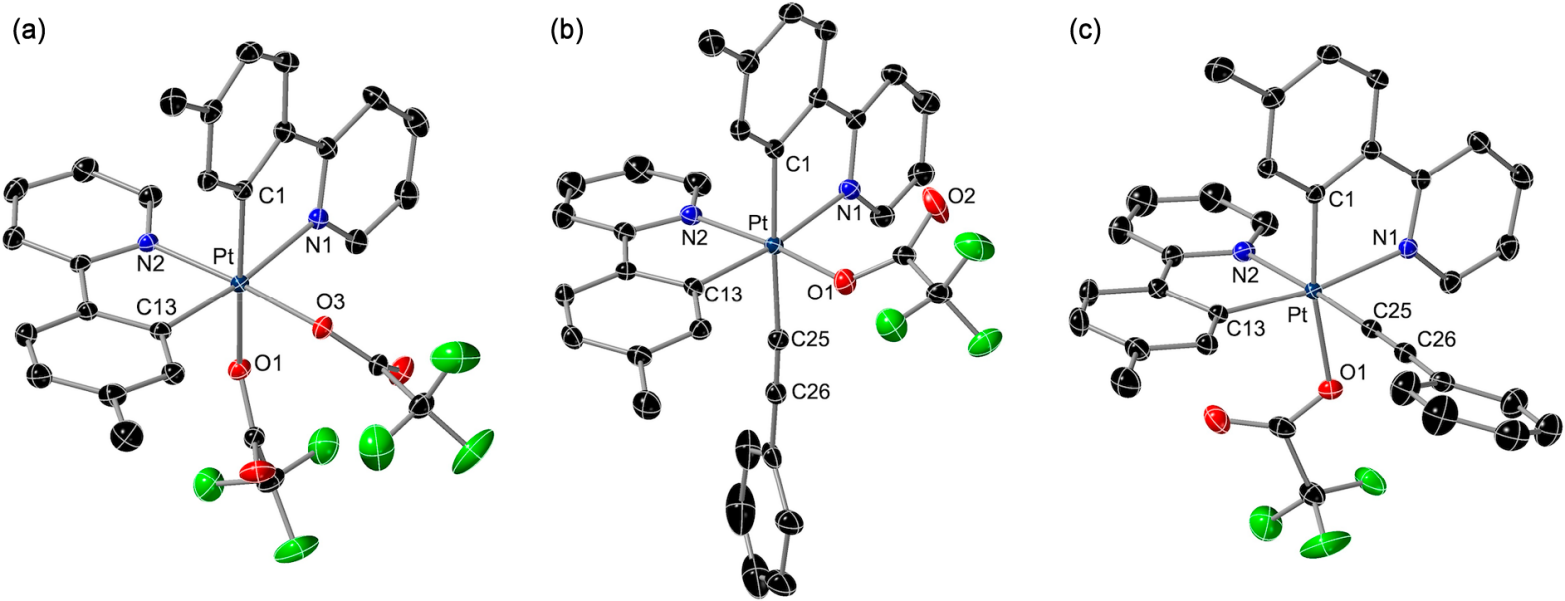
Structures of complexes **2** (a), *mer*-**5b** (b), and *fac*-**5b** (c) in the crystal (thermal ellipsoids at 50% probability). Hydrogen atoms and solvent molecules are omitted.

The crystal structures of **2**·CH_2_Cl_2_, **3**·CH_2_Cl_2_, **3′**, *mer*-**4b**, *mer*-**5b**, and *fac*-**5b**·CH_2_Cl_2_ were determined by X-ray diffraction studies. The molecular structures of **2**, *mer*-**5b**, and *fac*-**5b** are shown in Figure 2, and selected bond distances and angles are given in Table 1. The structures of **3**, **3′**, and *mer*-**4b** are presented in the Supporting Information. As expected, all of them present unsymmetrical {Pt(tpy)_2_} subunits, with mutually cis metalated aryls or coordinated pyridyl rings. In complex **2**, the Pt–O1 bond distance is ca. 0.1 Å longer than that of Pt–O3 because of the stronger trans influence of the metalated aryl with respect to the pyridyl moiety. For the same reason, the Pt–O1 distance in *fac*-**5b** is also ca. 0.1 Å longer than that in *mer*-**5b**. The strong trans influence of the alkynyl ligand is reflected in the appreciable elongation of the Pt–C1 bond in *mer*-**5b** (ca. 0.05 Å) or the Pt–N2 bond in *fac*-**5b** (ca. 0.08 Å) with respect to complex **2**.

**Table 1:** Selected Bond Distances (Å) and Angles (deg) for **2**, *mer*-**5b**, and *fac*-**5b**

	**2**	*mer*-**5b**	*fac*-**5b**
Pt–C1	2.002(2)	2.055(3)	2.0010(17)
Pt–C13	2.005(2)	2.005(3)	2.0187(17)
Pt–C25		2.064(3)	1.9651(18)
Pt–N1	2.1452(18)	2.137(2)	2.1312(14)
Pt–N2	2.0123(18)	2.019(2)	2.0876(15)
Pt–O1	2.1377(15)	2.0469(19)	2.1616(13)
Pt–O3	2.0384(15)		
O1–Pt–O3	96.79(6)		
O1–Pt–C25		92.44(10)	89.83(6)
C1–Pt–N1	80.59(8)	79.70(10)	81.10(6)
C13–Pt–N2	81.46(8)	81.37(10)	81.04(7)

### Photophysical Properties

**Figure 3:**
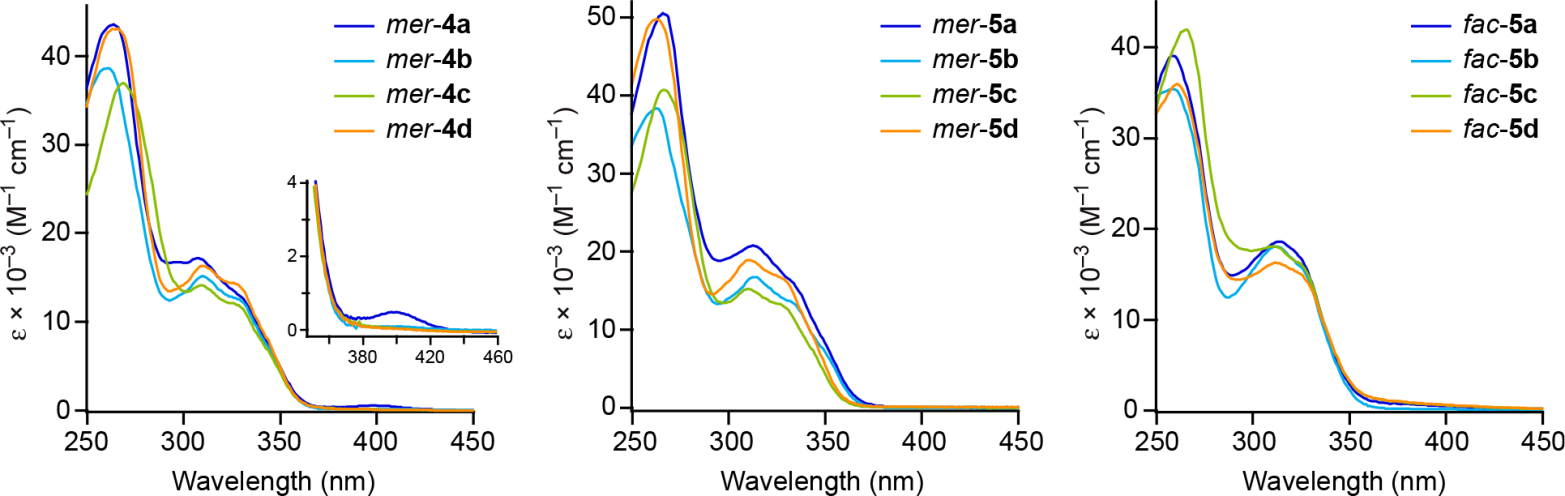
Electronic absorption spectra of *mer*-**4a**–**d**, *mer*-**5a**–**d**, and *fac*-**5a**–**d** in a MeCN solution (ca. 5 × 10^–5^ M) at 298 K.

The electronic absorption spectra of the alkynyl complexes were registered in MeCN solution at 298 K. The data are summarized in Table 2, and the spectra of the carboxylato derivatives are shown in Figure 3. The spectra of *fac*-**6a**–**d** are very similar to those of *fac*-**5a**–**d** and are given in the Supporting Information. All complexes give rise to a structured absorption band with maxima in the 300–330 nm range, which can be mainly ascribed to π–π* (LC) transitions within the tpy ligands.^30,34,37^ Additional features at longer wavelengths can be observed, which have lower molar extinction coefficients and vary depending on the electronic properties of the alkynyl and carboxylato ligands and their geometrical arrangement. A shoulder at around 350 nm is observed for *mer*-**4a**–**d** and *mer*-**5a**–**d**, which is absent in *fac*-**5a**–**d** and *fac*-**6a**–**d**. This difference has been previously noted between *mer* and *fac* isomers of tris-cyclometalated Pt(IV) complexes and attributed to LMCT transitions having lower energies for the *mer* complexes because of their lower dσ* orbital energies.^34,35^ Although the variations along the series are small, these shoulders shift to higher energies as electron withdrawing substituents are introduced on the alkynyl, which is most clearly observed for *mer*-**5a**–**d**. Therefore, they can be assigned to electronic promotions from a π orbital of the alkynyl to a metal dσ* orbital. For *mer*-**4a**, a weak absorption band is observed at 400 nm that we attribute to a LLCT transition from a π orbital of the alkynyl to a π* orbital of one of the tpy ligands; a similar absorption can be discerned for *mer*-**4b** as a tail extending to ca. 450 nm, but not for *mer*-**4c**,**d**, reasonably because it shifts to higher energies as the alkynyl ligands become less electron-donating and is obscured by the LMCT or LC bands. Complexes *fac*-**5a**–**d** present long tails extending to ca. 450 nm that we assign to very broad LLCT transitions (alkynyl-tpy) based on TDDFT calculations (see the computational study).

**Table 2:** Electronic Absorption Data for the Alkynyl Complexes in MeCN Solution (ca. 5 × 10^–5^ M) at 298 K

complex	λ_max_/nm (ε × 10^–3^/M^–1^ cm^–1^)
*mer*-**4a**	263 (43.4), 309 (17.1), 331 (12.5), 344 (7.5, sh), 400 (0.5)
*mer*-**4b**	261 (38.6), 310 (15.1), 331 (12.1), 345 (6.7, sh)
*mer*-**4c**	269 (36.8), 310 (14.0), 331 (11.3), 346 (6.0, sh)
*mer*-**4d**	265 (42.9), 310 (16.2), 330 (13.8), 343 (8.2, sh)
*mer*-**5a**	267 (50.3), 314 (20.7), 337 (14.8), 354 (6.3, sh)
*mer*-**5b**	261 (47.1), 311 (19.1), 330 (15.0), 347 (7.4, sh)
*mer*-**5c**	267 (44.7), 311 (16.7), 329 (14.0), 345 (7.4, sh)
*mer*-**5d**	263 (49.7), 311 (18.8), 331 (15.9), 344 (9.1, sh)
*fac*-**5a**	260 (39.1), 311 (18.4), 329 (15.1)
*fac*-**5b**	260 (35.1), 312 (18.0), 329 (14.4)
*fac*-**5c**	265 (43.9), 311 (18.9), 327 (16.4)
*fac*-**5d**	261 (35.9), 312 (16.2), 328 (14.3)
*fac*-**6a**	260 (42.3), 310 (19.1), 328 (16.5)
*fac-***6b**	258 (51.8), 311 (24.8), 329 (20.5)
*fac*-**6c**	268 (44.0), 310 (17.6), 330 (14.8)
*fac*-**6d**	263 (40.8), 311 (16.8), 328 (15.2)

**Figure 4:**
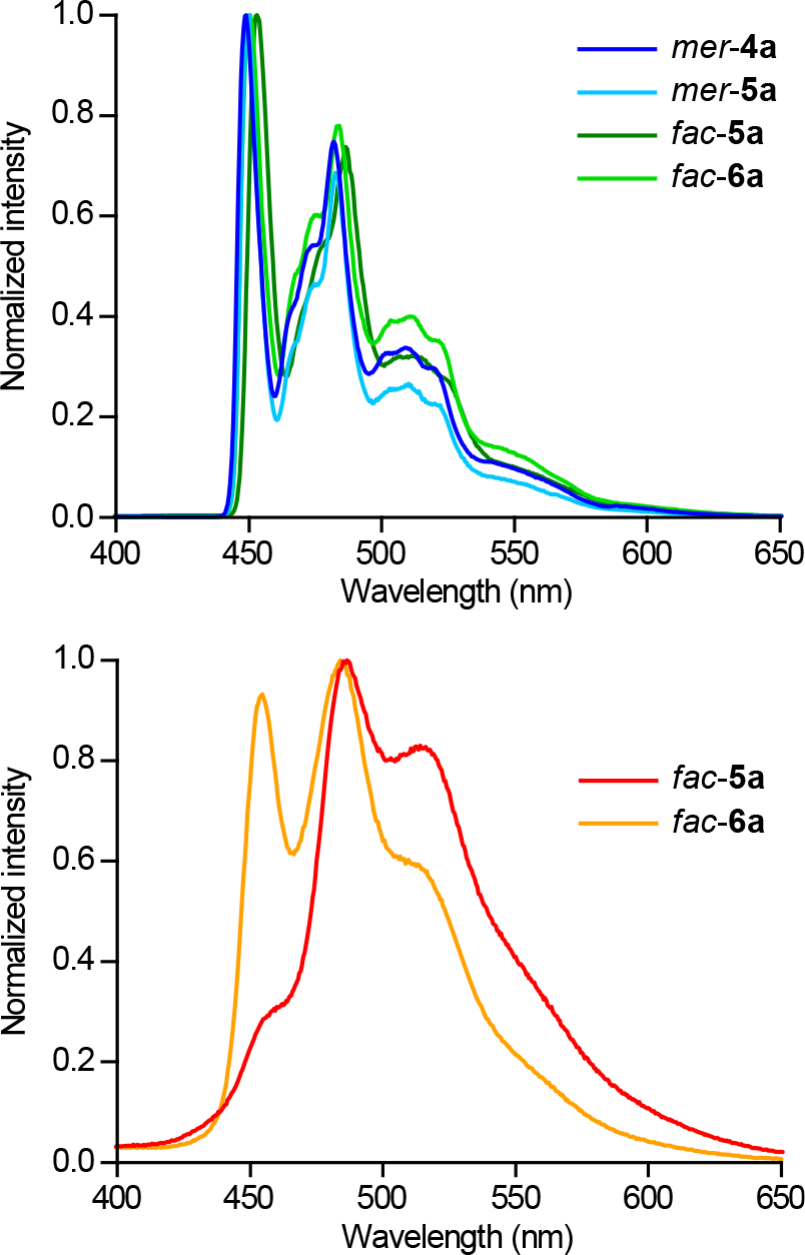
Top: Emission spectra of complexes *mer*-**4a**, *mer*-**5a**, *fac*-**5a**, and *fac*-**6a** in 2-MeTHF at 77 K. Bottom: Emission spectra of *fac*-**5a** and *fac*-**6a** in MeCN at 298 K.

All of the alkynyl complexes were found to be photoreactive under irradiation with UV light in solution at 298 K, with the exceptions of *fac*-**5a** and *fac*-**6a**, which were recovered unreacted after 4 h (see the next section). To characterize their lowest excited state, the emission spectra of *mer*-**4a**, *mer*-**5a**, *fac*-**5a** and *fac*-**6a** were registered in 2-methyltetrahydrofuran (2-MeTHF) frozen glasses at 77 K. Those of *fac*-**5a** and *fac*-**6a** could also be registered in deaerated MeCN solutions at 298 K. The emission data are summarized in Table 3 and selected emission spectra are shown in Figure 4. The complete set of excitation and emission spectra is given in the Supporting Information. Highly structured emissions are observed at 77 K, with lifetimes in the hundreds of microseconds range, revealing an emissive ^3^LC state involving one of the tpy ligands, as typically observed for other cyclometalated Pt(IV) complexes.^30,34,37^ The emissions of *fac*-**5a** and *fac*-**6a** in MeCN at 298 K are very weak, with quantum yields of 0.004 and 0.007, respectively, which can be attributed to the thermal population of geometrically distorted excited states that cause a very effective nonradiative deactivation.

**Table 3:** Emission Data of *mer*-**4a**, *mer*-**5a**, *fac*-**5a**, and *fac*-**6a**

complex	medium (*T*/K)	λ_em_ (nm)^a^	τ (μs)
*mer*-**4a**	2-MeTHF (77)	*449*, 482, 509	240
*mer*-**5a**	2-MeTHF (77)	*450*, 483, 510	280
*fac*-**5a**	2-MeTHF (77)	*453*, 486, 514	163
	MeCN (298)	459, *486*, 515	2.8
*fac*-**6a**	2-MeTHF (77)	*451*, 484, 512	222
	MeCN (298)	455, *484*, 512	1.1

a The most intense peak is italicized.

### Photoinduced Reductive Couplings

**Scheme 2:**
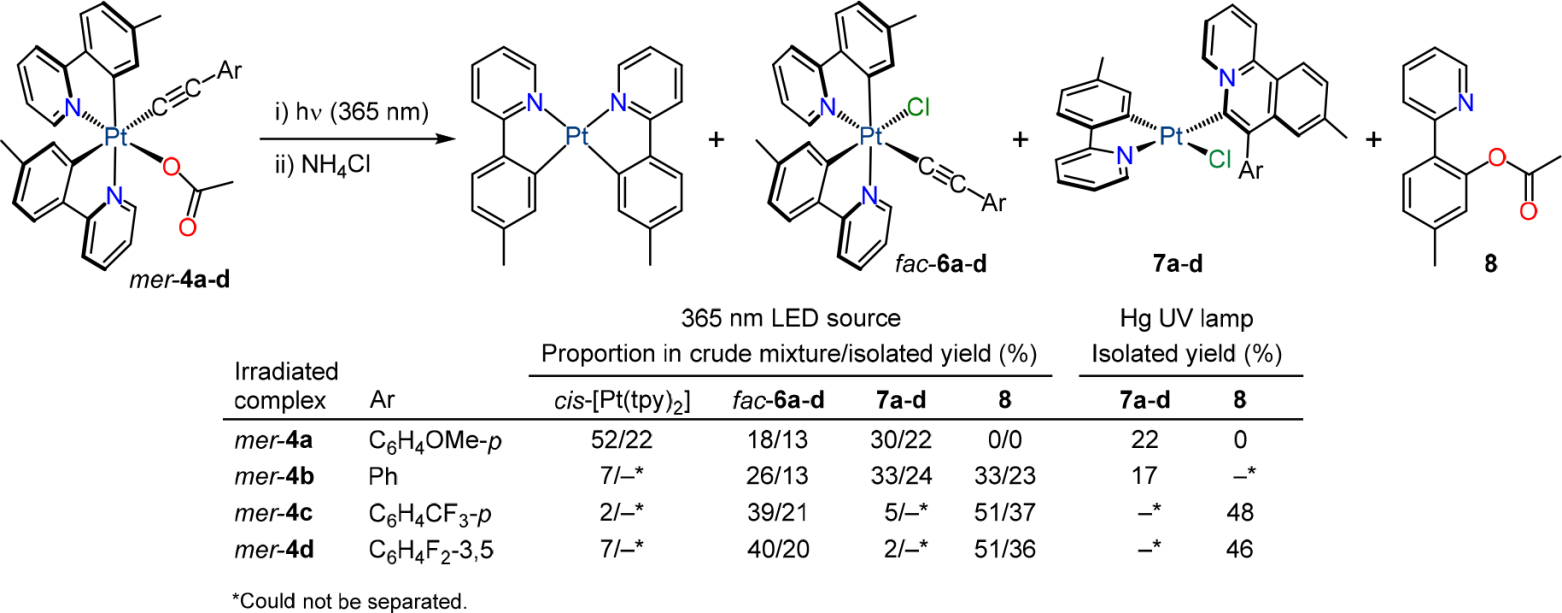
Identified Major Products of the Irradiation of *mer*-**4a**–**d** with UV Light

To characterize their photochemical reactivity, irradiation of MeCN solutions of complexes *mer*-**4a**–**d** was initially carried out with a UV LED source (λ_max_ = 365 nm) at room temperature. The complexes were completely consumed after 2 h, giving mixtures of several products. These mixtures were treated with NH_4_Cl in acetone to replace the acetate with chloride and produce more stable species, which made separation through column chromatography more effective. The ^1^H NMR spectra of the crude mixtures (Figures S31–S34) revealed the presence of variable proportions of *cis*-[Pt(tpy)_2_], the respective *fac*-**6** complex, the Pt(II) complex [PtCl(tpy)(L^1^-Ar)] (**7a**–**d**; where L^1^-Ar is 9-methyl-7-arylbenzo[*a*]quinolizin-5-ium metalated at the 6 position), and 5-methyl-2-(2-pyridyl)phenyl acetate (**8**) (Scheme 2). Small amounts of other products were also present but could not be identified. Isolation of the major products was possible, and the yields approximately correlated with the observed proportions in the crude mixtures (Scheme 2).

**Figure 5:**
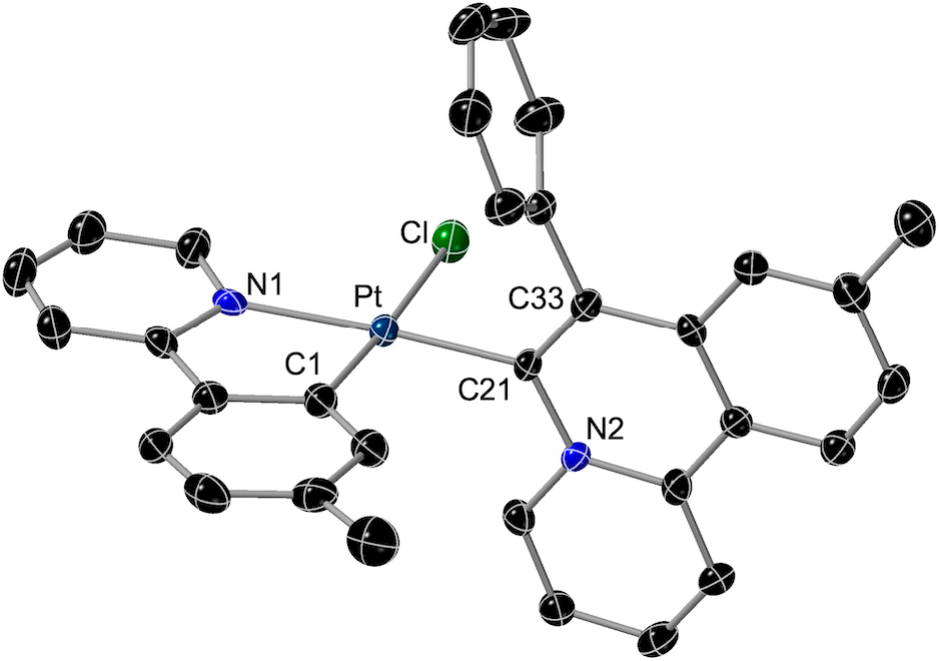
Structure of one of the two independent molecules in the crystal structure of **7b**·1.5CH_2_Cl_2_ (thermal ellipsoids at 50% probability). Hydrogen atoms and solvent molecules are omitted. Selected bond distances (Å) and angles (deg): Pt–C1, 1.983(5); Pt–C21, 2.019(5); Pt–N, 2.083(4); Pt–Cl, 2.4162(13); C1–Pt–C21, 96.3(2); C1–Pt–N1, 81.4(2); C21–Pt–N1, 177.74(19); C1–Pt–Cl, 175.45(16); C21–Pt–Cl, 87.74(15); N1–Pt–Cl, 94.51(13).

The identity of complexes **7a** and **7b** was established thanks to the crystal structure of **7b** (Figure 5), which revealed the metalated benzoquinolizinium ligand in trans to the coordinated N atom of the remaining tpy ligand. This ligand gives rise to a distinctive resonance in the ^1^H NMR spectrum at ca. 11.9 ppm arising from the aromatic proton ortho to the positively charged N atom, which allowed us to identify derivatives **7c** and **7d** in the crude mixtures resulting from the irradiation of *mer*-**4c** and *mer*-**4d**. In addition, these complexes give a shielded aromatic resonance at 6.4 ppm arising from the proton ortho to the metalated carbon of the tpy ligand, which is affected by the ring current of the benzoquinolizinium (Figures S25 and S26).

The above results demonstrate that at least three different photoinduced reductive processes occur in addition to the isomerization to the *fac* geometry. The formation of *cis*-[Pt(tpy)_2_] probably involves a reduction of the metal by the alkynyl group, although the ensuing organic product could not be detected. Complexes **7a**–**d** result from an annulation reaction involving the C_α_ and C_β_ atoms of the alkynyl and one of the tpy ligands. Related inter- and intramolecular annulations between alkynyls and 2-arylpyridines mediated or catalyzed by transition metal ions have been reported to produce quinolizinium ions, which are important as key structural motifs of many natural alkaloids and bioactive compounds.^47−50^ The formation of compound **8** entails C–O coupling between one of the tpy ligands and the acetato ligand. This reaction should also produce a Pt(II) complex with a cyclometalated tpy ligand, but it could not be identified. Although several examples of C(sp^3^)–O reductive couplings from Pt(IV) methyl complexes have been described, C(sp^2^)–heteroatom couplings from Pt(IV) aryl complexes are challenging^51,52^ and only recently have the first examples of Ar–X (X = OAr, SAr, NRR′) reductive elimination reactions from Pt(IV) complexes been reported, which proceed under thermal conditions.^53^

The electronic properties of the arylacetylide clearly affect the relative proportions in which the different photoinduced processes occur. Thus, the more electron-rich alkynyls favor the formation of *cis*-[Pt(tpy)_2_] and complexes **7**, suggesting that the responsible reactive excited states involve electronic promotions from molecular orbitals primarily localized on the alkynyl. Conversely, upon introduction of electron-withdrawing substituents on the aryl group, the isomerization to the *fac* geometry and the C–O reductive coupling leading to **8** predominate, meaning that these processes are likely triggered by excited states affecting the acetato ligand.

Irradiations of the complete set of *mer*-**4** complexes were also systematically performed using diluted MeCN solutions (ca. 2 × 10^–4^ M) in a thermostated (298 K) UV photoreactor equipped with a 150 W medium-pressure Hg lamp. Under these conditions, the complete consumption of the alkynyl complexes was observed within ca. 1 h. In the cases of *mer*-**4a** and *mer*-**4b**, Pt(II) complexes **7a** and **7b** were obtained in 22 and 17% yields, respectively, after the replacement of acetate for chloride and purification through column chromatography. No other products could be isolated from the reaction mixtures, probably as a consequence of photolysis due to highly energetic emissions of the employed lamp. In contrast, compound **8** was exclusively isolated from the irradiation of *mer*-**4c** and *mer*-**4d** in 48 or 46% yield, respectively. We hypothesize that higher yields in **8** using this lamp as compared to the 365 nm LED source could result from C–O reductive couplings occurring from the produced isomers *fac*-**4c** or *fac*-**4d** upon the absorption of high-energy UV light.

**Scheme 3:**
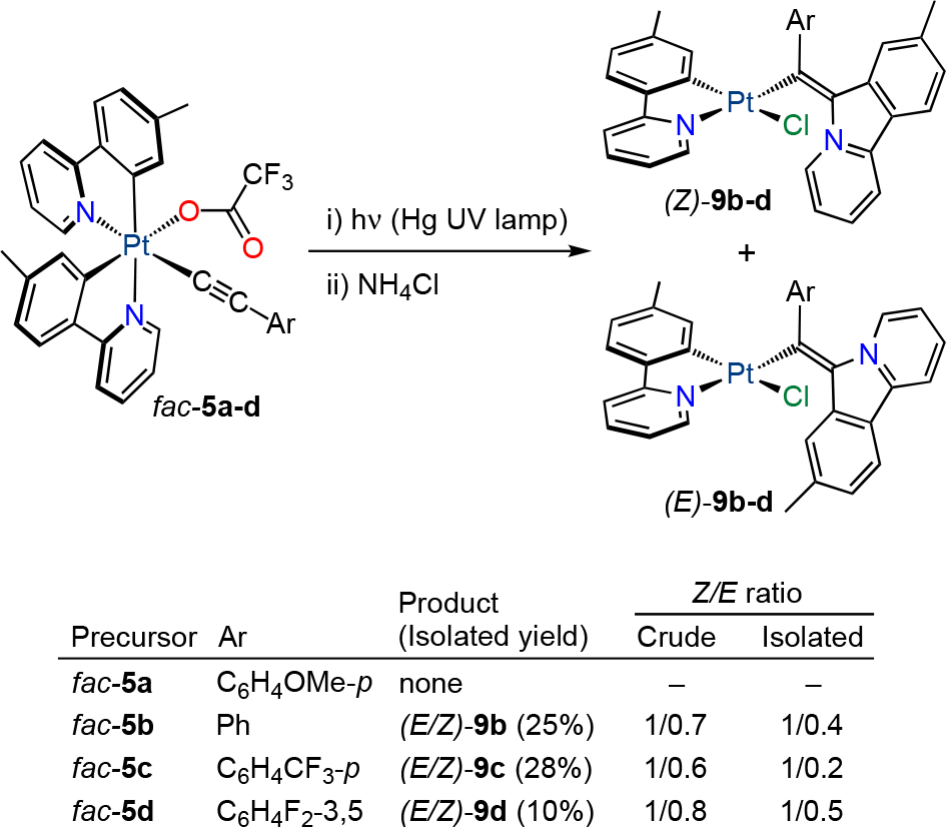
Results of the Irradiation of *fac*-**5a**–**d** with UV Light

Complexes *fac*-**5a**–**d** were also subjected to UV irradiation in the thermostated photoreactor at 298 K. Surprisingly, *fac*-**5a** was recovered unreacted after 4 h, whereas complexes *fac*-**5b**–**d** were completely transformed into mixtures that contained two major compounds, as deduced from the ^1^H NMR spectra of the crude residues. After the exchange of trifluoroacetate for chloride and purification through column chromatography, Pt(II) complexes of the type [PtCl(tpy)(L^2^-Ar)] were identified, where L^2^-Ar is an 8-methyl-6*H*-pyrido[2,1-*a*]isoindol-5-ium-6-ylidene)(aryl)methanide ligand, which were present as a mixture of *Z* and *E* isomers [(*Z*/*E*)-**9b**–**d**; Scheme 3]. Therefore, a reductive coupling between the alkynyl and one of the tpy ligands also occurred but involving only the C_α_ atom of the alkynyl. Isolated yields ranged from 10 to 28%. No other products could be identified in the reaction mixtures. The *Z* isomer was predominant in both the crude and the isolated mixtures.

**Figure 6:**
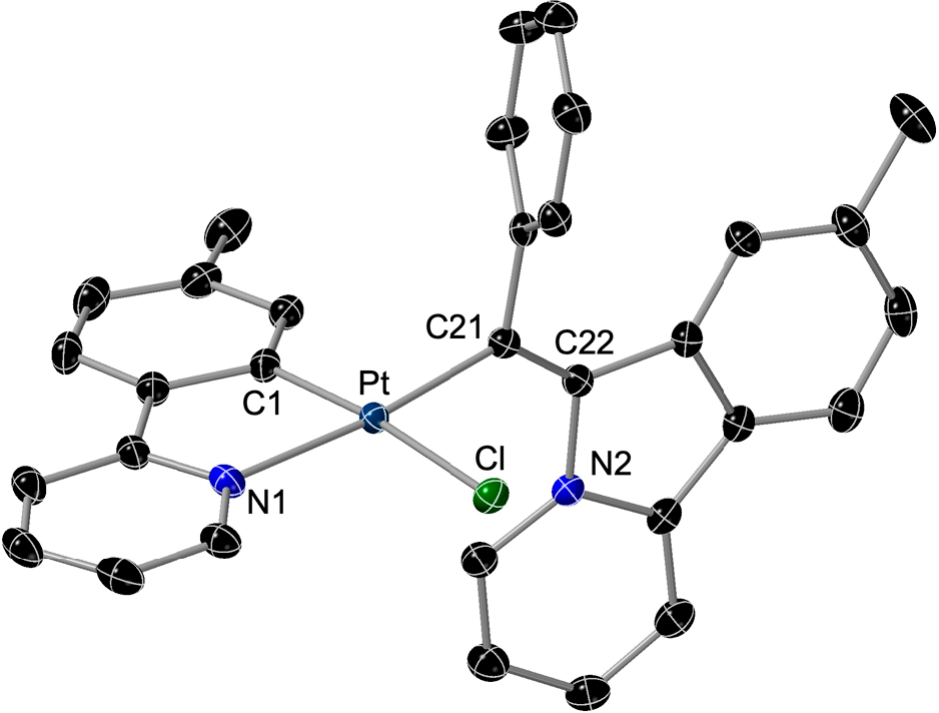
Structure of complex (*Z*)-**9b** (thermal ellipsoids at 50% probability). Hydrogen atoms are omitted. Selected bond distances (Å) and angles (deg): Pt–C21, 1.978(2); Pt–C1, 1.991(2); Pt–N1 2.089(2); Pt–Cl, 2.4135(6); C21–Pt–C1, 95.36(9); C21–Pt–N1, 173.93(9); C1–Pt–N1, 80.85(9); C21–Pt–Cl, 90.41(6); C1–Pt–Cl, 174.02(7); N1–Pt–Cl, 93.54(6).

The crystal structure of (*Z*)-**9b** was determined by X-ray diffraction and is shown in Figure 6. The L^2^-Ph ligand is situated trans to the coordinated N atom of the tpy ligand, and the pyridoisoindolium fragment is approximately perpendicular to the coordination mean plane. The distinctive feature in the ^1^H NMR spectra of derivatives **9** is the resonance of the proton ortho to the positively charged N atom, which appears at 13.3 ppm for the *Z* isomers, whereas it is upfield-shifted to 10.3 ppm for the *E* isomers because it is directed toward the shielding region of the aryl ring.

Irradiations of complexes *fac*-**6a** and *fac*-**6b** were also conducted at 298 K by using the photoreactor to evaluate the effect of introducing the chlorido ligand. Like *fac*-**5a**, complex *fac*-**6a** did not undergo any transformation. Complex *fac*-**6b** directly produced (*Z*/*E*)-**9b**, but the reaction was much slower compared to complexes *fac*-**5b**–**d** and did not complete after 5 h, suggesting that these reductive couplings are facilitated by the presence of the relatively labile trifluoroacetato ligand in *fac*-**5b**–**d** and involve a dissociation step.

### Computational Study and Assignment of Reactive Excited States

**Figure 7:**
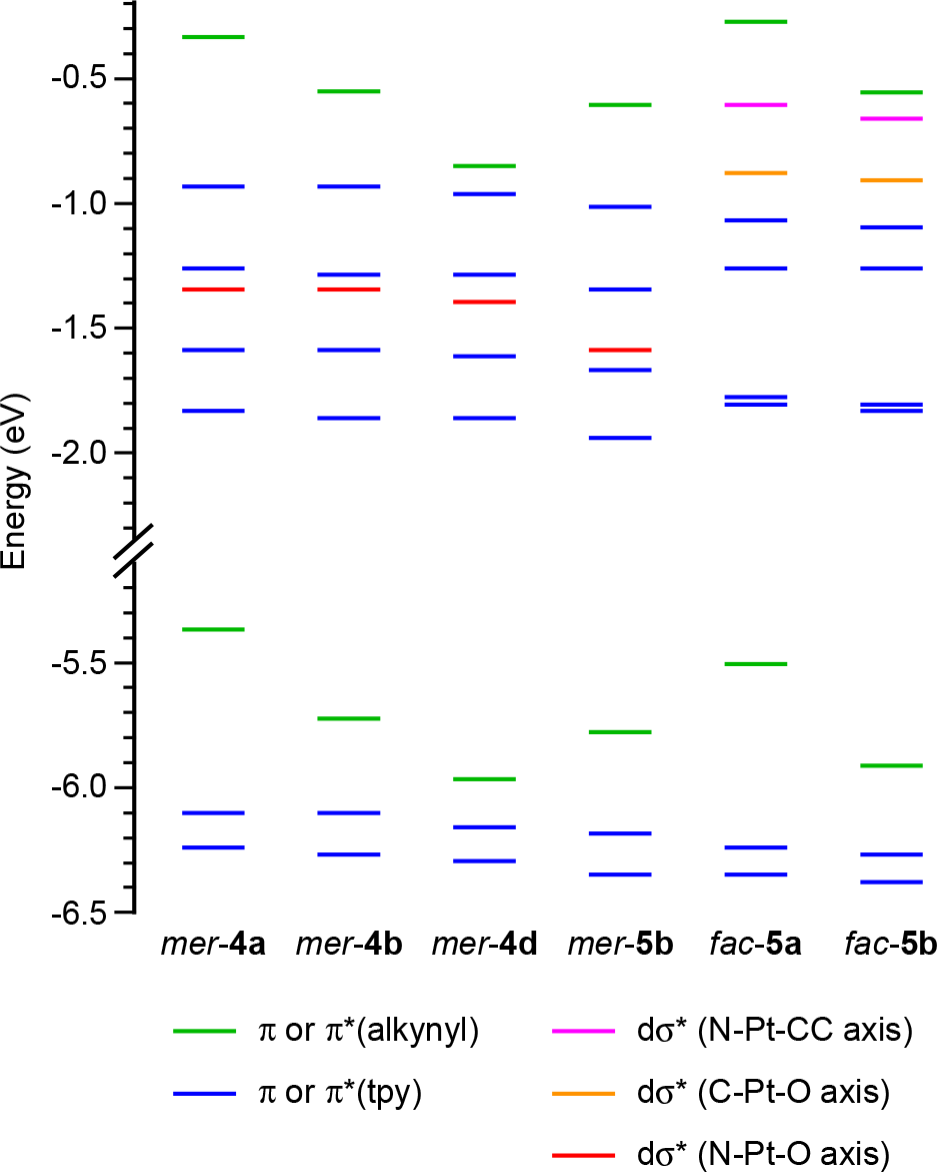
Molecular orbital energy diagram from DFT calculations.

To get an understanding of the photophysical properties and photochemical reactivity of the alkynyl complexes, DFT and TDDFT calculations were performed for *mer*-**4a**, *mer*-**4b**, *mer*-**4d**, *mer*-**5b**, *fac*-**5a**, and *fac*-**5b**. Complete details are given in the Supporting Information. A comparison between the free energies of *mer*-**5b** and *fac*-**5b** shows that the *fac* geometry leads to higher thermodynamic stability (Table S24). In all cases, the highest occupied molecular orbital (HOMO) is a π orbital of the alkynyl ligand (see Figure 7 for an orbital energy diagram). As expected, complexes *mer*-**4a** and *fac*-**5a** present the highest HOMO energies among the calculated series due to the presence of the electron-donating methoxy substituent on the arylacetylide. The HOMO–1 and HOMO–2 are π orbitals of each of the tpy ligands, which remain at very similar energies along the series. Analogously, the lowest unoccupied molecular orbital (LUMO) and LUMO+1 are π* orbitals mainly localized on each of the tpy ligands. The *mer* complexes present a low-lying dσ* orbital (LUMO+3) mainly distributed along the N–Pt–O axis, which has a lower energy for *mer*-**5b**, reasonably because of the weaker σ-donating ability of the trifluoroacetate relative to the acetate. The *fac* complexes present two dσ* orbitals mainly distributed along the C–Pt–O or N–Pt–CC axes, with higher energies with respect to the *mer* complexes, indicating that the *fac* geometry leads to stronger ligand-field splitting.

The TDDFT calculations predict ^1^LLCT [π(alkynyl) → π*(tpy)] and ^1^LMCT [π(alkynyl) → dσ*] transitions (or admixtures thereof) as the lowest-energy singlet excitations for the *mer* complexes. Oscillator strengths are very low for the ^1^LLCT transitions, whereas they are noticeable for transitions with significant LMCT character. Among the *mer* complexes, *mer*-**4a** and *mer*-**5b** present the lowest energies for ^1^LMCT transitions because of the higher energy of the π(alkynyl) orbital in *mer*-**4a** or the lower energy of the dσ* orbital in *mer*-**5b**, whereas *mer*-**4b** and *mer*-**4d** present increasingly higher energies for these transitions because of the decreasing energies of their π(alkynyl) orbitals. These results are consistent with an assignment of the shoulders around 350 nm in the absorption spectra of the *mer* complexes to ^1^LMCT transitions, whereas the weaker features at longer wavelengths correspond to ^1^LLCT transitions. In the cases of the *fac* complexes, several ^1^LLCT [π(alkynyl) → π*(tpy)] excitations are predicted as the lowest-energy singlet excitations, which may be responsible for the long tails observed in the experimental absorption spectra. In all cases, ^1^LC transitions involving the tpy ligands are predicted as the most intense singlet excitations in the 310–325 nm range. According to this analysis, irradiation with 365 nm light should populate ^1^LLCT and ^1^LMCT states in the cases of the *mer* complexes or ^1^LLCT states in the cases of the *fac* complexes, whereas by using the Hg UV lamp, ^1^LC(tpy) states, or even higher-lying excited states should be effectively populated.

**Figure 8:**
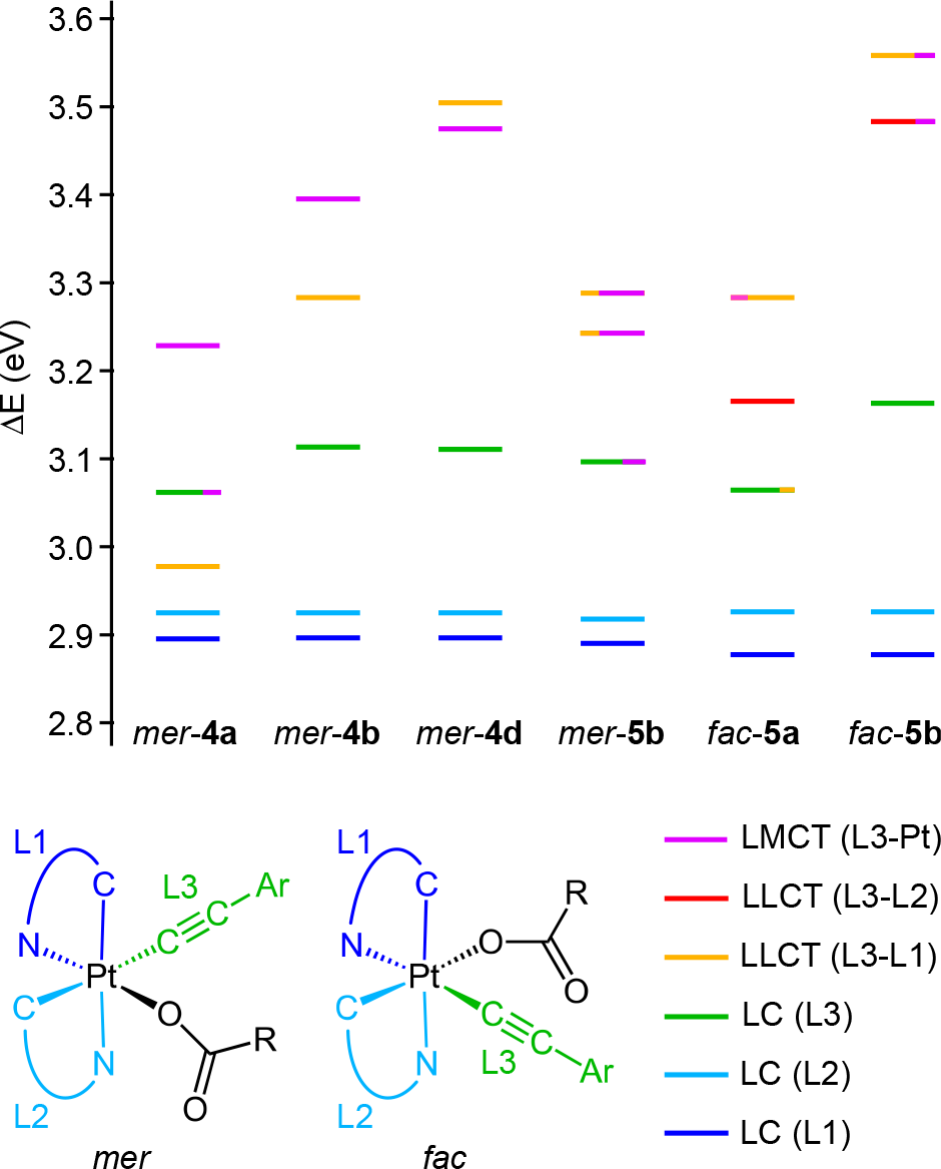
Energy diagram showing the lowest vertical triplet excitations from TDDFT calculations at the ground-state geometries and their assignment.

The observed photochemical reactivity is expected to be primarily triggered by triplet excited states because intersystem crossing to the triplet manifold should occur after photoexcitation due to the spin–orbit coupling effects induced by the metal. The TDDFT results show that, in all cases, the first two triplet excitations (T_1_ and T_2_) correspond to ^3^LC(tpy) transitions involving each of the tpy ligands (Figure 8). A ^3^LC(alkynyl) transition is also predicted as T_3_ excitation (T_4_ for *mer*-**4a**). None of the respective excited states is expected to induce photochemical reactivity, because they are usually stable and long-lived. In fact, the observed emissions can be clearly attributed to the lowest ^3^LC(tpy) state. Therefore, higher-lying excited states should be examined, which could be thermally populated from the lowest triplet state. Complexes *mer*-**4a**, *mer*-**4b** and *mer*-**4d** present ^3^LLCT [π(alkynyl) → π*(tpy)] and ^3^LMCT [π(alkynyl) → dσ*] excitations, whose energies increase as the alkynyl becomes less electron-donating (Figure 8). Their relative ordering also changes, with the ^3^LLCT excitation being lower in energy than the ^3^LMCT excitation for *mer*-**4a** and *mer*-**4b**, whereas the reverse situation is found for *mer*-**4d**. In the three cases, these states lie within less than 0.6 eV above the lowest ^3^LC state and therefore thermal population is expected to be possible at room temperature.

In view of the observed photochemical transformations from complexes *mer*-**4** leading to reduction of the metal or isomerization to the *fac* geometry, it is likely that they all proceed through different competitive pathways initiated by the ^3^LMCT [π(alkynyl) → dσ*] state, some of which would involve ligand dissociation or reductive couplings. Reduction to *cis*-[Pt(tpy)_2_] is probably most favored for *mer*-**4a** because of the stronger electron-donating character of the 4-methoxyphenyl substituent. Since the dσ* orbital involved in the ^3^LMCT state is distributed along the N–Pt–O axis, the rupture of the Pt–O bond could be promoted. Based on the behavior of the trifluoroacetato derivatives, the isomerization of complexes *mer*-**4** probably involves a heterolytic dissociation of the acetate to produce a cationic pentacoordinate intermediate. The C–O coupling could be triggered by homolytic cleavage of the Pt–O bond or proceed through a concerted mechanism.

**Scheme 4:**
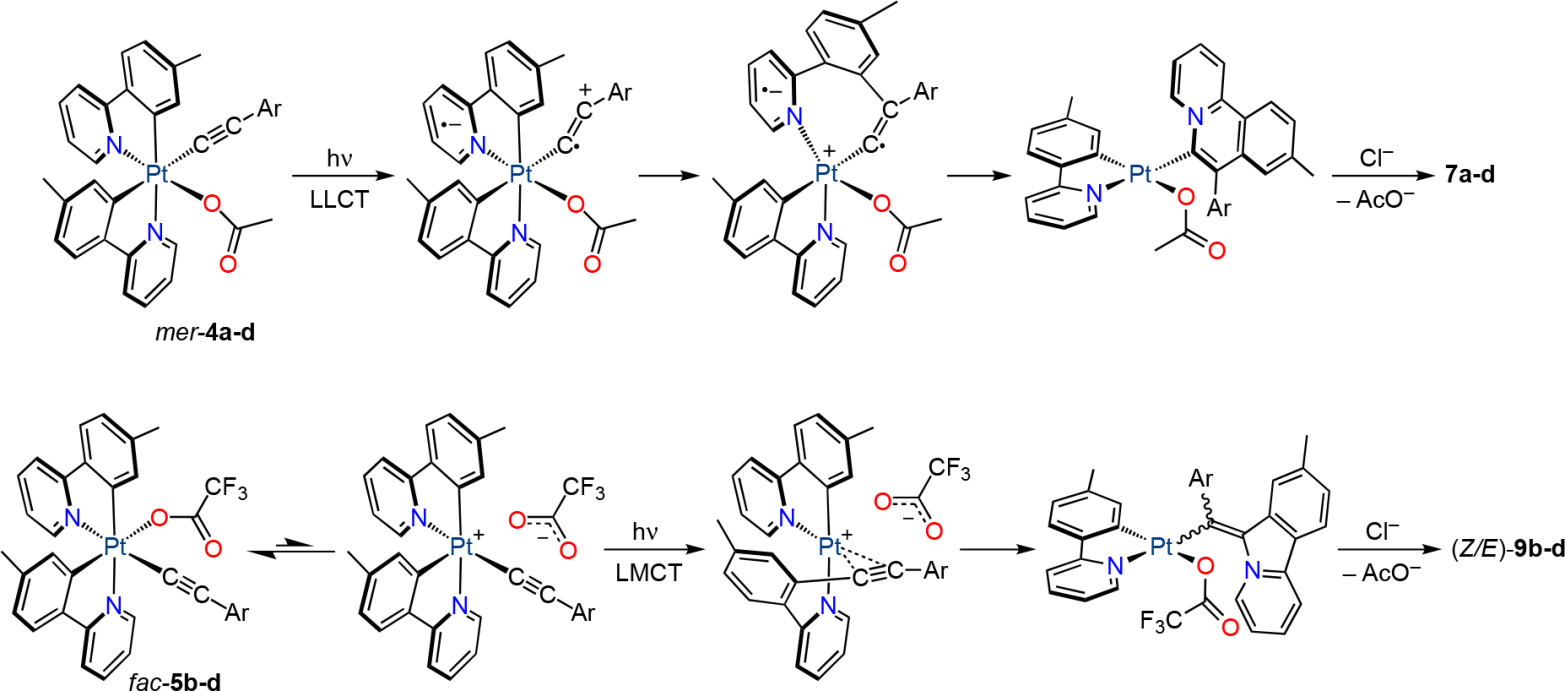
Postulated Reaction Sequences for the Formation of **7a**–**d** and (*Z/E*)-**9b**–**d**

Given that the highest proportions of complexes **7** are obtained from *mer*-**4a** and *mer*-**4b**, an alternative hypothesis is that their formation is initiated by the ^3^LLCT [π(alkynyl) → π*(tpy)] state, which should be more easily populated in these derivatives because it lies at a lower energy. This state involves an electron transfer from the alkynyl π system to a π* orbital of the tpy ligand, which could be followed by nucleophilic attack of the metalated aryl of the tpy ligand to the C_β_ atom of the alkynyl and reductive N–C_α_ coupling to produce the benzoquinolizinium fragment (Scheme 4). The different behaviors of *mer*-**4c** and *mer*-**4d**, for which the isomerization and C–O coupling processes predominate, would then be explained by the preferential population of the ^3^LMCT state as compared to the ^3^LLCT state in these cases.

In the case of *mer*-**5b**, the T_4_ and T_5_ excitations are ^3^LLCT/LMCT admixtures with predominant LMCT character, which are expected to cause heterolytic dissociation of the trifluoroacetato ligand, leading to isomerization to *fac*-**5b**. Reasonably, the trifluoroacetate is more prone to heterolytic dissociation compared to the acetate, which could explain why these derivatives preferentially undergo photoisomerization and do not produce the C–O coupling between the carboxylate and one of the tpy ligands.

Complexes *fac*-**5a** and *fac*-**5b** present ^3^LLCT states involving electronic promotions from the alkynyl to the π* orbitals of the tpy ligands, which have lower energies for *fac*-**5a** because of the higher energy of the alkynyl-based π orbital. In view of their energies, these states are expected to be thermally accessible from the lowest ^3^LC state, but it is clear that they do not directly trigger the reductive process leading to complexes **9** because this transformation is not observed for the most favorable case (*fac*-**5a**), where they probably produce just geometrical distortions that cause nonradiative deactivation of the lowest ^3^LC(tpy) state. The ^3^LLCT states in *fac*-**5b** have some LMCT character mixed in, suggesting that reductive processes might be more favorable. We speculate that the observed reaction occurs through a photoreactive pentacoordinate intermediate with accessible ^3^LMCT states, requiring previous dissociation of the trifluoroacetato ligand. This would be consistent with the observation that the process is much slower for chlorido complex *fac*-**6b**. The photoinduced step is possibly a reductive C–C coupling involving the C_α_ atom of the alkynyl and the metalated carbon of a tpy ligand, which would be followed by decoordination of the pyridine ring and cyclization to give the isoindolium fragment (Scheme 4).

## Conclusions

Bis-cyclometalated Pt(IV) alkynyl complexes of the types *mer/fac*-[Pt(tpy)_2_(O_2_CR)(CCAr)] and *fac*-[Pt(tpy)_2_Cl(CCAr)] have been synthesized, and their photophysical properties and photochemical reactivity have been examined. The *mer* derivatives bearing a carboxylato ligand are photoreactive under UV light irradiation, undergoing different transformations depending on the electronic properties of both the carboxylato and alkynyl ligands. Thus, irradiation of the acetato complexes may lead to reduction to *cis*-[Pt(tpy)_2_], isomerization to the *fac* geometry, reductive annulations between one of the tpy ligands and the C_α_ and C_β_ atoms of the alkynyl to give benzoquinolizinium derivatives, or C–O couplings between the acetato ligand and one tpy. The more electron-rich alkynyls favor the reduction to *cis*-[Pt(tpy)_2_] and annulations, whereas the introduction of electron-withdrawing substituents favors isomerization and C–O couplings. In contrast, irradiation of the trifluoroacetato complexes led exclusively to isomerization to the *fac* geometry. The *fac* derivatives bearing the most electron-rich alkynyl were found to be photostable, but the others underwent reductive C–C couplings involving the alkynyl C_α_ atom and one of the tpy ligands to give pyridoisoindolium derivatives.

An analysis of the nature and relative energies of the lowest triplet excitations of the *mer* complexes through TDDFT, combined with the proportions in which the different reductive processes occur, led us to postulate ^3^LLCT states as responsible for the annulations, whereas ^3^LMCT states would trigger reduction to *cis*-[Pt(tpy)_2_], isomerization, or C–O couplings. The C–C couplings observed for the *fac* complexes probably occur through photoreactive pentacoordinate intermediates. The cyclometalated ligands play a crucial role in the observed reactivities because they provide a highly energetic ^3^LC state, from which the reactive excited states can be thermally populated.

In brief, this work demonstrates that reductive C–C or C–heteroatom couplings can be photochemically induced using Pt(IV) complexes bearing cyclometalated 2-arylpyridines and provides valuable knowledge on the behavior of different reactive excited states that could help to develop new photoinduced processes with potential applications in synthesis.

## Supporting Information Available

Experimental details, characterization data, X-ray structure determinations, crystallographic data, NMR spectra, excitation and emission spectra, and computational methods and data (PDF)
