# Supplementary material for: Photoinduced Reductive C–C and C–Heteroatom Couplings from Bis-cyclometalated Pt(IV) Alkynyl Complexes
Source: Inorg Chem. 2023 Aug 24;62(35):14411–21. doi: 10.1021/acs.inorgchem.3c02162 (PMC10481375; doi:10.1021/acs.inorgchem.3c02162)
Supplement: Supplementary file 1 — ic3c02162_si_001.pdf [file ic3c02162_si_001.pdf]

SUPPORTING INFORMATION

Photoinduced Reductive C–C and C–Heteroatom Couplings from Bis-cyclometalated Pt(IV) Alkynyl Complexes

Juan Carlos López-López,[†] *Delia Bautista*[‡] *and Pablo González-Herrero*[*,†]

[†]Departamento de Química Inorgánica, Facultad de Química, Universidad de Murcia, Campus de Espinardo, 19, 30100 Murcia, Spain.

[‡]Área Científica y Técnica de Investigación, Universidad de Murcia, Campus de Espinardo, 21, 30100 Murcia, Spain.

*Email: pgh@um.es.

Contents:

1. Experimental details 2
   1.1. General considerations and materials 2
   1.2. Irradiations 2
   1.3. Spectroscopic and analytical methods 2
   1.4. Synthesis and characterization data of new compounds 2
   1.5. X-ray structure determinations 11
2. Crystal structures of 3, 3' and *mer*-4b 13
3. NMR spectra of new compounds 16
4. NMR spectra of crude reaction mixtures 43
5. Excitation and emission spectra 45
6. Computational methods 46
7. Computational data 47
   7.1. Complex *mer*-4a 47
   7.2. Complex *mer*-4b 51
   7.3. Complex *mer*-4d 55
   7.4. Complex *mer*-5b 59
   7.5. Complex *fac*-5a 63
   7.6. Complex *fac*-5b 67
8. References 75

1. Experimental details

1.1. General considerations and materials

Unless otherwise noted, all reactions were carried out at room temperature using extra-dry MeCN and flame-dried glassware under an N_2 atmosphere. Synthesis grade Et_2O and CH_2Cl_2 were degassed and dried using a Pure Solv MD-5 solvent purification system from Innovative Technologies, Inc. Other solvents were used as received. Complex *cis*-[Pt(tpy)$_2$] was prepared following a published procedure.[1] All other reagents were obtained from commercial sources.

1.2. Irradiations

Irradiations with 365 nm UV light were performed in flat-bottom, 20 mL Carius tubes (∅ = 20 mm) made of borosilicate glass and fitted with a PTFE vacuum stopcock, which were placed on top of individual LED emitters (LED Engin LuxiGen™ LZ1-10UV0R-0000) fixed to an aluminum heat sink and cooled by a fan. The radiant flux at the bottom of the tube was *ca.* 1360 mW based on technical specifications. The thermostated photoreactor was a UV-Consulting Peschl photoreactor, model UV-RS-1, equipped with a 150 W medium-pressure Hg immersion UV lamp (TQ 150; main emission wavelengths: 254, 265, 303, 312, 365 nm), a 400 mL reaction vessel with magnetic circulation pump, and a quartz cooling jacket connected to a PolyScience circulation chiller.

1.3. Spectroscopic and analytical methods

NMR spectra were recorded on Bruker Avance 300, 400, or 600 MHz spectrometers at 298 K. ^{1}H and $^{13}C\{^{1}H\}$ NMR spectra were referenced using residual signals of non-deuterated solvent and are given in ppm downfield from tetramethylsilane. ^{19}F NMR spectra were referenced against external $CFCl_3$. Elemental analyses were carried out with a LECO CHNS-932 microanalyzer. UV-vis absorption spectra were recorded on a Perkin-Elmer Lambda 750S spectrophotometer. Excitation and emission spectra were recorded on a Jobin Yvon Fluorolog 3-22 spectrofluorometer. The measurements in solution at room temperature were carried out using 10 mm quartz fluorescence cells. For the low-temperature measurements, 5 mm quartz NMR tubes with solutions of the complexes were placed in a liquid nitrogen Dewar with quartz windows. Emission lifetimes were determined using an IBH FluoroHub controller in MCS mode and the Fluorolog's FL-1040 phosphorimeter pulsed xenon lamp as excitation source ($\tau > 10$ μs) or in TCSPC mode using a pulsed NanoLED source ($\tau < 10$ μs); the estimated uncertainty is ±10% or better. Emission quantum yields were determined using a Hamamatsu C11347 Absolute PL Quantum Yield Spectrometer; the estimated uncertainty is ±5% or better.

1.4. Synthesis and characterization data of new compounds

[Pt(tpy)$_2$(OAc)$_2$] (1). To a solution of *cis*-[Pt(tpy)$_2$] (160 mg, 0.3 mmol) in CH_2Cl_2 (3 mL) was added PhI(OAc)$_2$ (0.33 mmol) and the mixture was stirred for 16 h. The solvent was evaporated under reduced pressure and the residue was crystallized from acetone/Et_2O to produce a white solid, which was collected by filtration, washed with Et_2O (3 × 2 mL) and vacuum-dried to give **1**. Yield: 162 mg (83%). ^{1}H NMR (600 MHz, CD_2Cl_2): δ 9.26 (d, J = 5.5 Hz, H), 8.07–8.02 (m, 1 H), 8.00 (d, J = 8.2 Hz, 1 H), 7.72–7.65 (m, 3 H), 7.56 (d, J = 7.9 Hz, 1H), 7.53–7.51 (m, 2 H), 7.47 (d with satellites, J_{PtH} = 32.8 Hz, Hz, J_{HH} = 6.0 Hz, 1 H), 7.25 (d, J = 8.2 Hz, 1 H), 6.84 (d, J = 7.9 Hz, 1 H), 6.82 (dt, J = 6.1 Hz, J = 2.8 Hz, 1 H), 6.04 (s with satellites, J_{PtH} = 38.0 Hz, 1 H), 2.57 (s, 3 H), 2.02 (s, 3 H), 1.90 (s, 3 H), 1.80 (s, 3 H). $^{13}C\{^{1}H\}$ NMR (150.8

MHz, CD_2Cl_2): δ 176.9 (C), 174.8 (C), 165.9 (C), 162.2 (C), 150.6 (CH), 149.0 (CH), 145.7 (C), 142.9 (C), 141.3 (C), 140.5 (CH), 140.4 (CH), 140.1 (C), 139.1 (C), 133.5 (J_{PtC} = 35.2 Hz, CH), 130.8 (C), 129.6 (CH), 127.5 (CH), 126.9 (CH), 124.9 (J_{PtC} = 32.7 Hz, CH), 124.7 (J_{PtC} = 52.5 Hz, CH), 123.2 (CH), 122.1 (J_{PtC} = 32.1 Hz, CH), 119.8 (CH), 119.7 (CH), 25.1 (CH_3), 24.0 (CH_3), 22.6 (CH_3), 21.9 (CH_3). Anal. Calcd for $C_{28}H_{26}N_2O_4Pt$: C 50.20, H 3.96, N 4.13. Found: C 50.31, H 4.07, N 4.12.

[Pt(tpy)$_2$(O$_2$CCF$_3$)$_2$] (2). A solution of PhI(O_2CCF_3)$_2$ (0.21 mmol) in Et_2O (5 mL) was added dropwise to a suspension of *cis*-[Pt(tpy)$_2$] (100 mg, 0.19 mmol) in Et_2O (10 mL). The mixture was stirred for 3 h and the solvent was evaporated under reduced pressure. The residue was treated with $CHCl_3$ (1 mL) and Et_2O (10 mL), whereupon a white solid precipitated, which was collected by filtration, washed with Et_2O (3 × 2 mL) and vacuum-dried to give **2**. Yield: 125 mg (87%). ^{1}H NMR (600 MHz, CD_2Cl_2): δ 9.24 (ddd, J = 5.5, 1.6, 0.8 Hz, 1H), 8.13 (ddd, J = 8.2, 7.5, 1.6 Hz, 1H), 8.07 (d, J = 8.3 Hz, 1H), 7.84–7.76 (m, 2H), 7.66 (s with satellites, J_{PtH} = 7.0 Hz, 1H), 7.64–7.60 (m, 1H), 7.59 (d, J = 7.8 Hz, 1H), 7.56 (d, J =8.1 Hz, 1H), 7.36 (ddd, J = 6.5, 1.4, 0.9 Hz, 1H), 7.31 (ddd, J = 7.9, 1.6, 0.7 Hz, 1H), 6.96–6.88 (m, 2H), 6.08 (s with satellites, J_{PtH} = 39.4 Hz, 1H), 2.57 (s, 3H), 2.07 (s, 3H). ^{13}C{^{1}H} NMR (150.8 MHz, CD_2Cl_2): δ 167.0 (C), 161.6 (q, J_{CF} = 36.7 Hz, C), 161.3 (C), 161.0 (q, J_{CF} = 37.0 Hz, C), 149.7 (CH), 147.9 (CH), 145.4 (C), 143.9 (C), 142.5 (C), 141.6 (CH), 141.5 (CH), 139.1 (C), 138.7 (J_{PtC} = 30.1 Hz, C), 133.4 (J = 33.6 Hz, CH), 129.6 (CH), 128.3 (CH), 128.2 (CH), 127.4 (J_{PtC} = 801.3 Hz, C), 125.4 (J_{PtC} = 30.0 Hz, CH), 125.3 (J_{PtC} = 30.0 Hz), 124.2 (CH), 123.2 (J_{PtC} = 33.7 Hz), 120.7 (CH), 120.5 (CH), 116.8 (q, J_{CF} = 294.2 Hz, C), 116.5 (q, J_{CF} = 294.2 Hz, C), 22.7 (CH_3), 22.0 (CH_3). ^{19}F NMR (377 MHz, CD_2Cl_2): δ –73.2 (s, J_{PtF} = 11.3 Hz, 3F), –73.5 (s, 3F). Anal. Calcd for $C_{28}H_{20}F_6N_2O_4Pt$: C 44.39, H 2.66, N 3.70. Found: C 44.68, H 2.66, N 3.63.

[{Pt(tpy)$_2$}$_2$(μ-O$_2$CCF$_3$-κ*O*:κ*O'*)][(CF$_3$CO$_2$)$_2$H] (3). A solution of PhI(O_2CCF_3)$_2$ (40 mg, 0.094 mmol) in CH_2Cl_2 (5 mL) at –16 °C (ice and salt bath) was added dropwise to a solution of *cis*-[Pt(tpy)$_2$] (50 mg, 0.094 mmol) in CH_2Cl_2 (5 mL) at –90 °C (isopropanol bath with liquid N_2) under an N_2 atmosphere and the resultant solution was stirred for 15 min and then allowed to warm to room temperature. The solvent was removed under reduced pressure and the residue was triturated with hexane to give **3** as a pale green precipitate, which was collected by filtration, washed with hexane (5 mL) and vacuum-dried. Yield: 54 mg (90%). ^{1}H NMR (600 MHz, CD_2Cl_2): δ 8.31 (d, J = 5.6 Hz, 1H), 8.15 (s with satellites, J_{PtH} = 34.1 Hz, 1H), 8.06 (d, J = 5.1 Hz, 1H), 8.03 (dt, J = 7.8, 1.5 Hz, 1H), 7.9 (d, J = 8.1 Hz, 1H), 7. 81 (d, J = 8.1 Hz, 1H), 7.80–7.75 (m, 2H), 7.71 (d, J = 8.1 Hz, 1H), 7.70–7.66 (m, 2H), 7.60–7.56 (m, 2H), 7.45 (d, J = 8.1, 1H), 7.19–7.13 (m, 2H), 7.12 (d, J = 8.1, 1H), 7.01 (d, J = 8.0 Hz, 1H), 6.89–6.85 (m, J = 7.2 Hz, 1H), 6.85–6.80 (m, 2H), 6.77–6.73 (m, 1H), 6.67–6.63 (m, 2H), 6.65 (s with satellites, J_{PtH} = 45.6 Hz, 1H), 6.34 (d with satellites, J_{PtH} = 22.0 Hz, J_{HH} = 5.8 Hz, 1H), 6.12 (d with satellites, J_{PtH} = 22.0 Hz, J_{HH} = 5.8 Hz, 1H), 5.81 (s with satellites, J = 54.7 Hz, 1H), 2.22 (s, 3H), 2.21 (s, 3H), 1.94 (s, 3H), 1.67 (s, 3H). ^{19}F NMR (377 MHz, CD_2Cl_2): δ –74.93 (s, 3F), –75.85 (br, 6F). Anal. Calcd for $C_{55}H_{42}F_9N_4O_6Pt_2$: C 46.19, H 3.02, N 3.99. Found: C 45.63, H 3.05, N 3.84.

[{Pt(tpy)$_2$(O$_2$CCF$_3$-κ*O*)}$_2$] (3'). This complex could not be obtained in pure form. The following NMR data were extracted from a mixture of **3** and **3'** in which the latter was the major component. ^{1}H NMR (401 MHz, CD_2Cl_2): δ 8.51 (s with satellites, J_{PtH} = 34.2 Hz, 2H), 7.56 (d, 8.0 Hz, 4H), 7.56 (d, 8.0 Hz, 4H), 7.28 (d, J = 8.3 Hz, 2H), 7.04 (d, J = 8.3 Hz, 2H), 6.96 (d, J = 8.0 Hz, 2H), 6.86–6.81 (m, 4H), 6.70 (d, J = 8.0 Hz, 2H), 6.68–6.63 (m, 4H), 6.63–6.56 (m, 2H), 6.10 (d, 2H), 2.09 (s, 6H), 2.06 (s, 6H). ^{19}F NMR (377 MHz, CD_2Cl_2): δ –75.6 (s, 6F).

General procedure for *mer*-[Pt(tpy)$_2$(OAc)(CCAr)] (*mer*-4). To a solution of *cis*-[Pt(tpy)$_2$] in CH_2Cl_2 (10 mL) was added PhI(OAc)$_2$ (0.285 mmol) and the mixture was stirred for 3 h. The alkyne ArCCH (0.65 mmol) and Na_2CO_3 (0.9 mmol) were then added and the suspension was stirred for 16 h and filtered through Celite. The solvent was evaporated under reduced pressure and the residue was treated with acetone (1 mL) and Et_2O (5 mL), whereupon a white precipitate formed, which was collected by filtration, washed with Et_2O (3 × 2 mL) and vacuum-dried to give the corresponding complex *mer*-**4**.

Data for *mer*-[Pt(tpy)$_2$(OAc)(CCC$_6$H$_4$OMe-*p*)] (*mer*-4a). White solid. Yield: 140 mg (65%) from complex *cis*-[Pt(tpy)$_2$] (160 mg). ^{1}H NMR (400 MHz, CD_2Cl_2): δ 10.06 (ddd, J = 5.6, 1.7, 0.8 Hz, 1H), 8.04 (ddd, J = 15.5, 8.4, 1.7 Hz, 1H), 7.97 (d, J = 7.9 Hz, 1H), 7.81 (d, J = 8.4 Hz, 1H), 7.78–7.68 (m, 2H), 7.64 (d, J = 7.9, 1H), 7.60 (d, J = 7.9 Hz, 1H), 7.48 (ddd, J = 7.2, 5.6, 1.5 Hz, 1H), 7.33 (d with satellites, J_{PtH} = 32.5 Hz, J_{HH} = 6.2 Hz, 1H), 7.25 (d with satellites, J_{PtH} = 49.3 Hz, J_{HH} = 7.9 Hz, 1H), 7.13–7.05 (m, 2H), 6.91–6.82 (m, 2H), 6.73–6.64 (m, 2H), 6.22 (s with satellites, J_{PtH} = 23.0 Hz, 1H), 3.72 (s, 3H), 2.60 (s, 3H), 2.05 (s, 3H), 1.91 (s, 3H). ^{13}C NMR (151 MHz, CD_2Cl_2): δ 174.9 (C), 165.8 (J_{PtC} = 52.4 Hz, C), 165.1 (J_{PtC} = 52.4 Hz, C), 158.3 (C), 153.3 (C), 152.7 (CH), 146.2 (CH), 145.2 (C), 143.3 (C), 142.2 (C), 141.2 (C), 140.0 (CH), 139.6 (CH), 137.9 (C), 133.8 (CH), 133.2 (CH), 129.1 (CH), 126.5 (CH), 126.4 (CH), 125.1 (CH), 125.0 (CH), 123.3 (CH), 122.9 (CH), 120.6 (CH), 119.9 (CH), 113.8 (CH), 102.0 (C), 99.6 (C), 55.7 (CH$_3$), 23.7 (J_{PtC} = 44.2 Hz, CH$_3$), 22.7 (CH$_3$), 22.0 (CH$_3$). Anal. Calcd for $C_{35}H_{30}N_2O_3Pt$: C 58.25, H 4.19, N 3.88. Found: C 58.14, H 4.15, N 3.80.

Data for *mer*-[Pt(tpy)$_2$(OAc)(CCPh)] (*mer*-4b). White solid. Yield: 220 mg (89%) from complex *cis*-[Pt(tpy)$_2$] (190 mg). ^{1}H NMR (300 MHz, CD_2Cl_2): δ 10.05 (d with satellites, J_{PtH} = 16.8 Hz, J_{HH} = 5.6 Hz, 1H), 8.08–7.94 (m, 2H), 7.86–7.79 (m, 1H), 7.79–7.68 (m, 2H), 7.65 (d, J = 8.0 Hz, 1H), 7.61 (d, J = 8.0 Hz, 1H), 7.48 (ddd, J = 7.2, 5.6, 1.6 Hz, 1H), 7.33 (d with satellites, J_{PtH} = 33.6 Hz, J_{HH} = 6.2 Hz, 1H), 7.25 (d, J = 7.9 Hz, 1H), 7.18–7.03 (m, 5H), 6.91–6.82 (m, 2H), 6.22 (s with satellites, J_{PtH} = 23.2 Hz, 1H), 2.60 (s, 3H), 2.05 (s, 3H), 1.91 (s, 3H). ^{13}C NMR (151 MHz, CD_2Cl_2): δ 174.94 (C), 165.9 (J_{PtC} = 50.6 Hz, C), 165.1 (J_{PtC} = 22.2 Hz, C), 153.1 (C), 152.8 (CH), 146.2 (J_{PtC} =72.6 Hz, CH), 145.2 (C), 143.3 (J_{PtC} = 33.2 Hz, C), 142.2 (C), 141.2 (J_{PtC} = 31.1 Hz, C), 140.0 (CH), 139.7 (CH), 137.9 (C), 133.8 (J_{PtC} = 26.3 Hz, CH), 132.1 (CH), , 129.1 (J_{PtC} = 159.6 Hz, CH), 128.3 (CH), 126.53 (CH), 126.47 (CH), 126.1, 125.1 (CH), 125.0 (CH), 123.3 (J_{PtC} = 139.4 Hz, CH), 122.9 (J_{PtC} = 30.8 Hz, CH), 120.6 (J_{PtC} = 36.1 Hz, CH), 120.0 (CH), 104.8 (C), 100.4 (C), 23.6 (J_{PtC} = 36.9 Hz, CH$_3$), 22.7 (CH$_3$), 22.0 (CH$_3$). Anal. Calcd for $C_{34}H_{28}N_2O_2Pt$: C 59.04, H 4.08, N 4.05. Found: C 58.96, H 4.10, N 4.00.

Data for *mer*-[Pt(tpy)$_2$(OAc)(CCC$_6$H$_4$CF$_3$-*p*)] *(mer*-4c). White solid. Yield: 180 mg (65%) from complex *cis*-[Pt(tpy)$_2$] (200 mg). ^{1}H NMR (300 MHz, CD_2Cl_2): δ 10.01 (d with satellites, J_{PtH} = 16.7 Hz, J_{HH} = 5.6 Hz, 1H), 8.11–7.93 (m, 2H), 7.87–7.79 (m, 1H), 7.79–7.69 (m, 1H), 7.65 (d, J = 12.6, 1H), 7.61 (d, J = 12.6, 1H) 7.39 (d, J = 8.2 Hz, 2H), 7.33 (d, J = 6.1 Hz, 1 H), 7.31–7.22 (m, 3H), 6.93–6.83 (m, 2H), 6.22 (s with satellites, J_{PtH} = 23.4 Hz, 1H), 2.59 (s, 3H), 2.05 (s, 3H), 1.91 (s, 3H). ^{13}C NMR (151 MHz, CD_2Cl_2): δ 174.9 (C), 165.8 (C, 50.5 Hz), 146.9 (C), 152.8 (CH), 152.7 (C), 146.2 (CH), 145.0 (C), 143.4 (C), 142.1 (C), 141.3 (C) ,140.1 (CH), 139.8 (CH), 137.9 (C), 133.7 (J_{PtC} = 28.1 Hz, CH), 132.3 (CH), 129.1 (CH), 127.4 (q, J_{CF} = 32.4 Hz, C), 126.7 (CH), 126.6 (CH), 125.2 (CH), 125.1 (CH), 125.1 (q, J_{CF} = 272.4 Hz, CF$_3$), 123.3 (CH), 123.0 (J_{PtC} = 26.8 Hz, CH), 120.6 (J_{PtC} = 34.8 Hz, CH), 120.0 (CH), 109.7 (J_{PtC} = 794.9 Hz, C), 99.8 (J_{PtC} = 146.7 Hz, C), 23.6 (J_{PtC} = 41.8 Hz, CH$_3$), 22.7 (CH$_3$), 22.0 (CH$_3$). ^{19}F NMR (282 MHz, CD_2Cl_2): δ –62.66 (s, 3F). Anal. Calcd for $C_{35}H_{27}N_2O_2F_3Pt\cdot 1/3CH_2Cl_2$: C 53.86, H 3.54, N 3.56. Found: C 53.58, H 3.67, N 3.40.

Data for *mer*-[Pt(tpy)$_2$(OAc)(CCC$_6$H$_3$F$_2$-3,5)] (*mer*-4d). White solid. Yield: 110 mg (73%) from complex *cis*-[Pt(tpy)$_2$] (110 mg). ^{1}H NMR (300 MHz, CD$_2$Cl$_2$): δ 9.96 (d with satellites, J_{PtH} = 17.4 Hz, J_{HH} = 5.6 Hz, 1H), 8.09–7.94 (m, 2H), 7.87–7.78 (m, 1H), 7.78–7.69 (m, 2H), 7.65 (d, J = 8.1 Hz, 1H), 7.61 (d, J = 8.2, 1H), 7.49 (ddd, J = 7.2, 5.6, 1.6 Hz, 1H), 7.32 (d with satellites, J_{PtH} = 32.4 Hz, J_{HH} = 6.0 Hz, 1H), 7.26 (d, J = 7.6 Hz, 1H), 6.94–6.82 (m, 2H), 6.76–6.62 (m, 2H), 6.56 (tt, J = 9.2, 2.4 Hz, 1H), 6.21 (s with satellites, J_{PtH} = 23.2 Hz, 1H), 2.59 (s, 3H), 2.06 (s, 3H), 1.91 (s, 3H). ^{13}C NMR (151 MHz, CD$_2$Cl$_2$): δ 174.9 (C), 165.9 (J_{PtC} = 58.5 Hz, C), 164.9 (C), 163.0 (dd, J_{CF} = 245.8 Hz, 14.2 Hz, 2C), 152.8 (CH), 152.6 (C), 146.2 (CH), 145.0 (C), 143.4 (C), 142.0 (C), 141.4 (J_{PtC} = 28.3 Hz, C), 140.2 (CH), 139.8 (CH), 137.9 (J_{PtC} = 28.3 Hz, C), 133.7 (J_{PtC} = 23.5 Hz, CH), 131.2–130.9 (m, C), 129.1 (CH), 126.7 (CH), 126.6 (CH), 125.13 (CH), 125.09 (CH), 123.3 (CH), 122.99 (J_{PtC} = 28.3 Hz, CH), 120.7 (J_{PtC} = 34.2 Hz, CH), 120.6 (CH), 114.1 (dd, J_{CF} = 20.2, 5.1 Hz, CH) 109.2 (C), 102.0 (t, J_{CF} = 25.9 Hz, CH), 99.0 (C), 23.5 (J_{PtC} = 46.3 Hz, CH$_3$), 22.7 (CH$_3$), 22.0 (CH$_3$). ^{19}F NMR (282 MHz, CD$_2$Cl$_2$): δ –112.42 (m, 2F). Anal. Calcd for C$_{34}$H$_{28}$N$_2$O$_2$F$_2$Pt: C 55.97, H 3.87, N 3.84. Found: C 56.01, H 4.11, N 3.76.

General procedure for *mer*-[Pt(tpy)$_2$(O$_2$CCF$_3$)(CCAr)] (*mer*-5). A solution of PhI(O$_2$CCF$_3$)$_2$ in Et$_2$O (5 mL) was added dropwise to a suspension of *cis*-[Pt(tpy)$_2$] (100 mg, 0.19 mmol) in Et$_2$O (5 mL). The mixture was stirred for 2 h and the solvent was evaporated under reduced pressure. The solid was dissolved in CH$_2$Cl$_2$ (10 mL) and the appropriate alkyne ArCCH (0.65 mmol) and Na$_2$CO$_3$ (0.9 mmol) were added. The suspension was stirred for 24 h at 40 °C and filtered through Celite. The filtrate was evaporated and the residue was dissolved in acetone (1 mL). The addition of Et$_2$O (5 mL) and pentane (3 mL) led to the precipitation of a white solid, which was collected by filtration, washed with Et$_2$O (3 × 2 mL) and vacuum-dried to give the corresponding complex *mer*-**5**.

Data for *mer*-[Pt(tpy)$_2$(O$_2$CCF$_3$)(CCC$_6$H$_4$OMe-*p*)] (*mer*-5a). White solid. Yield: 180 mg (63%) from complex *cis*-[Pt(tpy)$_2$] (200 mg). ^{1}H NMR (400 MHz, CD$_2$Cl$_2$): δ 10.11 (ddd, J = 5.6, 1.7, 0.8 Hz, 1H), 8.12 (ddd, J = 15.9, 8.1, 1.5 Hz, 1H), 8.06 (d, J = 8.1 Hz, 1H), 7.87 (d, J = 8.1 Hz, 1H), 7.80 (ddd, J = 15.5, 7.4, 1.5 Hz, 1H), 7.69 (t, J = 7.3 Hz, 3H), 7.57 (ddd, J = 7.1, 5.6, 1.5 Hz, 1H), 7.38 (d, J = 6.3 Hz, 1H), 7.31 (d, J = 7.7 Hz 1H), 7.19–7.11 (m, 2H), 6.99–6.87 (m, 2H), 6.77–6.69 (m, 2H), 6.28 (s with satellites, J_{PtH} = 21.7 Hz, 1H), 3.77 (s, 3H), 2.62 (s, 3H), 2.11 (s, 3H). ^{13}C NMR (151 MHz, CD$_2$Cl$_2$): δ 168.2 (C), 165.2 (C), 161.0 (q, J_{CF} = 35.8 Hz),158.5 (C), 152.5 (CH), 152.2 (C), 146.3 (CH), 145.1 (C), 143.8 (J_{PtC} = 31.2 Hz, C), 142.03 (C), 141.8 (J_{PtC} = 29.1 Hz, C), 140.6 (CH), 140.1 (CH), 137.7 (J_{PtC} = 36.9 Hz), 133.8 (J_{PtC} = 29.4 Hz), 133.3 (CH), 128.7 (CH), 127.0 (CH), 126.8 (CH), 125.2 (CH), 123.7 (CH), 123.1 (J_{PtC} = 31.2 Hz, CH), 120.8(J_{PtC} = 39.0 Hz, CH), 120.3 (CH) 120.2 (C), 113.91 (CH), 113.90 (q, J_{CF} = 296.5 Hz, CF$_3$) 100.7 (J_{PtC} = 794.2 Hz, C), 100.1 (J_{PtC} = 143.3 Hz, C), 55.7 (CH$_3$), 22.75 (CH$_3$), 22.03 (CH$_3$). ^{19}F NMR (377 MHz, CD$_2$Cl$_2$): δ –74.91 (s, J_{PtF} = 11.4 Hz, 3F). Anal. Calcd for C$_{34}$H$_{27}$N$_2$O$_3$F$_3$Pt: C 54.19, H 3.51, N 3.61. Found: C 54.29, H 3.77, N 3.51.

Data for *mer*-[Pt(tpy)$_2$(O$_2$CCF$_3$)(CCPh)] (*mer*-5b). White solid. Yield: 75 mg (53%) from complex *cis*-[Pt(tpy)$_2$] (100 mg). ^{1}H NMR (400 MHz, CD$_2$Cl$_2$): δ 10.07 (ddd, J = 5.6, 1.7, 0.8 Hz, 1H), 8.10 (ddd, J = 15.1, 8.1, 1.7 Hz, 1H), 8.02 (d, J = 8.2 Hz, 1H), 7.84 (d, J = 8.2 Hz, 1H) 7.76 (ddd, J = 8.3, 7.3, 1.5 Hz, 1H), 7.65 (t, J = 7.8 Hz, 3H), 7.53 (ddd, J = 7.2, 5.5, 1.5 Hz, 1H), 7.35 (d, J = 6.1 Hz, 1H), 7.28 (d, J = 8.5 Hz, 1H), 7.23–7.04 (m, 6H), 6.95–6.86 (m, 2H), 6.24 (s with satellites, J_{PtH} = 20.5 Hz, 1H), 2.58 (s, 3H), 2.07 (s, 3H). ^{19}F NMR (377 MHz, CD$_2$Cl$_2$): δ –74.91 (s, J_{PtF} = 11.3 Hz, 3F). Anal. Calcd for C$_{34}$H$_{25}$N$_2$O$_2$F$_3$Pt·1/4Et$_2$O: C 55.01, H 3.63, N 3.67. Found: C 55.65, H 3.67, N 3.79. Complex was too insoluble to obtain suitable APT data.

Data for *mer*-[Pt(tpy)$_2$(O$_2$CCF$_3$)(CCC$_6$H$_4$CF$_3$-*p*)] (*mer*-5c). White solid. Yield: 140 mg (46%) from complex *cis*-[Pt(tpy)$_2$] (200 mg). ^{1}H NMR (600 MHz, CD$_2$Cl$_2$): δ 10.01 (d, *J* = 5.7 Hz, 1H), 8.09 (ddd, *J* = 8.2, 7.4, 1.7 Hz, 1H), 8.03 (d, *J* = 8.2 Hz, 1H), 7.84 (d, *J* = 7.7 Hz, 1H), 7.77 (ddd, *J* = 8.2, 7.3, 1.4 Hz, 1H), 7.68–7.61 (m, 3H), 7.53 (ddd, *J* = 7.4, 5.6, 1.4 Hz, 1H), 7.43–7.38 (m, 2H), 7.35 (d with satellites, J_{PtH} = 36.9 Hz, J_{HH} = 6.6 Hz, 1H), 7.31–7.26 (m, 3H), 6.94–6.87 (m, 2H), 6.24 (s with satellites, *J* = 19.5 Hz, 1H), 2.58 (s, 3H), 2.07 (s, 3H). ^{13}C NMR (151 MHz, CD$_2$Cl$_2$): δ 166.2 (J_{PtC} = 62.9 Hz, C), 165.1 (J_{PtC} = 165.1 Hz, C), 161.0 (q, J_{PtC} = 37.1 Hz, C), 152.5 (CH), 151.5 (C), 146.4 (CH), 144.9 (C), 144.0 (J_{PtC} = 27.5 Hz, C), 142.0 (C), 142 (C), 140.7 (CH), 140.3 (CH), 137.6 (C), 133.8 (J_{PtC} = 27.6 Hz, CH), 132.4 (CH), 131.8 (C) 128.8 (CH), 127.8 (q, J_{CF} = 30.7 Hz, C), 127.2 (CH), 127.0 (CH), 125.4 (CH), 125.3 (CH), 125.24 (CH), 125.21 (CH), 125.1 (q, J_{CF} = 275.5 Hz, CF$_3$), 123.8 (CH), 123.2 (J_{PtC} = 32.8 Hz), 120.9 (J_{PtC} = 36.9 Hz), 120.5 (CH), 114.2 (q, J_{CF} = 291.2 Hz, CF$_3$), 108.1 (J_{PtC} = 788.6 Hz, C), 100.2 (J_{PtC} = 141.6 Hz, C), 22.8 (CH$_3$), 22.0 (CH$_3$).^{19}F NMR (282 MHz, CD$_2$Cl$_2$): δ –62.71 (s, 3F), –74.89 (s, J_{PtF} = 11.5 Hz, 3F). Anal. Calcd for $C_{35}H_{24}N_2O_2F_6Pt$: C 51.67, H 2.97, N 3.44. Found: C 51.63, H 3.03, N 3.52.

Data for *mer*-[Pt(tpy)$_2$(O$_2$CCF$_3$)(CCC$_6$H$_3$F$_2$-3,5)] (*mer*-5d). White solid. Yield: 170 mg (57%) from complex *cis*-[Pt(tpy)$_2$] (200 mg). ^{1}H NMR (300 MHz, CD$_2$Cl$_2$): δ 9.97 (d with satellites, J_{PtH} = 16.7 Hz, J_{HH} = 5.6 Hz, 1H), 8.15–7.99 (m, 2H), 7.88–7.73 (m, 2H), 7.69–7.60 (m, 3H), 7.54 (ddd, *J* = 7.1, 5.6, 1.5 Hz, 1H), 7.33 (d with satellites, J_{PtH} = 38.7 Hz, J_{HH} = 6.3 Hz, 1H), 7.28 (d, J_{HH} = 8.0 Hz, 1H), 6.97–6.85 (m, 2H), 6.76–6.65 (m, 2H), 6.58 (tt, *J* = 9.2, 2.4 Hz, 1H), 6.22 (s with satellites, J_{PtH} = 21.3 Hz, 1H), 2.58 (s, 3H), 2.07 (s, 3H). ^{13}C NMR (151 MHz, CD$_2$Cl$_2$): δ 166.2 (C), 165.0 (C), 163.0 (dd, J_{CF} = 246.3, 13.7 Hz, C), 161.1 (q, J_{CF} = 37.2 Hz, C), 152.5 (CH), 151.3 (C), 146.2 (CH), 145.0 (C), 143.9 (C), 142.0 (C), 141.8 (C), 140.7 (CH), 140.3 (CH), 137.6 (C), 133.7 (J_{PtC} = 25.7 Hz, CH), 130.6 (t, J_{CF} = 11.9 Hz, C), 129.1 (CH), 128.7 (CH), 127.1 (d, J_{CF} = 20.1 Hz, CH), 125.3 (d, J_{CF} = 7.3 Hz, CH), 123.8 (CH), 123.2 (J_{PtC} = 33.2 Hz, CH), 120.9 (J_{PtC} = 40.2 Hz, CH), 120.5 (CH), 114.9 (dd, J_{CF} = 20.6, 4.8 Hz, CH), 107.6 (J_{PtC} = 791.5 Hz, C), 102.2 (t, J_{CF} = 26.6 Hz, CH), 99.3 (J_{PtC} = 141.9 Hz, C), 22.7 (CH$_3$), 22.0 (CH$_3$). ^{19}F NMR (282 MHz, CD$_2$Cl$_2$): δ –74.90 (s, J_{PtF} = 11.6 Hz, 3F), –112.23 (m, 2F). Anal. Calcd for $C_{34}H_{23}N_2O_2F_5Pt{\cdot}2/3CH_2Cl_2$: C 49.85, H 3.06, N 3.25. Found: C 49.67, H 2.93, N 3.34.

General synthesis of *fac*-[Pt(tpy)$_2$(OOCCF$_3$)(CCAr)] (*fac*-5). A suspension of the appropriate complex *mer*-**5** in extra-dry CH$_3$CN (10 mL) was irradiated using a 365 nm LED source for 2 h. The solvent was evaporated under reduced pressure and a 1:5 mixture of CHCl$_3$ and Et$_2$O was added (12 mL), whereupon a pale yellow solid precipitated, which was collected by filtration, washed with Et$_2$O (3 × 2 mL) and vacuum-dried to give the corresponding complex *fac*-**5**.

Data for *fac*-[Pt(tpy)$_2$(OOCCF$_3$)(CCC$_6$H$_4$OMe-*p*)] (*fac*-5a). Pale yellow solid. Yield: 45 mg (45%) from complex *mer*-**5a** (100 mg). ^{1}H NMR (600 MHz, CD$_2$Cl$_2$): δ 9.09 (d, *J* = 5.3 Hz 1H), 8.35 (s, J_{PtH} = 43.4 Hz, 1H), 8.15–8.03 (m, 2H), 7.86 (d, *J* = 8.2 Hz, 1H), 7.79 (t, *J* = 7.9 Hz, 1H), 7.64 (d, *J* = 7.9 Hz, 1H), 7.60 (ddd, *J* = 7.2, 5.5, 1.5 Hz, 1H), 7.58–7.50 (m, 2H), 7.23 (d, *J* = 8.5 Hz, 1H), 7.12 (d, *J* = 9.8 Hz, 2H), 6.97 (t, *J* = 7.4 Hz), 6.92 (d, *J* = 7.2 Hz, 1H), 6.70 (d, *J* = 6.7 Hz, 1H), 6.24 (s, J_{PtH} = 51.2 Hz, 1H), 3.73 (s, 3H), 2.55 (s, 3H), 2.07 (s, 3H). ^{13}C NMR (151 MHz, CD$_2$Cl$_2$): δ 164.7 (J_{PtC} = 53.0 Hz, C), 161.4 (q, J_{CF} = 26.8 Hz, C), 161.3 (J_{PtC} = 44.0 Hz, C), 158.9 (C), 148.5 (CH), 147.3 (CH), 143.4 (J_{PtC} = 50.6 Hz, C), 143.07 (CH), 142.4 (J_{PtC} = 58.9 Hz, C), 140.8 (CH), 140.4 (CH), 139.6 (C), 138.2 (J_{PtC} = 2214.1 Hz, C), 135.6 (J_{PtC} = 50.6 Hz, CH), 133.5 (J_{PtC} = 51.2 Hz, CH), 128.4 (*J* = 833.1 Hz, C), 127.2 (CH), 127.1 (CH), 125.3 (J_{PtC} = 34.6 Hz, CH), 125.2 (J_{PtC} = 34.6 Hz, CH), 124.5 (CH), 123.0 (CH), 120.7 (CH), 120.1 (J_{PtC} = 21.1 Hz), 119.7 (C), 116.6 (q, J_{CF} = 291.2 Hz, C), 113.9 (CH), 92.5 (J_{PtC} = 276.1 Hz, C), 70.1 (J_{PtC} = 1377.1 Hz, C)

55.8 (CH_3), 22.5 (CH_3), 22.0 (CH_3). ^{19}F NMR (377 MHz, CD_2Cl_2): δ –75.28 (s, 3F). Anal. Calcd for $C_{34}H_{27}N_2O_3F_3Pt$: C 54.19, H 3.51, N 3.61. Found: C 54.21, H 3.60, N 3.52.

Data for *fac*-[Pt(tpy)$_2$(OOCCF$_3$)(CCPh)] (*fac*-5b). Pale yellow solid. Yield: 40 mg (53%) from complex *mer*-**5b** (75 mg). ^{1}H NMR (600 MHz, CD_2Cl_2): δ 9.10 (ddd, J = 5.5, 1.6, 0.8 Hz, 1H), 8.35 (s, J_{PtC} = 43.9 Hz, 1H), 8.12 (ddd, J = 15.7, 8.5, 1.6 Hz, 1H), 8.08 (d, J = 8.0 Hz, 1H), 7.87 (d, J = 8.1 Hz, 1H), 7.80 (ddd, J = 8.2, 7.4, 1.6 Hz, 1H), 7.65 (d, J = 7.9 Hz, 1H), 7.61 (ddd, J = 7.2, 5.5, 1.5 Hz, 1H), 7.57 (d, J = 8.4 Hz, 1H), 7.55 (d, J = 6.1 Hz, 1H), 7.24 (d, J = 8.0 Hz, 1H), 7.22–7.14 (m, 4H), 7.14–7.08 (m, 1H), 6.97 (ddd, J = 12.9, 6.5, 1.6 Hz, 1H), 6.92 (d, J = 8.0 Hz, 1H), 6.25 (s, J_{PtH} = 51.1 Hz, 1H), 2.55 (s, 3H), 2.07 (s, 3H). ^{13}C NMR (151 MHz, CD_2Cl_2): δ 164.7 (J_{PtC} = 45.96, C), 161.4 (q, J_{CF} = 23.2 Hz, C), 161.3 (J_{PtC} = 54.2 Hz, C), 148.5 (CH), 147.2 (CH), 143.5 (J_{PtC} = 50.7 Hz, C), 143.1 (C), 142.4 (J_{PtC} = 58.3 Hz, C), 140.9 (C), 140.4 (CH), 140.2 (CH), 139.6 (C), 138.2 (C), 135.6 (J_{PtC} = 50.7 Hz, CH), 133.6 (J_{PtC} = 50.7 Hz, CH), 132.2 (CH), 128.3 (CH), 127.4 (C), 127.2 (CH), 127.1 (CH), 126.8 (CH), 125.4 (CH), 125.2 (CH), 124.5 (CH), 123.1 (CH), 120.8 (CH), 120.2 (J = 19.4 Hz, CH), 120.2 (CH), 93.2 (J_{PtC} = 274.0 Hz, C), 72.6 (J_{PtC} = 1372.5 Hz, C), 30.3 (C), 22.5 (CH_3), 22.0 (CH_3). ^{19}F NMR (377 MHz, CD_2Cl_2): δ –74.77 (s, 3F). Anal. Calcd for $C_{34}H_{25}N_2O_2F_3Pt{\cdot}1/4Et_2O$: C 55.01, H 3.63, N 3.67. Found: C 55.55, H 3.56, N 3.77.

Data for *fac*-[Pt(tpy)$_2$(OOCCF$_3$)(CCC$_6$H$_4$CF$_3$-*p*)] (*fac*-5c). Pale yellow solid. Yield: 70 mg (58%) from complex *mer*-**5c** (120 mg). ^{1}H NMR (300 MHz, CD_2Cl_2): δ 9.08 (d with satellites, J_{PtH} = 13.7 Hz, J_{HH} = 5.6, Hz, 1H), 8.30 (s with satellites, J_{PtH} = 43.6 Hz, 1H), 8.17–8.05 (m, 2H), 7.91–7.76 (m, 2H), 7.69–7.47 (m, 5H), 7.46–7.37 (m, 2H), 7.34–7.27 (m, 2H), 7.24 (d, J = 7.8 Hz, 1H), 7.01–6.89 (m, 2H), 6.24 (s with satellites, J = 50.9 Hz, 1H), 2.55 (s, 3H), 2.07 (s, 3H). ^{13}C NMR (151 MHz, CD_2Cl_2): δ 164.7 (J_{PtC} = 54.9 Hz, C), 161.4 (J_{PtC} = 48 Hz, C), 148.5 (CH), 147.2 (CH), 143.6 (J_{PtC} = 49.9 Hz, C), 142.9 (C), 142.5 (C), 141.0 (CH), 140.5 (CH), 139.6 (C), 138.2 (C), 135.5 (J_{PtC} = 49.9 Hz, CH), 133.6 (J_{PtC} = 51.4 Hz), 132.5 (CH), 127.3 (d, J_{CF} = 9.3 Hz, CH), 125.6–125.1 (m), 124.6 (CH), 123.1 (J_{PtC} = 15.3 Hz, CH), 120.8 (J_{PtC} = 13.3 Hz, CH), 120.2 (J_{PtC} = 21.6 Hz, CH), 92.6 (J_{PtC} = 270.0 Hz, C), 77.1 (C), 22.5 (CH_3), 22.0 (CH_3). ^{19}F NMR (282 MHz, CD_2Cl_2): δ –62.80 (s, 3F), –75.28 (s, 3F). Anal. Calcd for $C_{35}H_{24}N_2O_2F_6Pt$: C 51.67, H 2.97, N 3.44. Found: C 51.78, H 3.15, N 3.20.

Data for *fac*-[Pt(tpy)$_2$(OOCCF$_3$)(CCC$_6$H$_3$F$_2$-3,5)] (*fac*-5d). Pale yellow solid. Yield: 50 mg (50%) from complex *mer*-**5d** (100 mg). ^{1}H NMR (600 MHz, CD_2Cl_2): δ 9.08 (d, J_{PtH} = 14.5 Hz, J_{HH} = 5.6, Hz, 1H), 8.26 (s, J_{PtH} = 43.2 Hz, 1H), 8.13 (ddd, J = 8.2, 7.4, 1.6 Hz, 1H), 8.09 (d, J = 8.3 Hz, 1H), 7.87 (d, J = 8.4 Hz, 1H), 7.83–7.78 (m, 1H), 7.65 (d, J = 8.0 Hz, 1H), 7.63–7.60 (m, 1H), 7.57 (d, J_{PtH} = 5.6 Hz, 1H), 7.53 (d, J_{PtH} = 20.7 Hz, J_{HH} = 5.8 Hz, 1H), 7.25 (d, J = 8 Hz, 1H), 7.00–6.96 (m, 1H), 6.93 (d, J = 8.5 Hz, 1H), 6.74–6.69 (m, 2H), 6.60 (tt, J = 9.2, 2.4 Hz, 1H), 6.24 (s, J_{PtH} = 51.1 Hz, 1H), 2.55 (s, 3H), 2.07 (s, 3H). ^{13}C NMR (151 MHz, CD_2Cl_2): δ 164.7 (J_{PtC} = 52.4 Hz, C), 163.0 (dd, J_{CF} = 246.0, 14.0 Hz, C), 161.3 (J_{PtC} = 49.8 Hz, C), 148.4 (CH), 147.2 (CH), 143.6 (J_{PtC} = 53.9 Hz, C), 142.9 (C), 142.6 (C), 141.0 (CH), 140.5 (CH), 139.5 (C), 138.2 (C), 135.4 (J_{PtC} = 48.1 Hz, CH), 133.5 (J_{PtC} = 45.1, CH), 130.2 (t, J_{CF} = 11.6 Hz, C), 128.2 (C), 127.32 (CH), 127.7 (CH), 125.7–125.1 (m), 124.6 (CH), 123.1 (CH), 120.8 (CH), 120.3 (J_{PtC} = 20.9 Hz, CH), 115.1 (dd, J_{CF} = 22.5, 5.0 Hz, CH), 102.8 (t, J_{CF} = 26.0 Hz, CH), 91.8 (J_{PtF} = 275.4 Hz, C), 76.7 (J_{PtC} = 1371.2 Hz, C), 22.5 (CH_3), 22.0 (CH_3). ^{19}F NMR (282 MHz, CD_2Cl_2): δ –75.30 (s, 3F), –112.02 (m, 2F). Anal. Calcd for $C_{34}H_{23}N_2O_2F_5Pt$: C 52.25, H 2.97, N 3.58. Found: C 52.28, H 3.09, N 3.55.

General synthesis of *fac*-[Pt(tpy)$_2$(Cl)(CCAr)] (*fac*-6). A suspension of complex *mer*-**5** in extra-dry CH_3CN (10 mL) in a flame-dried glassware was irradiated using 365 nm LED for 2 h. Solvent was evaporated under reduced pressure and a suspension of NH_4Cl in acetone (5 mL) was added. After stirring

for 4 h, solvent was evaporated, and the crude mixture was filtered over celite using CH_2Cl_2 and the crude mixture was passed through silica gel column hexane/AcOEt (1:1). A white solid was precipitated with CH_2Cl_2/Et_2O (1:10), collected by filtration washed with Et_2O and vacuum dried to give complex.

Data for *fac*-[Pt(tpy)$_2$(Cl)(CCC$_6$H$_4$OMe-*p*)] (*fac*-6a). White solid. Yield: 55 mg (62%) from complex *mer*-**5a** (100 mg). ^{1}H NMR (300 MHz, CD_2Cl_2): δ 9.72 (d with satellites, J_{PtH} = 15.8 Hz, J_{HH} = 5.5 Hz, 1H), 8.30 (s with satellites, J_{PtH} = 43.6 Hz, 1H), 8.15–8.00 (m, 2H), 7.88 (d, J = 8.2 Hz, 1H), 7.82–7.72 (m, 1H), 7.70–7.54 (m, 3H), 7.39 (d with satellites, J_{PtH} = 21.2 Hz, J_{HH} = 6.2 Hz, 1H), 7.20 (ddd, J = 8.0, 1.8, 0.7 Hz, 1H), 7.15–7.05 (m, 2H), 7.02–6.87 (m, 2H), 6.74–6.63 (m, 2H), 6.36 (s with satellites, J_{PtH} = 50.0 Hz, 1H), 3.72 (s, 3H), 2.54 (s, 3H), 2.08 (s, 3H). ^{13}C NMR (151 MHz, CD_2Cl_2): δ 164.0 (J_{PtC} = 52.6 Hz, C), 162.4 (J_{PtC} = 52.6 Hz, C), 158.6 (C), 143.7 (J_{PtC} = 53.7 Hz, C), 142.8 (C), 142.5 (J_{PtC} = 55.5 Hz, C), 140.5 (CH), 139.9 (CH), 138.8 (C), 138.6 Hz (C), 137.6 (C), 135.4 (J_{PtC} = 51.4 Hz, CH), 133.3 (CH), 133.2 (CH), 126.9 (CH), 126.7–125.1 (CH), 124.4 (CH), 123.4 (CH), 120.8–120.2 (m, CH), 120.1 (J_{PtC} = 27.8 Hz, C), 113.8 (CH), 92.6 (J_{PtC} = 272.4 Hz, C), 68.8 (C), 55.7 (CH_3), 22.5 (CH_3), 22.0 (CH_3). Anal. Calcd for $C_{33}H_{27}ClN_2OPt\cdot 1/4CH_2Cl_2$: C 55.18, H 3.73, N 3.88. Found: C 54.94, H 4.09, N 4.16.

Data for *fac*-[Pt(tpy)$_2$(Cl)(CCPh)] (*fac*-6b). White solid. Yield: 40 mg (55%) from complex *mer*-**5b** (85 mg). ^{1}H NMR (600 MHz, CD_2Cl_2): δ 9.74 (ddd with satellites, J_{PtH} = 15.1 Hz, J_{HH} = 5.5, 1.6, 0.9 Hz, 1H), 8.31 (s with satellites, J_{PtH} = 43.8 Hz, 1H), 8.10 (ddd, J = 8.2, 7.3, 1.6 Hz, 1H), 8.06 (d, J = 8.1 Hz, 1H), 7.89 (d, J = 8.3, Hz, 1H), 7.78 (ddd, J = 8.2, 7.4, 1.6 Hz, 1H), 7.67 (d, J = 7.9 Hz, 1H), 7.62 (s with satellites, J_{PtH} = 6.4 Hz, 1H) 7.61–7.58 (m, 1H), 7.41 (ddd with satellites, J_{PtH} = 21.4 Hz, J_{HH} = 5.8, 1.5, 0.8 Hz, 1H), 7.22 (ddd, J = 7.9, 1.8, 0.7 Hz, 1H), 7.18–7.13 (m, 4H), 7.11–7.08 (m, 1H), 6.99–6.95 (m, 1H), 6.93 (d, J = 8.4 Hz, 1H), 6.37 (s, J = 49.3 Hz, 1H), 2.55 (s, 3H), 2.08 (s, 3H). ^{13}C NMR (151 MHz, CD_2Cl_2): δ 163.5 (J_{PtC} = 52.2 Hz, C), 161.8 (J_{PtC} = 48.0 Hz, C), 148.1 (CH), 145.5 (CH), 143.2 (55.4 Hz, C), 142.2 (C), 142.0 (C), 140.0 (CH), 139.3 (CH), 138.2 (CH), 138.0 (CH), 137.0 (CH), 134.8 (J_{PtC} = 52.6 Hz, CH), 132.6 (J_{PtC} = 52.6 Hz, CH), 131.5 (CH), 127.7 (CH), 126.3 (CH), 126.1 (CH), 126.0 (CH), 124.9 (J_{PtC} = 34.4 Hz, 1H), 124.8 (J_{PtC} = 30.48 Hz, CH), 123.9 (CH), 122.8 (J_{PtC} = 14.6 Hz, CH), 120.1 (J_{PtC} = 20.3 Hz, CH), 119.9 (J_{PtC} = 12.4 Hz, CH), 92.8 (J_{PtC} = 272.2 Hz, C), 21.9 (CH_3), 21.4 (CH_3). Anal. Calcd for $C_{33}H_{25}N_2ClPt$: C 57.53, H 3.77, N 4.19. Found: C 57.53, H 3.91, N 4.09.

Data for *fac*-[Pt(tpy)$_2$(Cl)(CCC$_6$H$_4$CF$_3$-*p*)] (*fac*-6c). White solid. Yield: 15 mg (37%) from complex *mer*-**5c** (45 mg). ^{1}H NMR (600 MHz, CD_2Cl_2): δ 9.73 (d with satellites, J_{PtH} = 14.5 Hz, J_{HH} = 5.3 Hz, 1H), 8.27 (s with satellites J_{PtH} = 43.9 Hz, 1H), 8.14–8.09 (m, 1H), 8.09–8.05 (m, 1H), 7.90 (d, J_{HH} = 8.1 Hz, 1H), 7.82–7.77 (m, 1H), 7.67 (d, J = 8.0 Hz, 1H), 7.64–7.58 (m, 2H), 7.43–7.37 (m, 3H), 7.28 (d, J = 8.0 Hz, 1H), 7.22 (d, 8.0 Hz, 1H), 7.00–6.96 (m, 1H), 6.94 (d, J = 8.0 Hz, 1H), 6.72–6.66 (m, 2H), 6.58 (tt, J = 9.1, 2.4 Hz, 1H), 6.37 (s with satellites, J_{PtH} = 49.3 Hz, 1H), 2.55 (s, 3H), 2.09 (s, 3H). ^{13}C NMR (151 MHz, CD_2Cl_2): δ 164.0 (J_{PtC} = 51.1 Hz, C), 162.4 (J_{PtC} = 48.6 Hz, C), 148.6 (CH), 146.1 (CH), 143.9 (J_{PtC} = 51.2 Hz, C), 142.6 (J_{PtC} = 56.4 Hz, C), 138.8 (C), 138.6 (C), 137.5 (C), 135.2 (J_{PtC} = 49.8 Hz, CH), 133.2 (J_{PtC} = 51.2 Hz, CH), 132.4 (CH), 131.6 (J_{PtC} = 25.3 Hz, C), 127.9 (q, J_{CF} = 31.0 Hz), 127.0 (CH), 126.8 (CH), 125.5 (J_{PtC} = 33.8 Hz, CH), 125.46 (J_{PtC} = 29.8 Hz, CH), 125.2 (CH), 125.17 (CH), 125.0 (q, J_{CF} = 271.5 Hz, CF_3), 124.5 (CH), 123.5 (J_{PtC} = 15.3 Hz, CH), 120.8 (J_{PtC} = 22.0 Hz, CH), 120.6 (J_{PtC} = 12.2 Hz, CH), 92.8 (J_{PtC} = 273.9 Hz, C), 76.4 (J_{PtC} = 1354.3 Hz, C), 22.5 (CH_3), 22.0 (CH_3). ^{19}F NMR (282 MHz, CD_2Cl_2): δ –60.7 (s, 3F). Anal. Calcd for $C_{33}H_{24}N_2F_3ClPt$: C 53.85, H 3.29, N 3.81. Found: C 53.88, H 3.14, N 3.79.

Data for *fac*-[Pt(tpy)$_2$(Cl)(CCC$_6$H$_4$F$_2$-3,5)] (*fac*-6d). White solid. Yield: 21 mg (47%) from complex *mer*-**5d** (50 mg). ^{1}H NMR (600 MHz, CD_2Cl_2): δ 9.72 (ddd with satellites, J_{PtH} = 14.8 Hz, J_{HH} = 5.5, 1.6, 0.8 Hz,

1H), 8.21 (s with satellites J_{PtH} = 43.5 Hz, 1H), 8.14–8.09 (m, 1H), 8.09–8.05 (m, 1H), 7.91–7.87 (m, 1H), 7.82–7.77 (m, 1H), 7.67 (d, J = 8.0 Hz, 1H), 7.64–7.58 (m, 2H), 7.39 (ddd with satellites, J_{PtH} = 21.0 Hz, J_{PtH} = 5.8, 1.6, 0.7 Hz, 1H), 7.23 (d, J = 7.8 Hz, 1H), 7.00–6.96 (m, 1H), 6.94 (d, J = 7.8 Hz, 1H), 6.72–6.66 (m, 2H), 6.58 (tt, J = 9.1, 2.4 Hz, 1H), 6.36 (s with satellites, J_{PtH} = 49.6 Hz, 1H), 2.55 (s, 3H), 2.09 (s, 3H). ^{13}C NMR (151 MHz, CD_2Cl_2): δ 164.0 (J_{PtC} = 52.0 Hz, C), 163.0 (dd, J_{CF} = 244.3, 13.4 Hz, C), 162.4 (J_{PtC} = 44.0 Hz, C), 148.6 (CH), 146.1 (CH), 143.9 (J_{PtC} = 54.3 Hz), 142.7 (J_{PtC} = 54.3 Hz), 142.6 (C), 140.7 (CH), 140.0 (CH), 138.8 (CH), 138.6 (CH), 137.4 (CH), 135.2 (J_{PtC} = 47.6 Hz), 133.2 (J_{PtC} = 49.7 Hz), 130.5 (t, J_{CF} = 14.0 Hz, C), 127.1 (CH), 126.8 (CH), 126.9 (d, J_{CF} = 33.9 Hz, CH), 125.53 (J_{Pt}C = 36.0 Hz, CH), 125.47 (J_{PtC} = 28.8 Hz, CH), 124.6 (CH), 123.5 (J_{PtC} = 15.1 Hz, CH), 120.7 (J_{PtC} = 21.4 Hz, CH) 120.6 (J_{PtC} = 13.7 Hz, CH), 114.9 (dd, J_{CF} = 21.2, 5.4 Hz, CH), 102.5 (t, J_{CF} = 25.2 Hz, C), 92.1 (C), 76.0 (C), 30.3 (C), 22.5 (CH_3), 22.0 (CH_3). ^{19}F NMR (282 MHz, CD_2Cl_2): δ –112.2 (m, 2F). Anal. Calcd for $C_{32}H_{23}N_2F_2ClPt$: C 54.59, H 3.29, N 3.98. Found: C 54.45, H 3.33, N 3.84.

Irradiation of *mer*-4 with 365 nm light. A stirred solution of *mer*-**4** (0.12 mmol) in CH_3CN (5 mL) was irradiated for 2 h under an N_2 atmosphere, whereupon an orange solution was obtained. The solvent was evaporated under reduced pressure and the residue was stirred with a suspension of NH_4Cl (1 mmol) in acetone (20 mL) for 16 h. The solvent was removed, and the residue was treated with CH_2Cl_2 (10 mL). The solids were separated by filtration through Celite, and the filtrate was evaporated to dryness. The residue was then chromatographed on silica gel using a hexane/$CHCl_3$ mixture as the eluent, gradually increasing the proportions of $CHCl_3$ from 5 to 40%. The addition of Et_3N (1%) to the hexane fraction was necessary to avoid partial hydrolysis of compound **8** to 5-methyl-2-(2-pyridyl)phenol. The detected and isolated photoproducts are given in Scheme 2 (see main text).

General procedure for [PtCl(tpy)(L^1-Ar)] (7). A solution of the appropriate complex *mer*-**4** in CH_3CN (300 mL) was irradiated in the thermostated photoreactor at 25 °C for 1 h under an N_2 atmosphere. The solvent was evaporated under reduced pressure, a suspension of NH_4Cl (1 mmol) in acetone (20 mL) was added, and the mixture was stirred for 24 h. The solvent was removed, and the residue was suspended in CH_2Cl_2 (10 mL). The solids were removed by filtration through Celite, and the filtrate was evaporated to dryness. The residue was chromatographed on silica gel using a hexane/$CHCl_3$ mixture as the eluent, gradually increasing the proportion of $CHCl_3$ from 5 to 40%. The volatiles were removed, the residue was triturated with Et_2O, and the precipitate was collected by filtration, washed with Et_2O (3 × 2 mL) and vacuum-dried to give the corresponding complex **7**.

Data for [Pt(tpy)(Cl)(L^1-C_6H_4OMe-*p*)] (7a). Yellow solid. Yield: 20 mg (22%) from *mer*-**4a** (100 mg). ^{1}H NMR (400 MHz, CD_2Cl_2): δ 11.91 (d, J = 7.0, Hz, 1H), 9.47 (d, J = 5.7, Hz, 1H), 8.89 (d, 1H), 8.62 (d, J = 8.7 Hz, 1H), 8.09 (ddd, J = 8.6, 7.0, 1.5 Hz, 1H), 7.86 (dd, J = 8.4, 2.2 Hz, 1H), 7.79 (ddd, J = 8.1, 7.4, 1.7 Hz, 1H), 7.71–7.53 (m, 4H), 7.37 (s with satellites, J_{PtH} = 54.5 Hz, 1H), 7.32 (d, J = 7.8 Hz, 1H), 7.24–7.14 (m, 2H), 6.98 (dd, J = 8.5, 2.8 Hz, 1H), 6.80–6.71 (m, 2H), 6.39 (s, J = 71.1 Hz, 1H), 3.77 (s, 3H), 2.48 (s, 3H), 1.95 (s, 3H). Anal. Calcd for $C_{33}H_{27}N_2OClPt$: C 56.78, H 3.92, N 4.01. Found: C 56.43, H 3.62, N 4.08. ^{13}C NMR data could not be registered because of the low solubility of this complex.

Data for [Pt(tpy)(Cl)(L^1-Ph)] (7b). Yellow solid. Yield: 11 mg (17%) from *mer*-**4b** (65 mg). ^{1}H NMR (400 MHz, CD_2Cl_2): δ 11.89 (d, J = 7.0 Hz, 1 H), 9.46 (d, J = 6.5 Hz, 1 H), 8.88 (d, J = 8.9 Hz, 1 H), 8.61 (d, J = 8.7 Hz, 1 H), 8.11 (t br, J = 8.0 Hz, 1 H), 7.94 (d, J = 8.2 Hz, 1 H), 7.77 (t , J = 7.3 Hz, 1 H), 7.60 (t, J = 8.7 Hz, 2 H), 7.43 (t br, J = 8.2 Hz, 1 H), 7.34–7.11 (m, 8 H), 6.75 (d, J = 8.3 Hz, 1 H), 6.41 (s with satellites, J_{PtH} = 70.4 Hz, 1 H), 2.46 (s, 3 H), 1.96 (s, 3 H). Anal. Calcd for $C_{32}H_{25}N_2ClPt$: C 57.53, H 3.77, N 4.18.

Found: C 57.53, H 3.67, N 4.20. ^{13}C NMR data could not be registered because of the low solubility of this complex.

5-Methyl-2-(2-pyridyl)phenyl acetate (8). A solution of *mer*-**4c** or *mer*-**4d** in CH_3CN (300 mL) was irradiated using the thermostated photoreactor at 25 °C for 15 min. The solvent was evaporated under reduced pressure and the crude mixture was chromatographed on silica gel using a hexane/AcOEt mixture (9:1) as the eluent. Et_3N was added to the hexane fraction (1%) to avoid partial hydrolysis to 5-methyl-2-(2-pyridyl)phenol. Compound **8** was obtained as a yellow oil after evaporation of the solvents. Yield: 15 mg (48%) from *mer*-**4c** (100 mg); 10 mg (46%) from *mer*-**4d** (70 mg). ^{1}H NMR (401 MHz, $CDCl_3$): δ 8.69 (ddd, *J* = 4.9, 1.9, 1.0 Hz, 1H), 7.72 (dt, *J* = 7.5, 1.8 Hz, 1H), 7.61 (d, *J* = 7.8 Hz, 1H), 7.53 (td, *J* = 8.0, 1.1 Hz, 1H), 7.22 (ddd, *J* = 7.5, 4.9, 1.2 Hz, 1H), 7.19–7.15 (m, 1H), 6.98 (s, 1H), 2.42 (s, 3H), 2.19 (s, 3H). ^{13}C NMR (101 MHz, CD_2Cl_2): δ 169.7 (C), 156.0 (C), 149.6 (CH), 148.0 (C), 140.3 (C), 136.4 (CH), 130.7 (CH), 130.3 (CH), 127.4 (CH), 123.8 (CH), 123.6 (CH), 122.1 (CH), 21.3 (CH_3), 21.1 (CH_3). These data are consistent with those reported in the literature.[2]

General procedure for *(Z,E)*-[Pt(tpy)(Cl)(L^2-Ar)] [*(Z/E)*-9]. A solution of the appropriate complex *fac*-**5** in CH_3CN (300 mL) was irradiated in the thermostated photoreactor at 25 °C for 1 h under an N_2 atmosphere. The corresponding complex *(Z/E)*-**9** was isolated following the workup procedure described for complexes **7**.

***(Z/E)*-[PtCl(tpy)(L^2-Ph)] [*(Z/E)*-9b].** Orange solid. Yield: 10 mg (25%) from *fac*-**5b** (45 mg). ^{1}H NMR (600 MHz, CD_2Cl_2): δ 13.34 (dt, *J* = 6.7, 1.0 Hz, 1H, *Z*), 10.31 (s, 1H, *E*), 9.57 (ddd, *J* = 5.6, 1.7, 0.8 Hz, 1H, *E*), 9.54 (ddd, *J* = 5.6, 1.7, 0.8 Hz, 1H, *Z*), 8.18–8.12 (m, 3H, *Z*+*E*), 8.10 (d, *J* = 6.7 Hz, 1H, *E*), 7.98–7.93 (m, 2H, *Z*+*E*), 7.84–7.80 (m, 2H, *Z*+*E*), 7.73–7.69 (m, 2H, *Z*+*E*), 7.54–7.43 (m, 6H), 7.43–7.35 (m, 4H, *Z*+*E*), 7.34–7.27 (m, 4H, *Z*+*E*), 7.26–7.20 (m, 3H), 7.14 (s, 1H, *E*), 7.03 (s with satellites, J_{PtH} = 74.7 Hz, 1H, *Z*), 6.88–6.83 (m, 2H, *Z*+*E*), 6.18 (s, 1H, *Z*), 2.41 (s, 3H, *E*), 2.19 (s, 3H, *Z*), 2.10 (s, 3H, *E*), 2.05 (s, 3H, *Z*). Anal. Calcd for $C_{32}H_{25}N_2ClPt$: C 57.53, H 3.77, N 4.18. Found: C 57.34, H 4.31, N 4.08. ^{13}C NMR data could not be registered because of the low solubility of this complex.

***(Z/E)*-[PtCl(tpy)(L^2-$C_6H_4CF_3$-*p*)] [*(Z/E)*-9c].** Orange solid. Yield: 18 mg (28%) from *fac*-**5c** (70 mg). ^{1}H NMR (600 MHz, CD_2Cl_2): δ 13.31 (dt, *J* = 6.7 Hz, 1H, *Z*), 10.28 (s, 1H, *E*), 9.54 (d, *J* = 5.6 Hz, 1H, *E*), 9.52 (d, *J* = 5.4 Hz, 1H, *Z*), 8.21–8.15 (m, 3H, *Z*+*E*), 8.10 (d, *J* = 7.0 Hz, 1H, *E*), 8.01 (t, *J* = 7.7 Hz, 1H, *E*), 7.98–7.94 (m, 2H, *Z*+*E*), 8.0 Hz, 1H), 7.86–7.80 (m, 2H, *Z*+*E*), 7.74–7.69 (m, 4H, *Z*+*E*), 7.66–7.62 (m, 1H, *Z*+*E*), 7.53–7.40 (m, 6H, *Z*+*E*), 7.30 (d, *J* = 8.1 Hz, 1H, *Z*), 7.26–7.23 (m, 2H, *Z*+*E*), 7.14 (s, 1H, *E*), 7.08 (t, *J* = 7.1 Hz, 1H, *E*), 7.05 (s, J_{PtH} = 69.5 1H, *Z*), 6.87 (d, *J* = 7.6 Hz, 2H, *Z*+*E*), 6.11 (s, 1H, *Z*), 2.40 (s, 3H, *E*), 2.20 (s, 3H, *Z*), 2.11 (s, 3H, *E*), 2.07 (s, 3H, *Z*). ^{19}F NMR (282 MHz, CD_2Cl_2): δ –62.4 (s, 3F). Anal. Calcd for $C_{33}H_{24}N_2ClF_3Pt$: C 53.85, H 3.29, N 3.81. Found: C 53.49, H 3.62, N 3.51. ^{13}C NMR data could not be registered because of the low solubility of this complex.

***(Z/E)*-[PtCl(tpy)(L^2-$C_6H_3F_2$-3,5)] [*(Z/E)*-9d].** Orange solid. Yield: 10 mg (10%) from *fac*-**5d** (110 mg). ^{1}H NMR (600 MHz, CD_2Cl_2): δ 13.28 (d, *J* = 6.6 Hz, 1H, *Z*), 10.24 (s, 1H, *E*), 9.56 (d, *J* = 5.6 Hz, 1H, *E*), 9.53 (d, *J* = 5.6 Hz, 1H, *Z*), 8.20–8.14 (m, 3H, *Z*+*E*), 8.05–8.01 (m, 1H, *E*), 7.96 (d, *J* = 8.0 Hz, 2H, *Z*+*E*), 7.86–7.81 (m, 2H, *Z*+*E*), 7.72 (m, 2H, *Z*+*E*), 7.49–7.44 (m, 3H, *Z*+*E*), 7.40 (d, *J* = 8.2 Hz, 1H, *E*) 7.32 (d, *J* = 7.9 Hz, 1H, *Z*), 7.27–7.23 (m, *J* = 7.5 Hz, 3H, *Z*+*E*), 7.17 (d, *J* = 9.6 Hz, 1H, *Z*), 7.14 (dt, *J* = 7.1, 1.4 Hz, 1H, *E*), 7.06 (s, 1H, *E*), 6.97 (s, J_{PtH} = 73.4 Hz, 1H, *Z*), 6.95–6.90 (m, 2H, *Z*+*E*), 6.89–6.85 (m, 2H *Z*+*E*), 6.74 (tt, *J* = 9.2, 2.4 Hz, 1H, *Z*), 6.68 (tt, *J* = 9.2, 2.4 Hz, 1H, *E*), 6.35 (s, 1H, *Z*), 2.38 (s, 3H, *E*), 2.29 (s, 3H, *Z*), 2.10 (s, 3H, *E*), 2.06 (s, 3H, *Z*). ^{19}F NMR (282 MHz, CD_2Cl_2): δ –110.1 (q, J_{FH} = 8.15 Hz, F), –110.8 (q, J_{FH}

= 8.2 Hz, F). Anal. Calcd for $C_{32}H_{23}N_2ClF_2Pt\cdot1.5CH_2Cl_2$: C 48.39, H 3.15, N 3.37. Found: C 48.33, H 3.36, N 3.28. ^{13}C NMR data could not be registered because of the low solubility of this complex.

1.5. X-ray structure determinations

Single crystals suitable for X-ray diffraction were obtained by slow liquid-liquid diffusion from CH_2Cl_2/hexane [**2**·CH_2Cl_2, **3**·CH_2Cl_2, *mer*-**4b**, *mer*-**5b**, *fac*-**5b**·CH_2Cl_2 and *(Z)*-**9b**)] or CH_2Cl_2/Et_2O (**3'** and **7b**·$1.5CH_2Cl_2$). The data were collected on a Bruker D8 QUEST diffractometer with monochromated Mo-$K\alpha$ radiation performing φ and ω scans. The structures were solved by direct methods and refined anisotropically on F^2 using the program SHELXL-2018 (G. M. Sheldrick, University of Göttingen).[3,4] Numerical details are given in Tables S1 and S2. *Special features of refinement:* In *mer*-**5b**, the trifluoroacetato ligand is disordered over two positions, *ca.* 55:45%. In *fac*-**5b**, the trifluoroacetato ligand and the solvent are disordered over two positions, *ca.* 66:34% or 55:45%, respectively. In **3**·CH_2Cl_2, the CF_3 group of the bridging trifluoroacetato ligand is disordered over two positions, *ca.* 86:14%; there are two independent non-coordinated trifluoroacetates in the asymmetric unit, which are disordered over two positions, *ca.* 65:35% and 77:23%; each of them forms a single hydrogen bond with a symmetry-related one, based on the O···O distances (mean values of 2.440 or 2.3745 Å for each of the pairs), which demonstrate the presence of $[(CF_3CO_2)_2H]^-$ as the counterion, but the hydrogen atom could not be located.

Table S1. Crystallographic data for **2**, **3**·CH_2Cl_2, **3'** and *mer*-**4b**.

	**2**·CH_2Cl_2	**3**·CH_2Cl_2	**3'**	*mer*-**4b**
formula	$C_{29}H_{22}Cl_2F_6N_2O_4Pt$	$C_{55}H_{43}Cl_2F_9N_4O_6Pt_2$	$C_{52}H_{40}F_6N_4O_4Pt_2$	$C_{34}H_{28}N_2O_2Pt$
fw	842.47	1489.02	1289.06	691.67
T (K)	100(2)	100(2)	100(2)	100(2)
λ (Å)	0.71073	0.71073	0.71073	0.71073
cryst syst	Triclinic	Triclinic	Monoclinic	Orthorhombic
space group	P-1	P-1	C2/c	Pbca
a (Å)	9.8618(8)	10.2901(4)	18.0178(8) Å	10.5686(6)
b (Å)	12.1072(10)	12.6876(5)	15.9403(8) Å	21.7168(13)
c (Å)	13.8521(15)	20.6172(7)	15.5714(7) Å	24.5292(15)
α (°)	94.306(3)	82.9780(10)	90	90
β (°)	110.285(2)	84.745(3)	91.804(2)	90
γ (°)	104.250(3)	78.7830(10)	90	90
V (Å^3)	1480.0(4)	2614.13(17)	4470.0(4)	5629.8(6)
Z	2	2	4	8
ρ_{calcd} (Mg m^{-3})	1.890	1.890	1.915	1.632
μ (mm^{-1})	4.997	5.535	6.330	5.018
R1^a	0.0200	0.0187	0.0.0190	0.0215
wR2^b	0.0481	0.0420	0.0397	0.0405

^{a}R1 = $\Sigma||F_o|-|F_c||/\Sigma|F_o|$ for reflections with $I > 2\sigma(I)$. ^{b}wR2 = $[\Sigma[w(F_o^2-F_c^2)^2/\Sigma[w(F_o^2)^2]]^{0.5}$ for all reflections; $w^{-1} = \sigma^2(F^2) + (aP)^2 + bP$, where $P = (2F_c^2 + F_o^2)/3$ and a and b are constants set by the program.

Table S2. Crystallographic data for *mer*-**5b**, *fac*-**5b**, **7b**·1.5CH_2Cl_2, and *(Z)*-**9b**.

	mer-**5b**	*fac*-**5b**·CH_2Cl_2	**7b**·1.5CH_2Cl_2	*(Z)*-**9b**
formula	$C_{34}H_{25}F_3N_2O_2Pt$	$C_{35}H_{27}Cl_2N_2O_2Pt$	$C_{33.5}H_{28}FCl_4N_2Pt$	$C_{32}H_{25}ClN_2Pt$
fw	745.65	830.57	795.47	668.08
T (K)	100(2)	100(2)	100(2)	100(2)
λ (Å)	0.71073	0.71073	0.71073	0.71073
cryst syst	Orthorhombic	Monoclinic	Monoclinic	Monoclinic
space group	Pbca	$P2_1/n$	$P2_{1/n}$	$P2_1/c$
a (Å)	10.7952(10)	12.8058(10)	12.0765(4)	15.7732(11)
b (Å)	21.422(9)	16.3447(13)	34.1851(10)	8.8124(6)
c (Å)	24.577(2)	15.9004(12)	15.6505(4)	18.1890(13)
α (°)	90	90	90	90
β (°)	90	103.028(10)	100.7290(3)	99.812(2)°
γ (°)	90	90	90	90
V (Å^3)	5683.5(15)	3242.4(4)	6348.1(3)	2491.3(3)
Z	8	4	8	4
ρ_{calcd} (Mg m^{-3})	1.743	1.701	1.665	1.781
μ (mm^{-1})	4.992	4.544	4.783	5.764
R1[a]	0.0234	0.0180	0.0345	0.0205
wR2[b]	0.0525	0.0393	0.0813	0.0472

[a] $R1 = \Sigma||F_o|-|F_c||/\Sigma|F_o|$ for reflections with $I > 2\sigma(I)$. [b] $wR2 = [\Sigma[w(F_o^2-F_c^2)^2/\Sigma[w(F_o^2)^2]]^{0.5}$ for all reflections; $w^{-1} = \sigma^2(F^2) + (aP)^2 + bP$, where $P = (2F_c^2 + F_o^2)/3$ and a and b are constants set by the program.

2. Crystal structures of 3, 3' and *mer*-4b

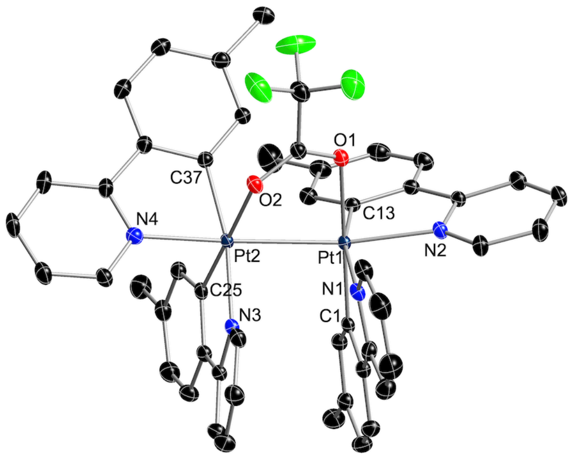

Figure S1. Structure of the cation of complex **3** (thermal ellipsoids at 50% probability). Hydrogen atoms and the $[(CF_3CO_2)_2H]^-$ anion are omitted.

Table S3. Selected bond distances (Å) and angles (°) for **3**.

Pt(1)-C(1)	1.995(2)	Pt(2)-C(25)	1.992(2)
Pt(1)-C(13)	2.026(2)	Pt(2)-C(37)	2.036(2)
Pt(1)-N(1)	2.1421(18)	Pt(2)-N(3)	2.1249(18)
Pt(1)-N(2)	2.1702(17)	Pt(2)-N(4)	2.1439(17)
Pt(1)-O(1)	2.1871(17)	Pt(2)-O(2)	2.1991(15)
Pt(1)-Pt(2)	2.66036(12)		
C(1)-Pt(1)-C(13)	91.63(8)	C(25)-Pt(2)-C(37)	97.57(8)
C(1)-Pt(1)-N(1)	81.26(8)	C(25)-Pt(2)-N(3)	80.22(8)
C(13)-Pt(1)-N(1)	165.41(7)	C(37)-Pt(2)-N(3)	169.22(7)
C(1)-Pt(1)-N(2)	93.46(7)	C(25)-Pt(2)-N(4)	87.18(7)
C(13)-Pt(1)-N(2)	80.01(8)	C(37)-Pt(2)-N(4)	79.92(7)
N(1)-Pt(1)-N(2)	87.67(7)	N(3)-Pt(2)-N(4)	89.41(7)
C(1)-Pt(1)-O(1)	173.04(7)	C(25)-Pt(2)-O(2)	179.18(7)
C(13)-Pt(1)-O(1)	95.25(7)	C(37)-Pt(2)-O(2)	81.62(7)
N(1)-Pt(1)-O(1)	91.79(7)	N(3)-Pt(2)-O(2)	100.56(6)
N(2)-Pt(1)-O(1)	86.69(6)	N(4)-Pt(2)-O(2)	92.55(6)
C(1)-Pt(1)-Pt(2)	96.78(6)	C(25)-Pt(2)-Pt(1)	98.80(6)
C(13)-Pt(1)-Pt(2)	103.13(6)	C(37)-Pt(2)-Pt(1)	100.63(6)
N(1)-Pt(1)-Pt(2)	90.42(5)	N(3)-Pt(2)-Pt(1)	90.14(5)
N(2)-Pt(1)-Pt(2)	169.18(5)	N(4)-Pt(2)-Pt(1)	173.83(5)
O(1)-Pt(1)-Pt(2)	82.73(4)	O(2)-Pt(2)-Pt(1)	81.49(4)

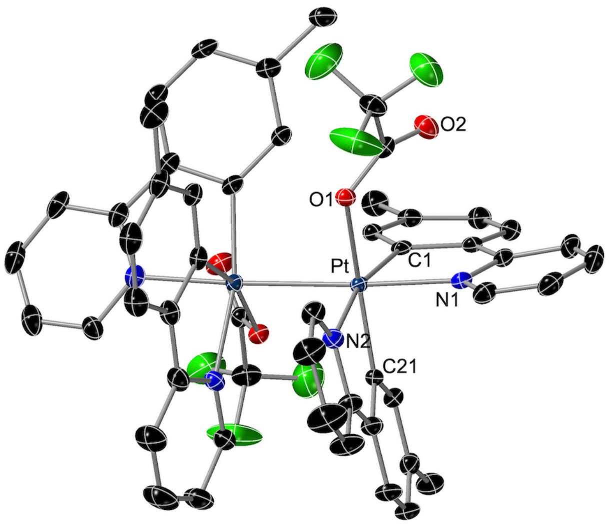

Figure S2. Structure of complex **3'** (thermal ellipsoids at 50% probability). Hydrogen atoms are omitted.

Table S4. Selected bond distances (Å) and angles (°) for **3'**.

Pt(1)-C(21)	1.997(3)	Pt(1)-C(1)	2.032(3)
Pt(1)-N(2)	2.135(2)	Pt(1)-N(1)	2.149(2)
Pt(1)-O(1)	2.1571(18)	Pt(1)-Pt(1)#1	2.6412(2)
O(1)-C(41)	1.267(3)		
C(21)-Pt(1)-C(1)	89.58(11)	C(21)-Pt(1)-N(2)	81.16(10)
C(1)-Pt(1)-N(2)	166.99(10)	C(21)-Pt(1)-N(1)	89.29(10)
C(1)-Pt(1)-N(1)	80.03(11)	N(2)-Pt(1)-N(1)	90.67(9)
C(21)-Pt(1)-O(1)	164.73(10)	C(1)-Pt(1)-O(1)	103.54(9)
N(2)-Pt(1)-O(1)	84.58(8)	N(1)-Pt(1)-O(1)	85.41(8)

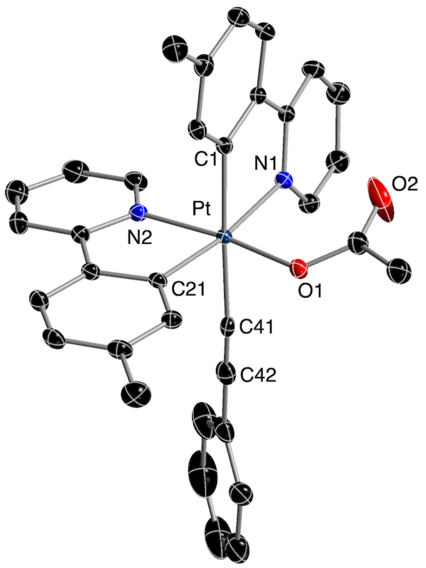

Figure S3. Structure of complex *mer*-**4b** (thermal ellipsoids at 50% probability). Hydrogen atoms are omitted.

Table S5. Selected bond distances (Å) and angles (°) for *mer*-**4b**.

Pt-C(21)	2.004(2)	Pt-N(1)	2.1357(17)
Pt-O(1)	2.0342(15)	O(1)-C(49)	1.291(3)
Pt-N(2)	2.0350(18)	O(2)-C(49)	1.201(3)
Pt-C(1)	2.049(2)	C(41)-C(42)	1.146(3)
Pt-C(41)	2.107(2)	C(42)-C(43)	1.448(3)
C(1)-Pt-N(1)	79.81(8)	C(42)-C(41)-Pt	174.4(2)
C(21)-Pt-N(2)	81.46(8)	C(41)-C(42)-C(43)	178.1(3)
O(1)-Pt-C(41)	91.62(7)	O(2)-C(49)-O(1)	126.3(2)
C(49)-O(1)-Pt	125.83(15)		

3. NMR spectra of new compounds

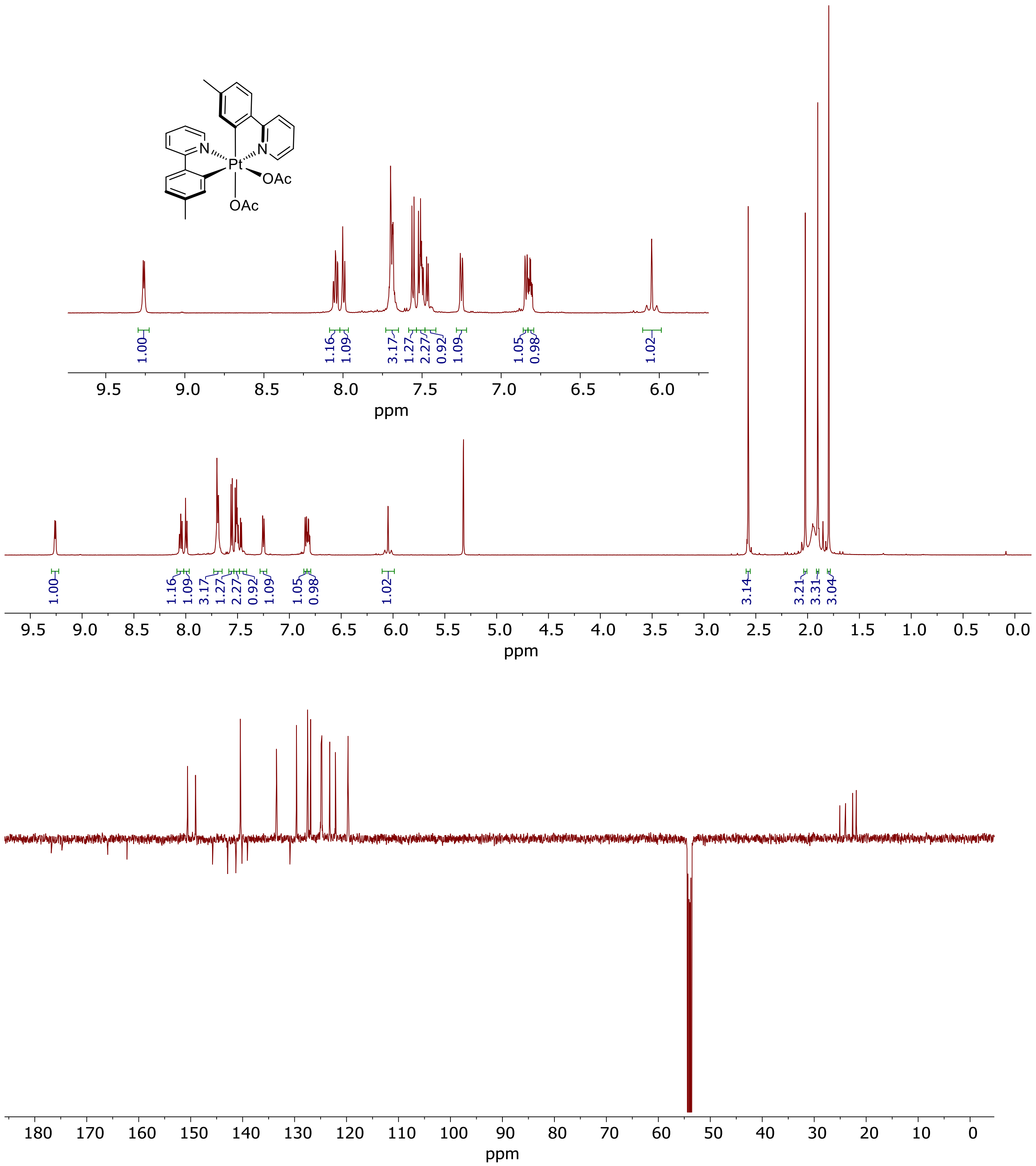

Figure S4. ^{1}H (top) and $^{13}C\{^{1}H\}$ APT (bottom) NMR spectra of complex **1** (CD_2Cl_2, 600 and 151 MHz, respectively).

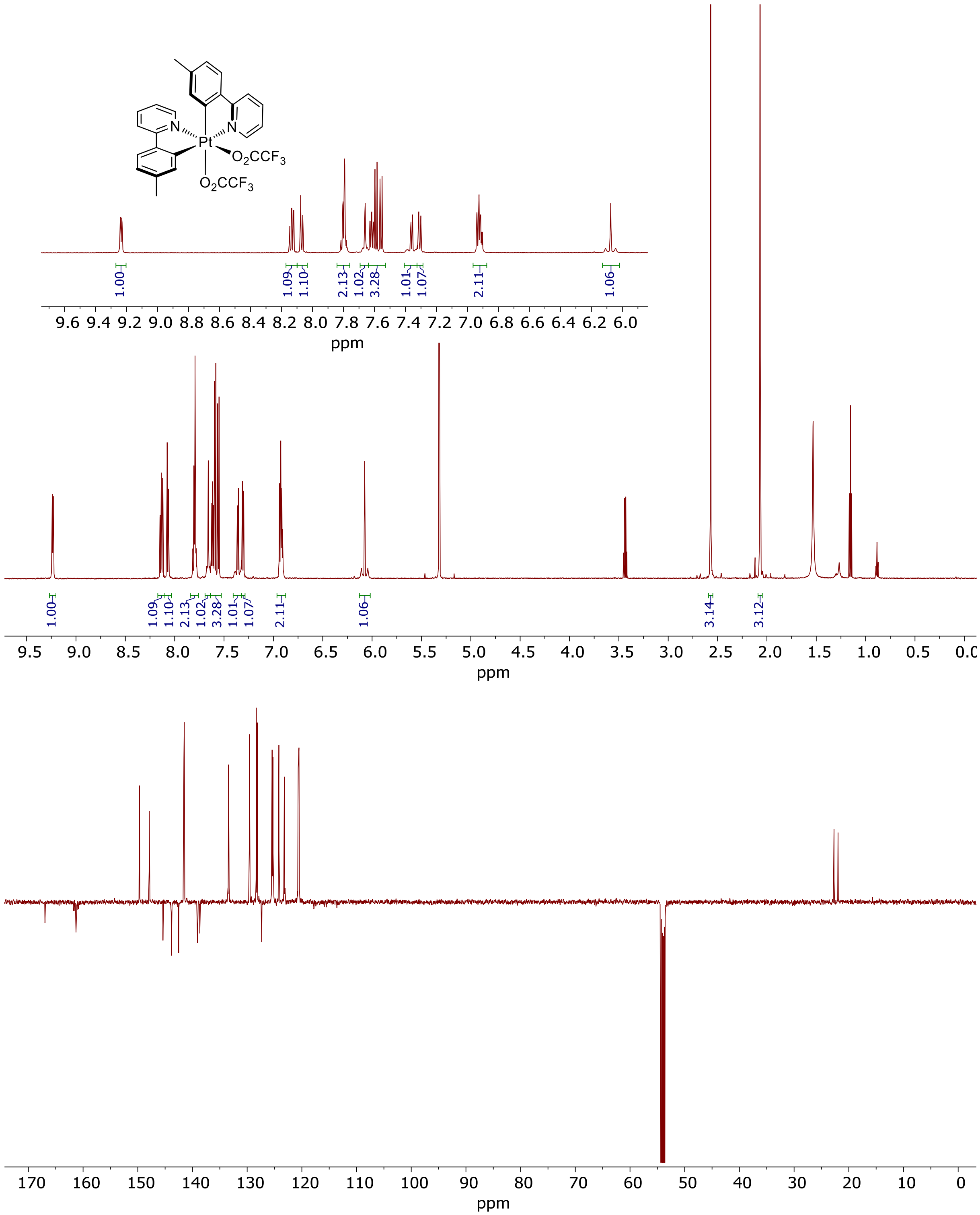

Figure S5. ^{1}H (top) and ^{13}C{^{1}H} APT (bottom) NMR spectra of complex **2** (CD_2Cl_2, 600 and 151 MHz, respectively).

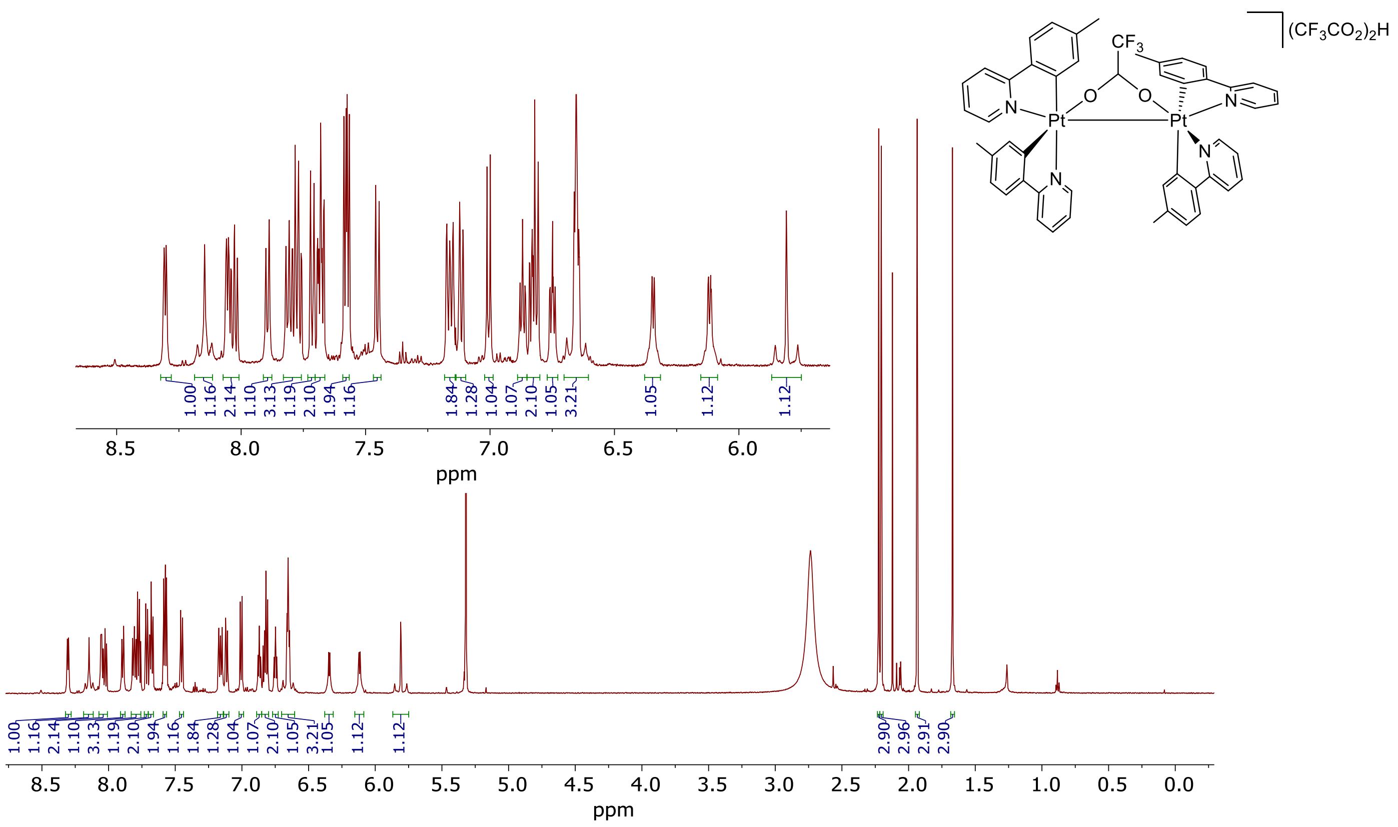

Figure S6. ^{1}H NMR spectrum of the crude product of the reaction between *cis*-[Pt(tpy)$_2$] and PhI(O_2CCF_3)$_2$ at –90 °C (complex **3**) (CD_2Cl_2, 600 MHz).

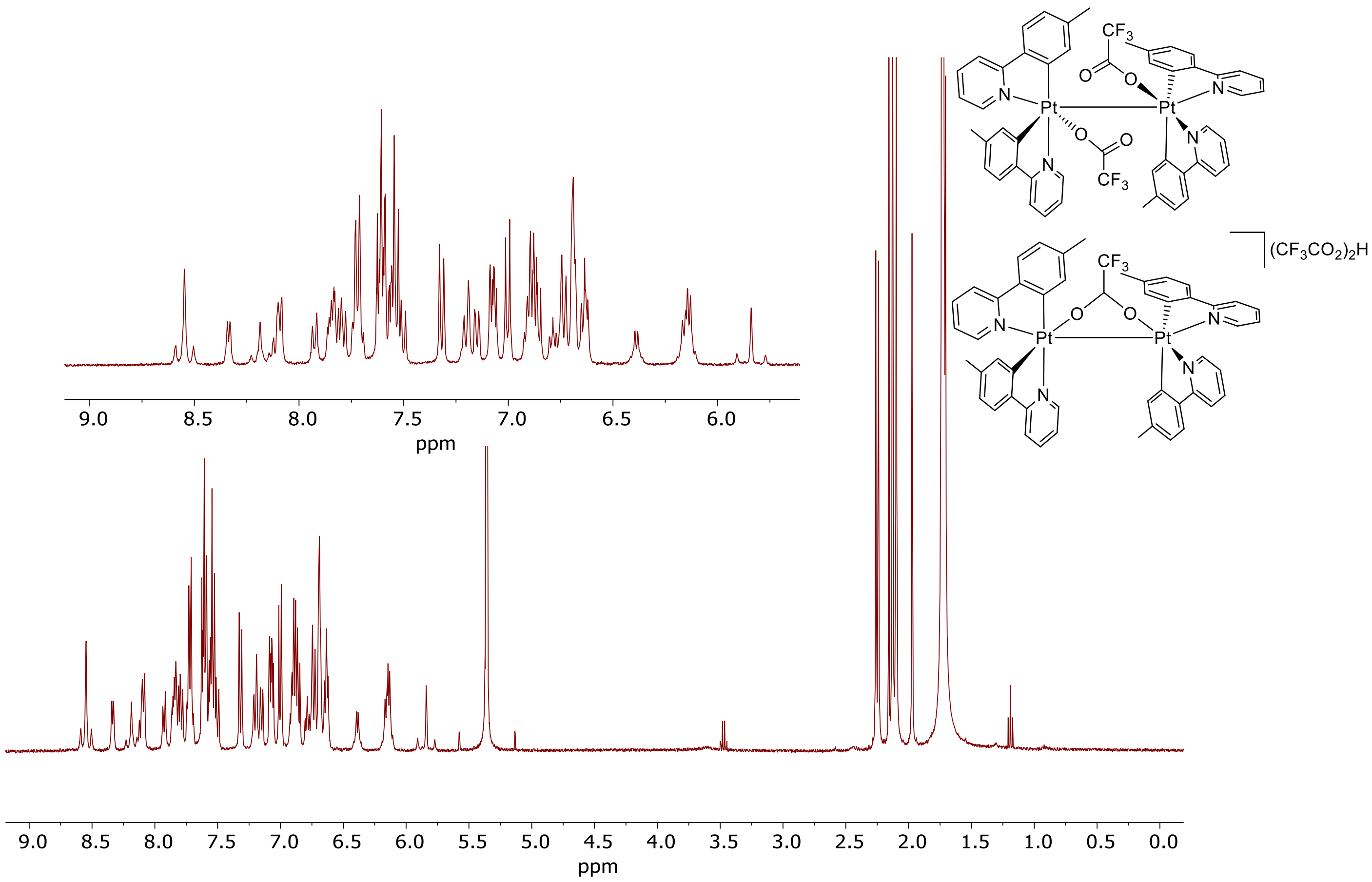

Figure S7. ^{1}H NMR spectrum of a mixture of complexes **3** and **3'** obtained by dissolving **3** in CD_2Cl_2 (600 MHz).

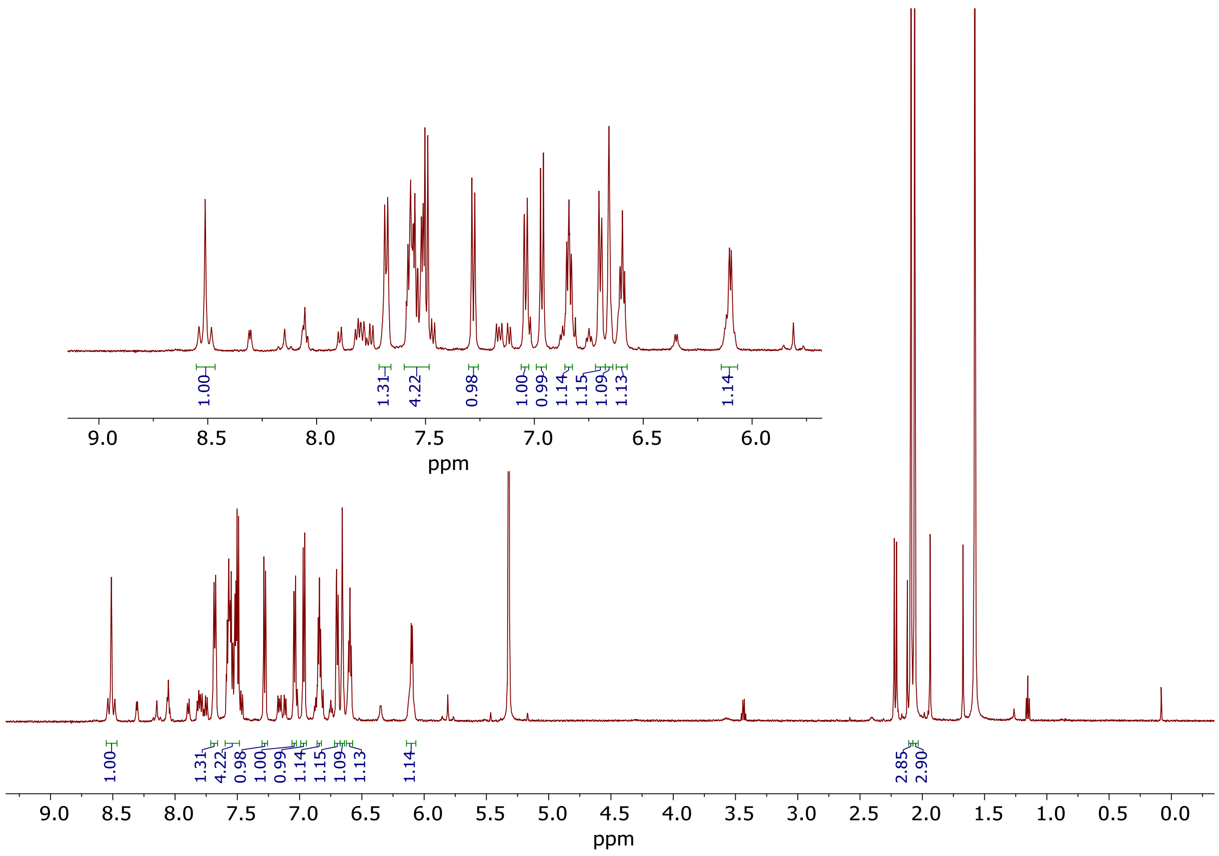

Figure S8. ^{1}H NMR spectrum of a mixture of complexes **3** and **3'**, in which **3'** is the major component (CD_2Cl_2, 600 MHz).

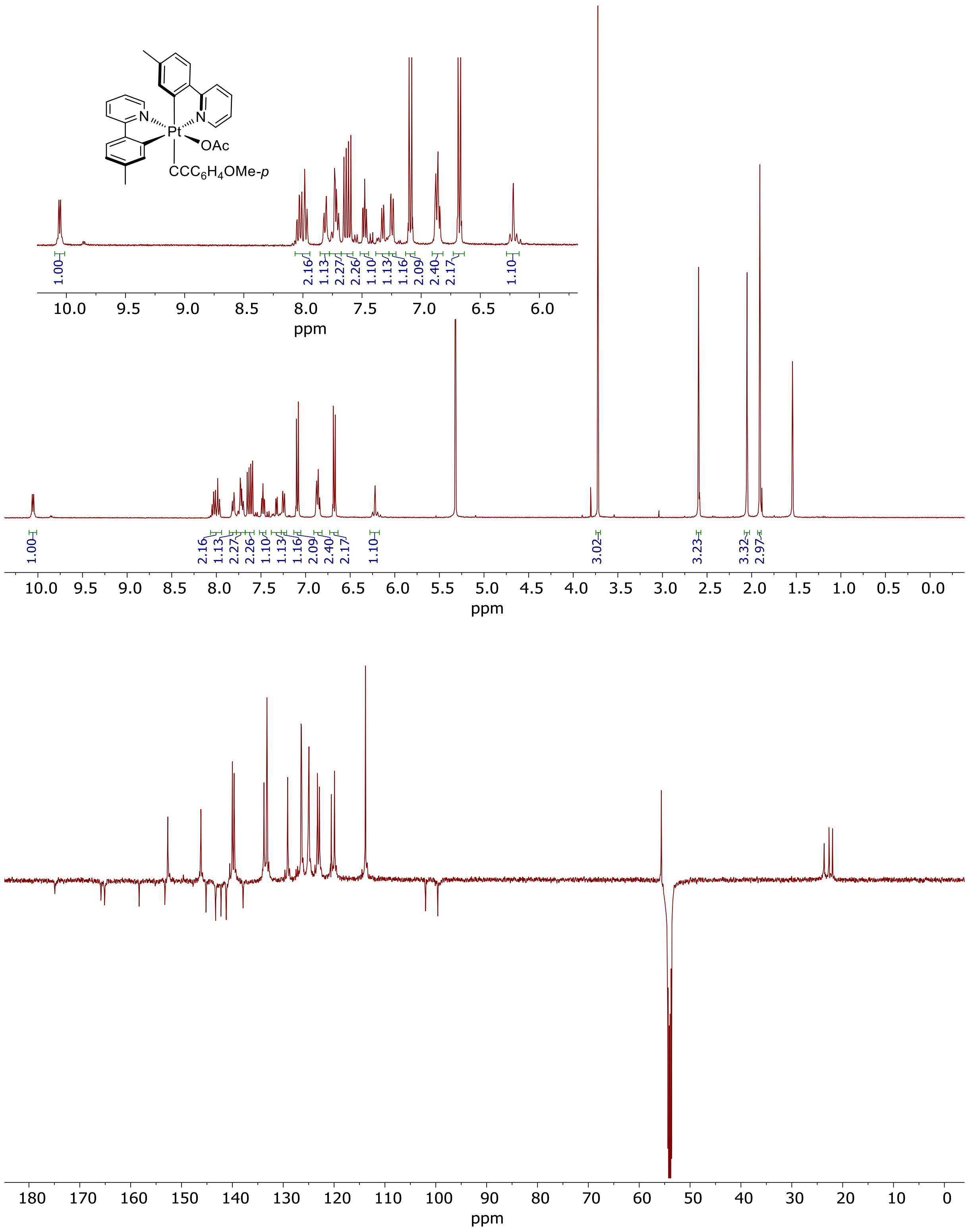

Figure S9. ^{1}H (top) and $^{13}C\{^{1}H\}$ APT (bottom) NMR spectra of complex *mer*-**4a** (CD_2Cl_2, 400 and 151 MHz, respectively).

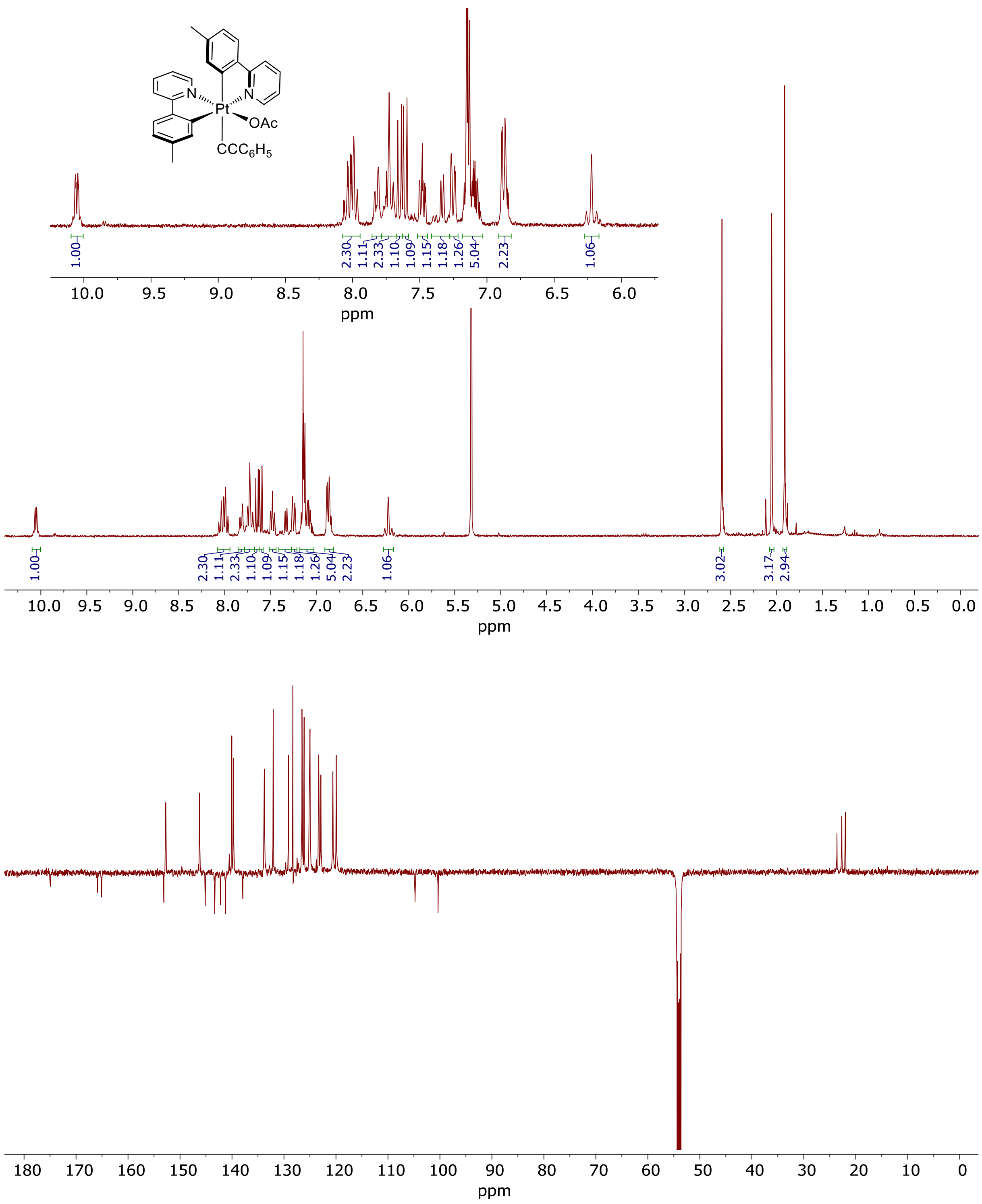

Figure S10. ^{1}H (top) and $^{13}C\{^{1}H\}$ APT (bottom) NMR spectra of complex *mer*-**4b** (CD_2Cl_2, 300 and 151 MHz, respectively).

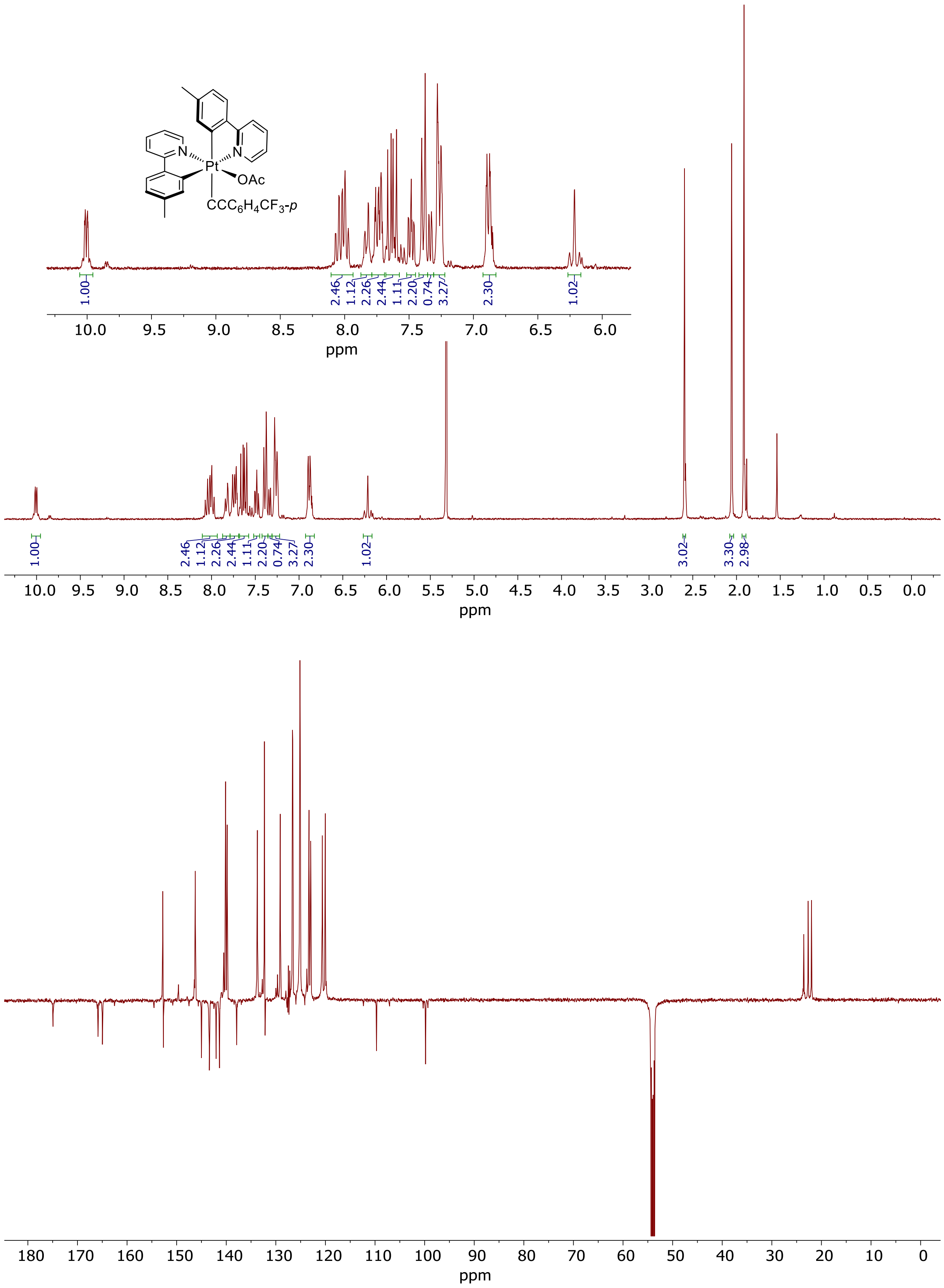

Figure S11. ^{1}H (top) and $^{13}C\{^{1}H\}$ APT (bottom) NMR spectra of complex *mer*-**4c** (CD_2Cl_2, 300 and 151 MHz, respectively).

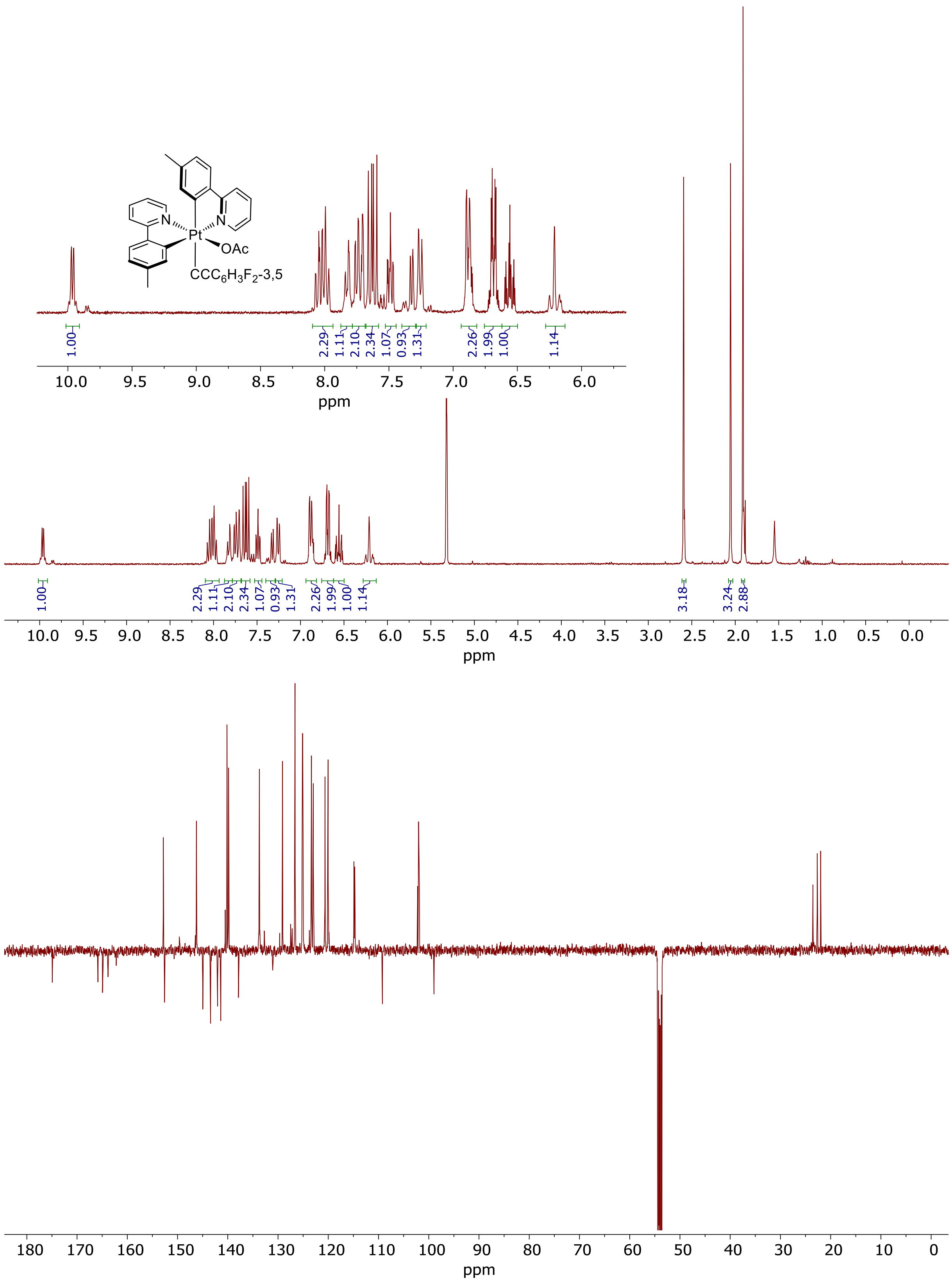

Figure S12. ^{1}H (top) and $^{13}C\{^{1}H\}$ APT (bottom) NMR spectra of complex *mer*-**4d** (CD_2Cl_2, 300 and 151 MHz, respectively.

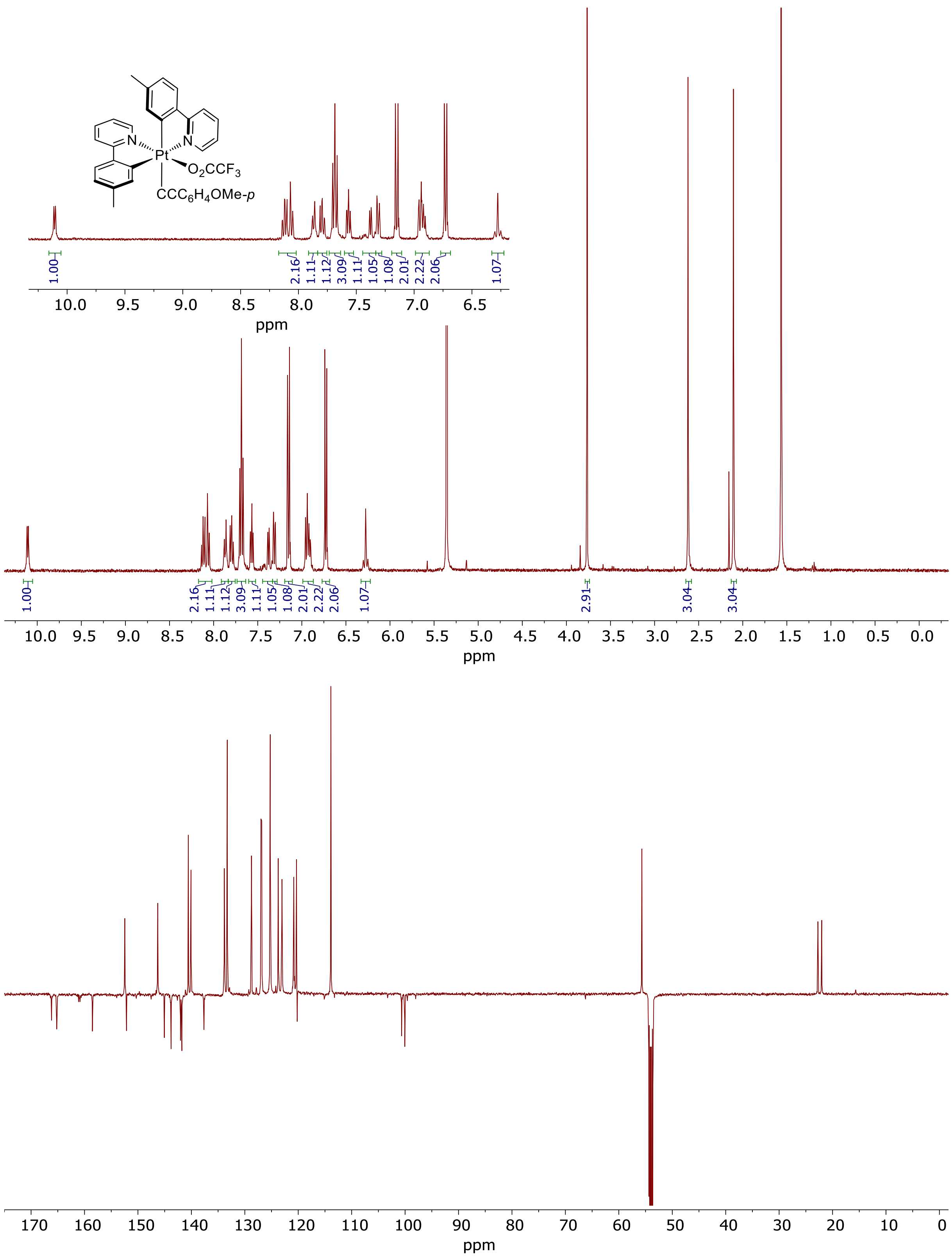

Figure S13. ^{1}H (top) and $^{13}C\{^{1}H\}$ APT (bottom) NMR spectra of complex *mer*-**5a** (CD_2Cl_2, 400 and 151 MHz, respectively).

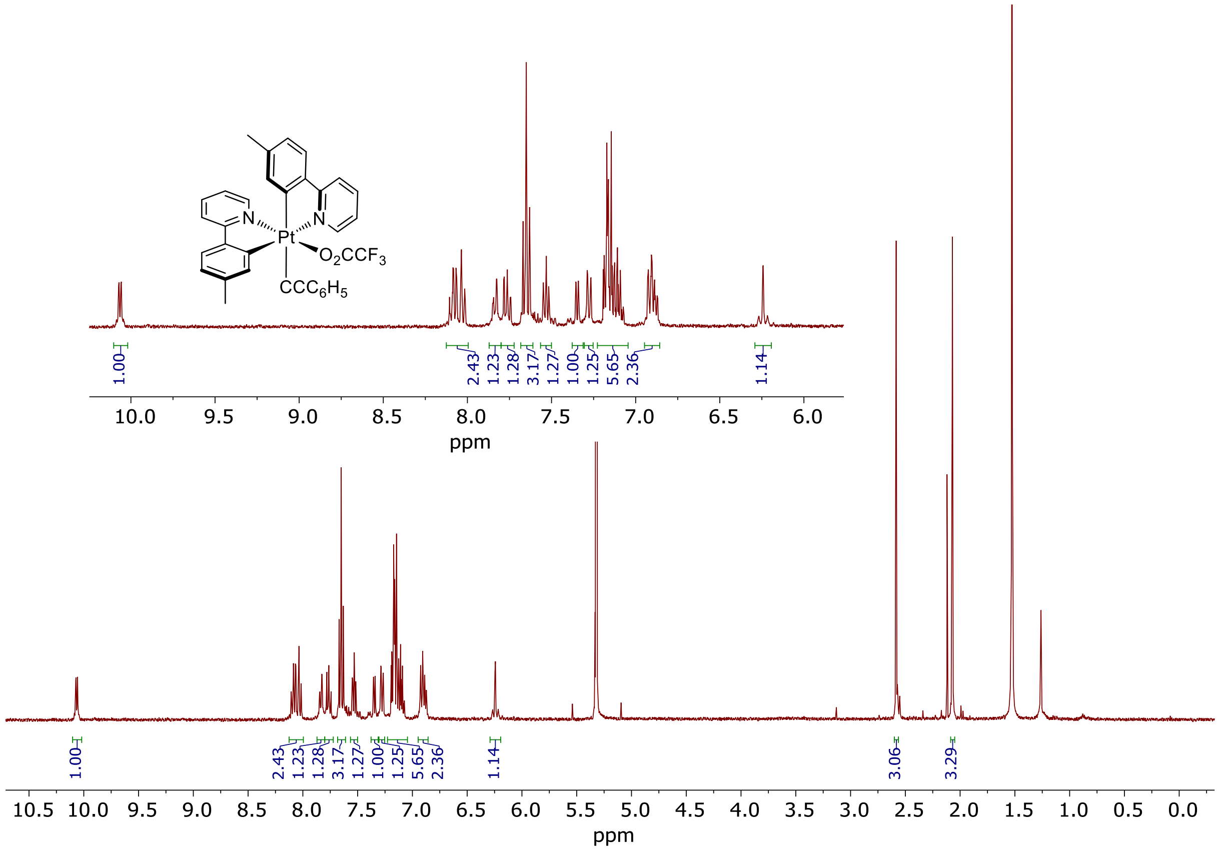

Figure S14. ^{1}H NMR spectrum of complex *mer*-**5b** (CD_2Cl_2, 400 MHz).

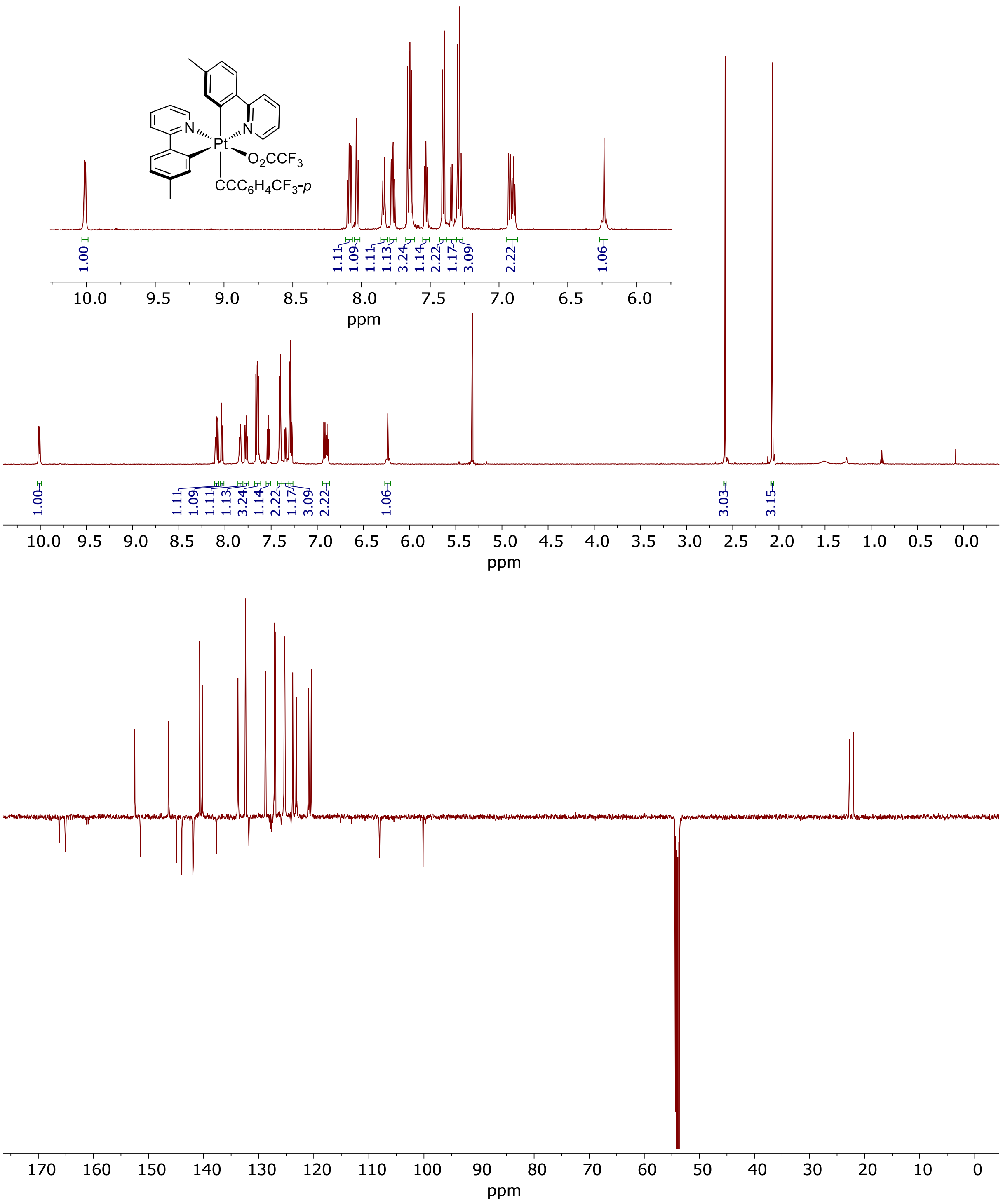

Figure S15. ^{1}H (top) and ^{13}C{^{1}H} APT (bottom) NMR spectra of complex *mer*-**5c** (CD_2Cl_2, 600 and 151 MHz, respectively).

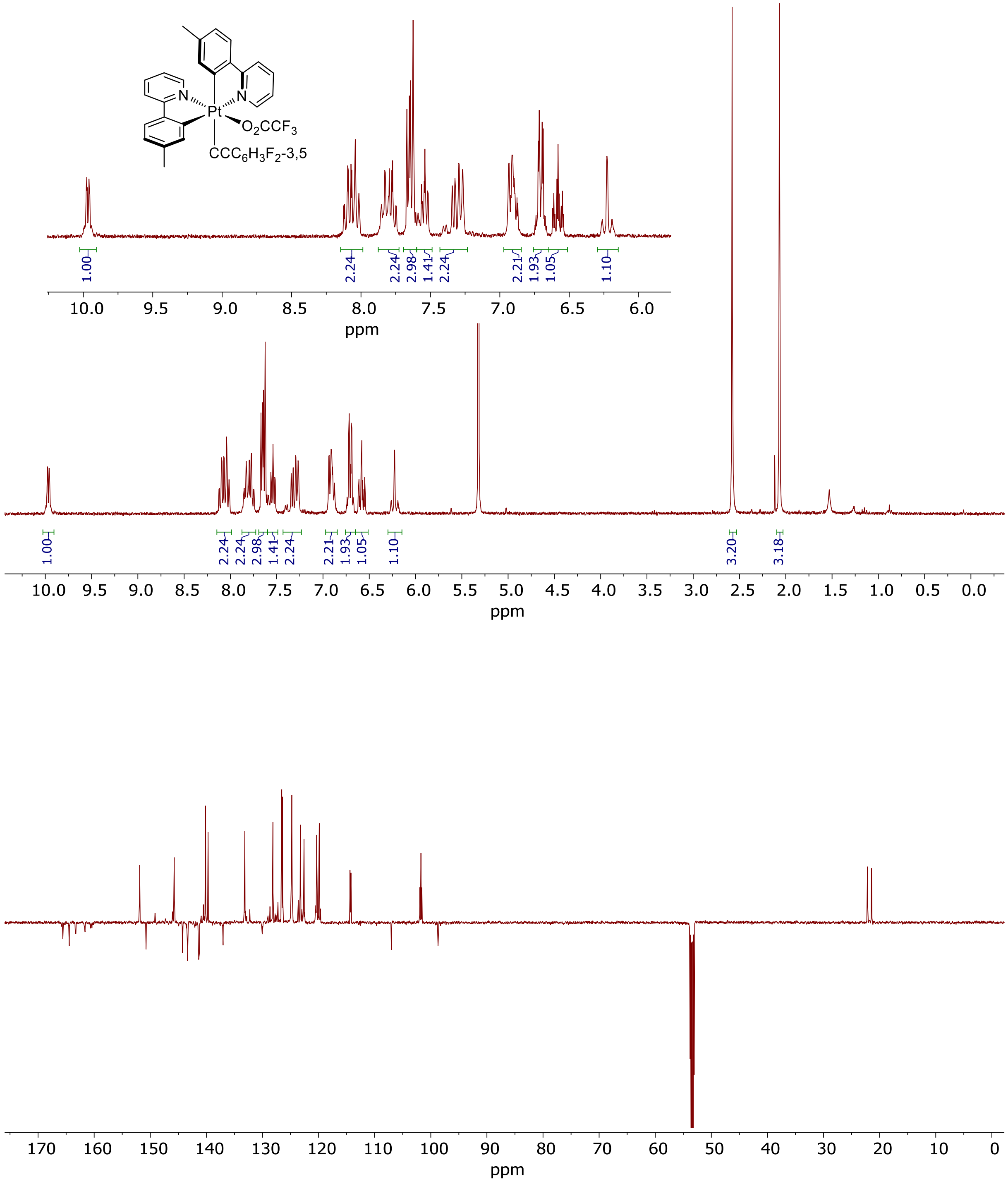

Figure S16. ^{1}H (top) and ^{13}C{^{1}H} APT (bottom) NMR spectra of complex *mer*-**5d** (CD_2Cl_2, 300 and 151 MHz, respectively).

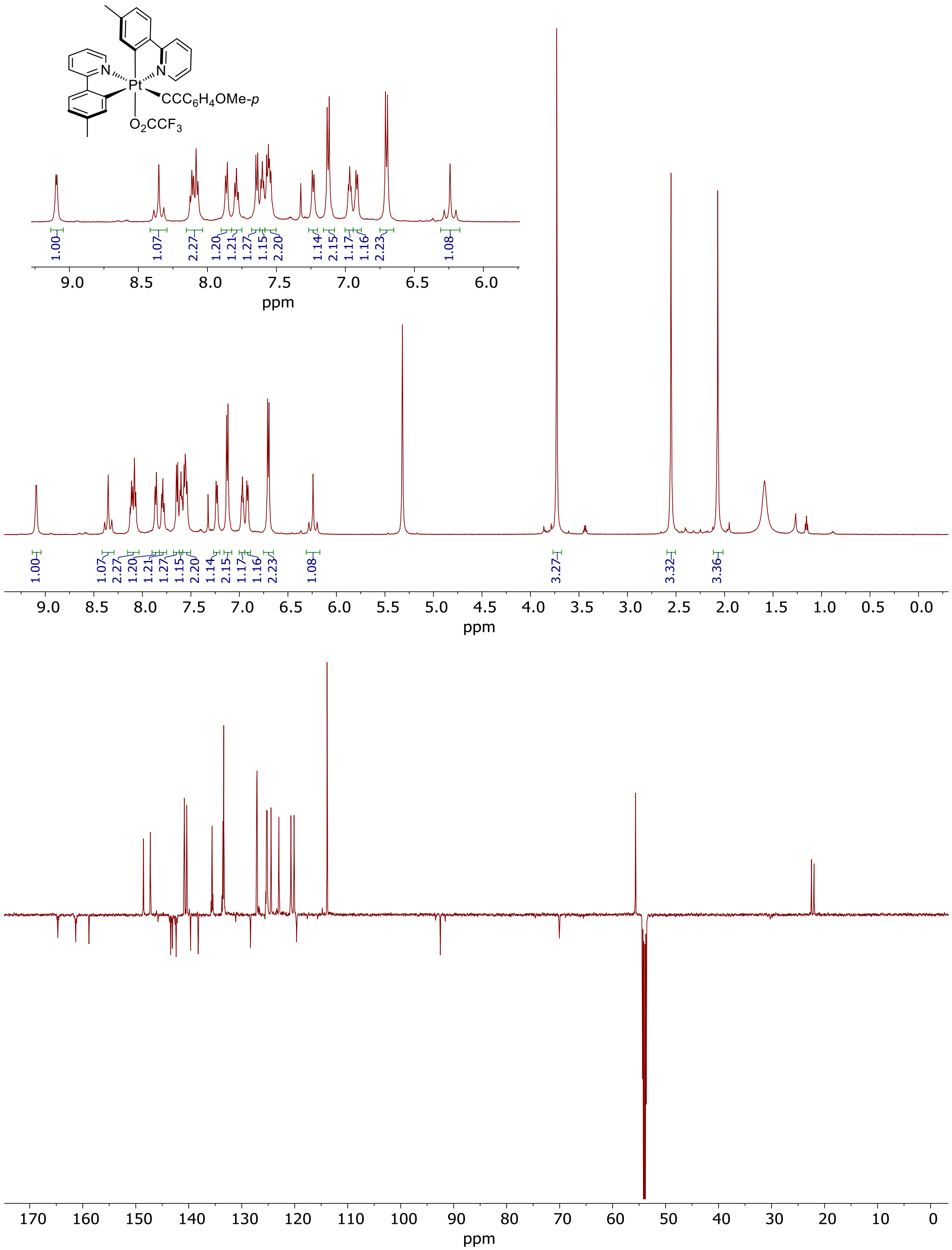

Figure S17. ^{1}H (top) and $^{13}C\{^{1}H\}$ APT (bottom) NMR spectra of complex *fac*-**5a** (CD_2Cl_2, 600 and 151 MHz, respectively).

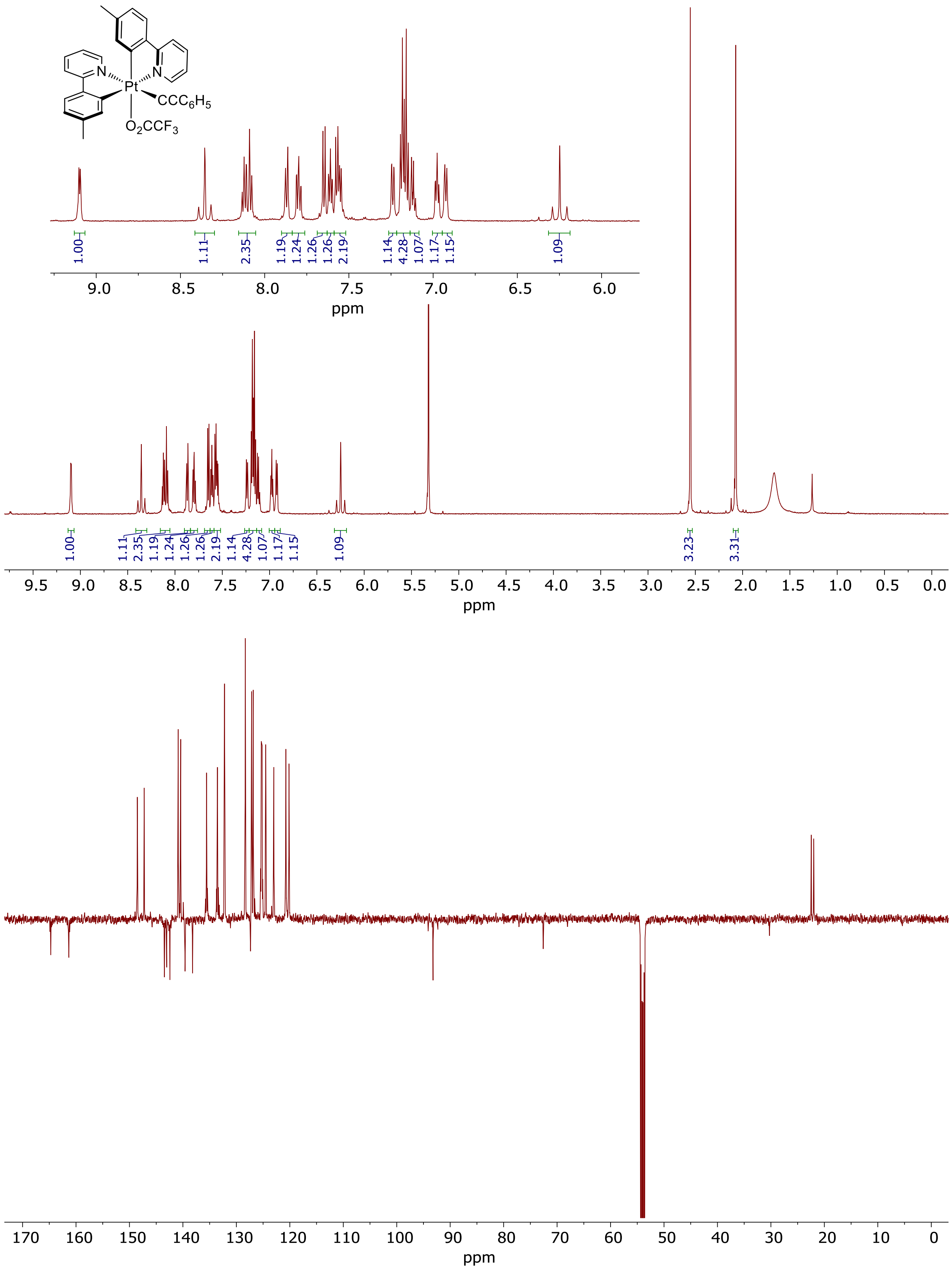

Figure S18. ^{1}H (top) and $^{13}C\{^{1}H\}$ APT (bottom) NMR spectra of complex *fac*-**5b** (CD_2Cl_2, 600 and 151 MHz, respectively).

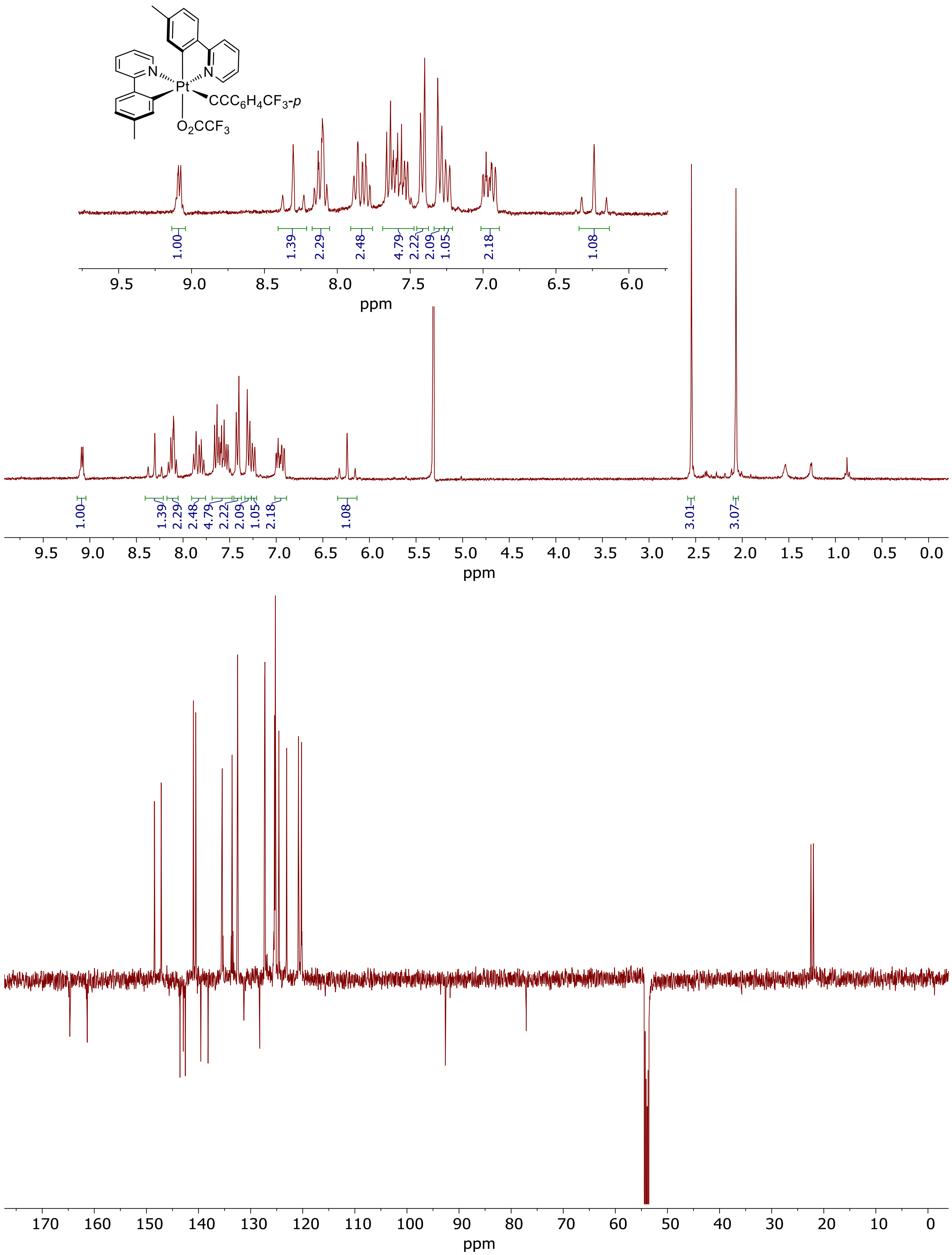

Figure S19. ^{1}H (top) and $^{13}C\{^{1}H\}$ APT (bottom) NMR spectra of complex *fac*-**5c** (CD_2Cl_2, 600 and 151 MHz, respectively).

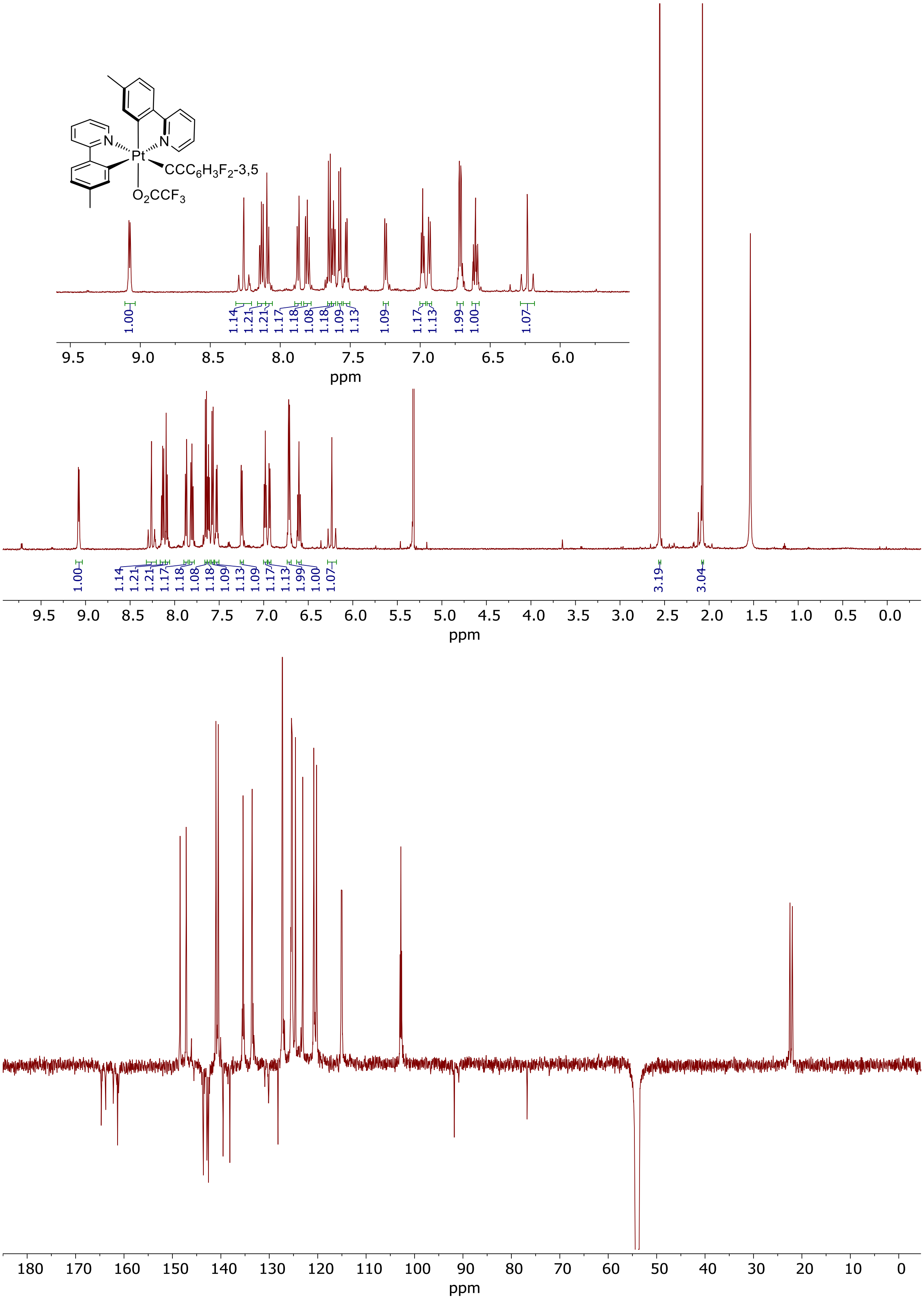

Figure S20. ^{1}H (top) and ^{13}C{^{1}H} APT (bottom) NMR spectra of complex *fac*-**5d** (CD_2Cl_2, 600 and 151 MHz, respectively).

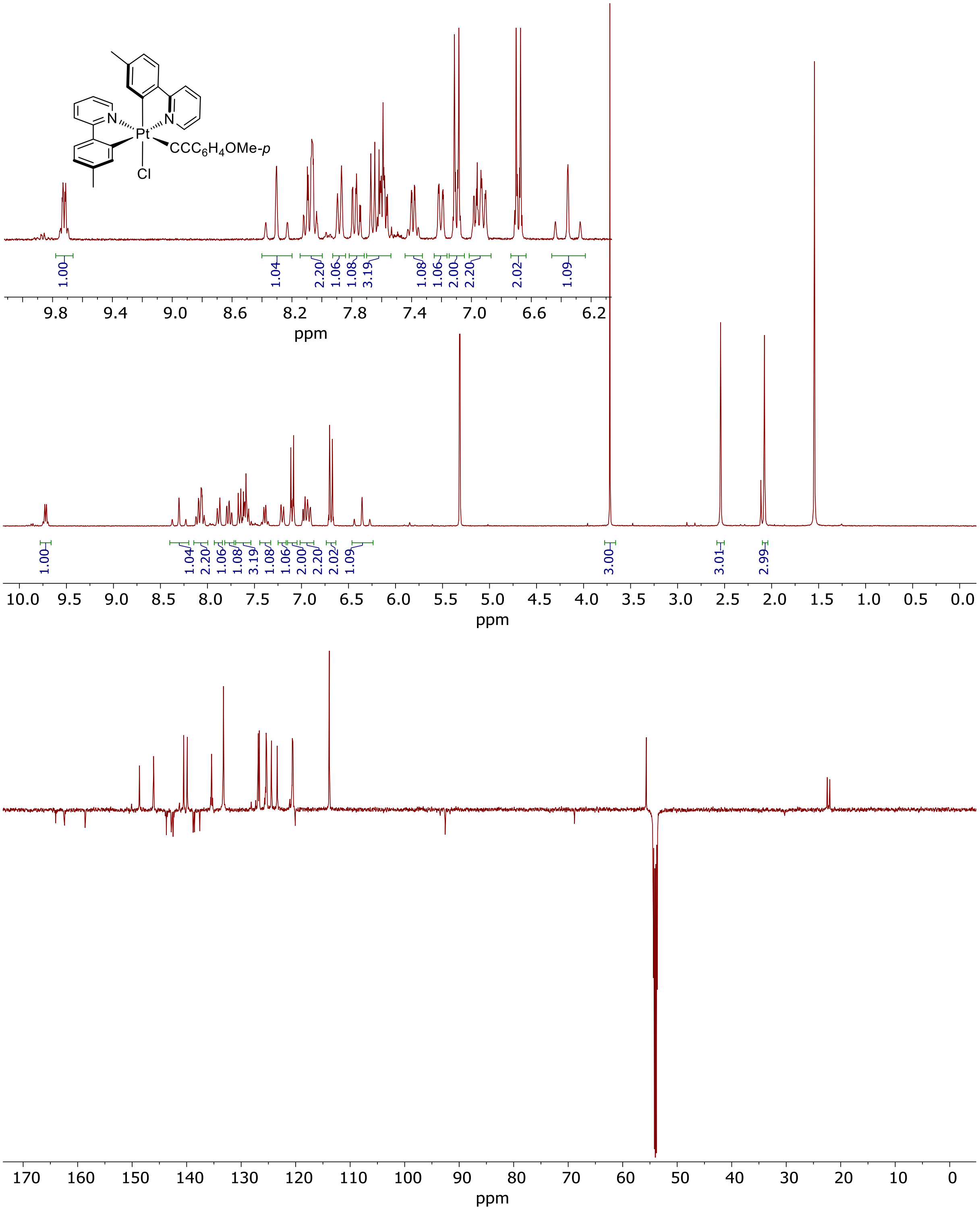

Figure S21. ^{1}H (top) and ^{13}C{^{1}H} APT (bottom) NMR spectra of complex *fac*-**6a** (CD_2Cl_2, 300 and 151 MHz, respectively).

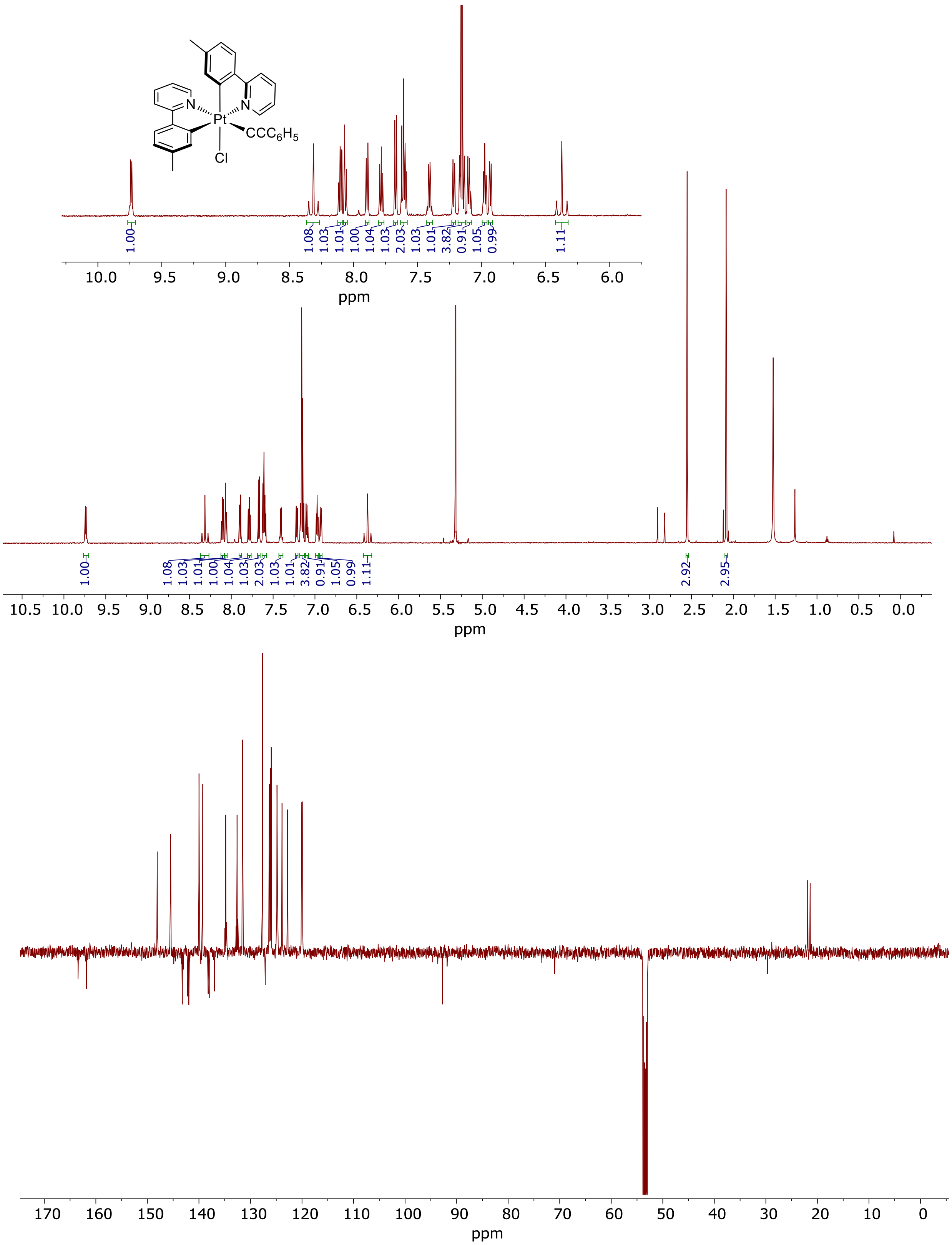

Figure S22. ^{1}H (top) and $^{13}C\{^{1}H\}$ APT (bottom) NMR spectra of complex *fac*-**6b** (CD_2Cl_2, 300 and 151 MHz, respectively).

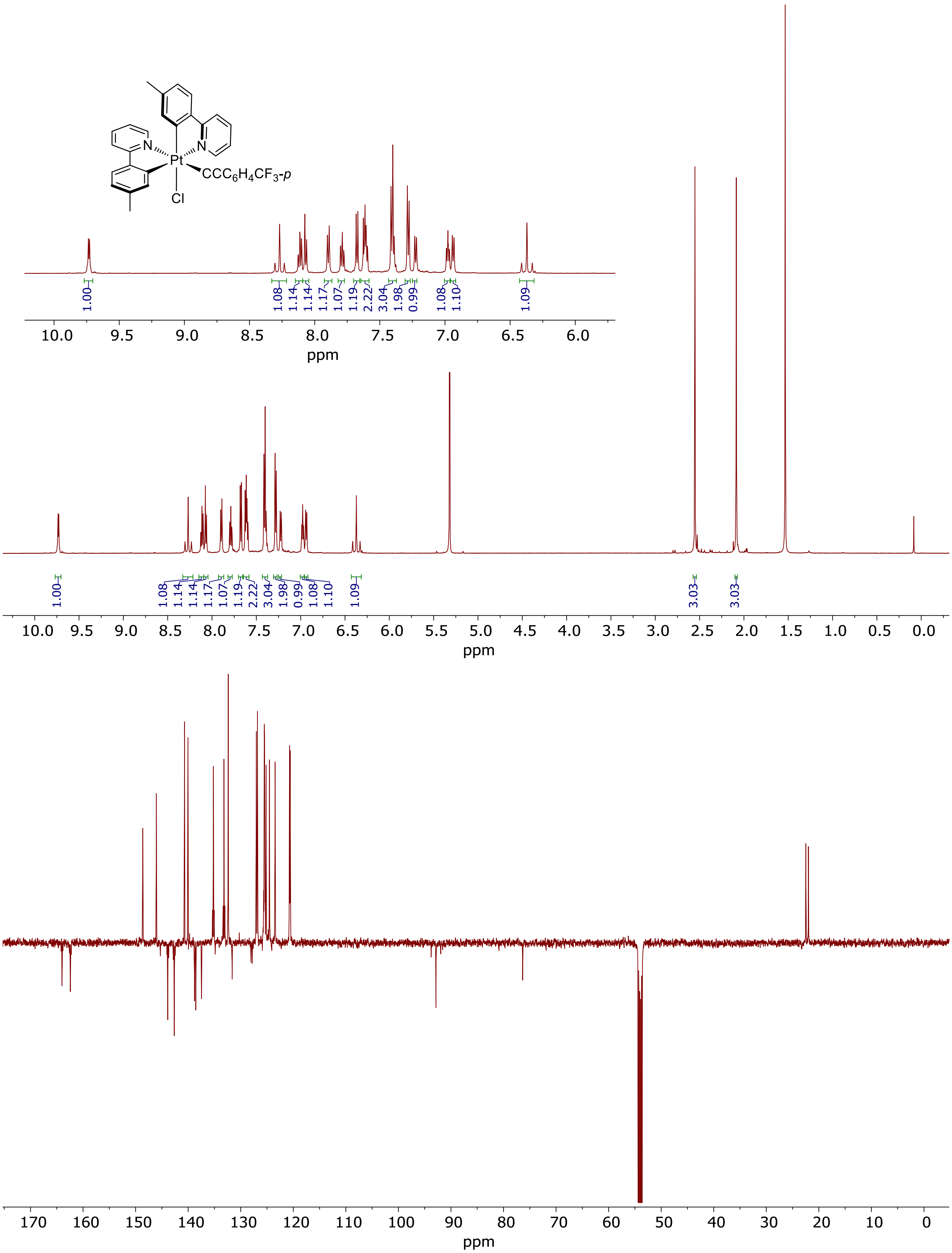

Figure S23. ^{1}H (top) and $^{13}C\{^{1}H\}$ APT (bottom) NMR spectra of complex *fac*-**6c** (CD_2Cl_2, 600 and 151 MHz, respectively).

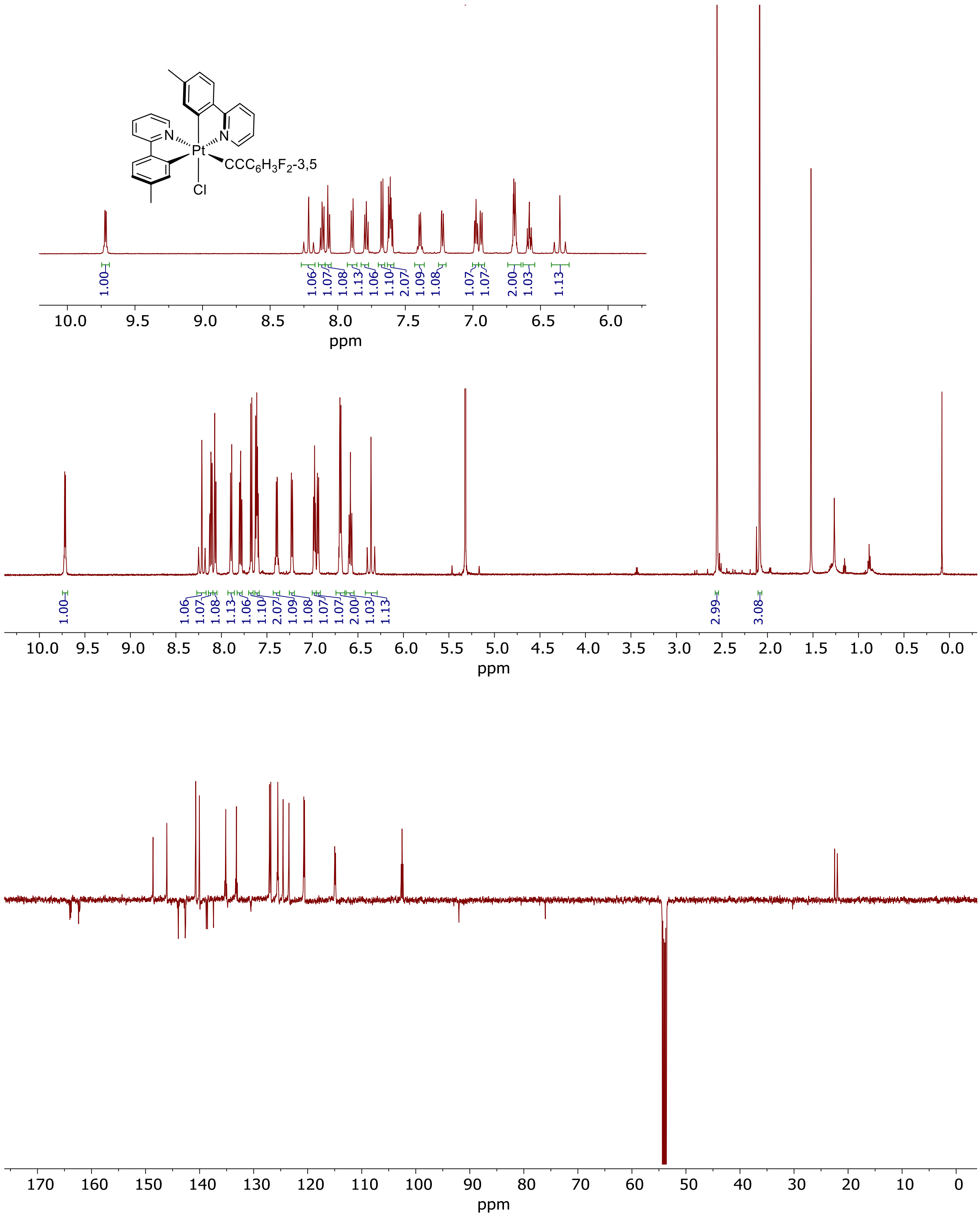

Figure S24. ^{1}H (top) and $^{13}C\{^{1}H\}$ APT (bottom) NMR spectra of complex *fac*-**6d** (CD_2Cl_2, 600 and 151 MHz, respectively).

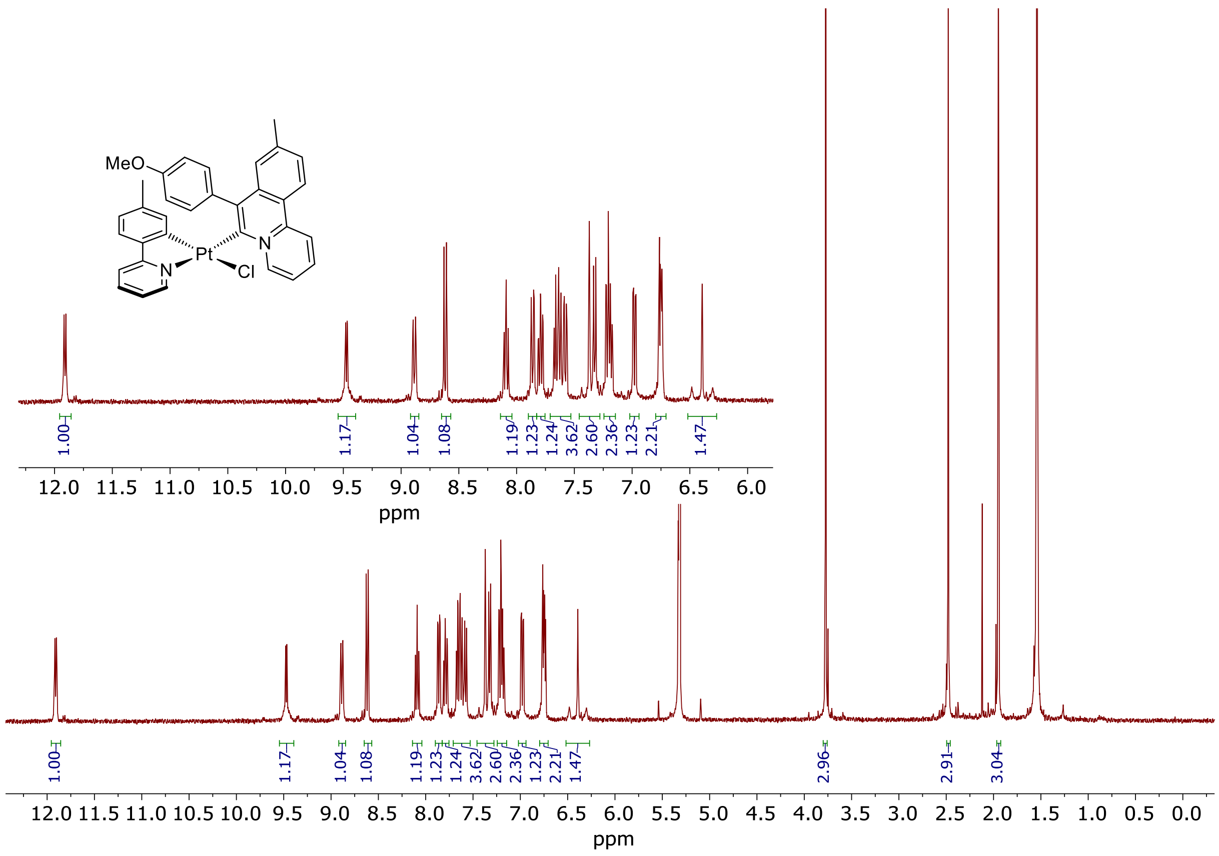

Figure S25. ^{1}H NMR spectrum of complex **7a** (CD_2Cl_2, 400 MHz).

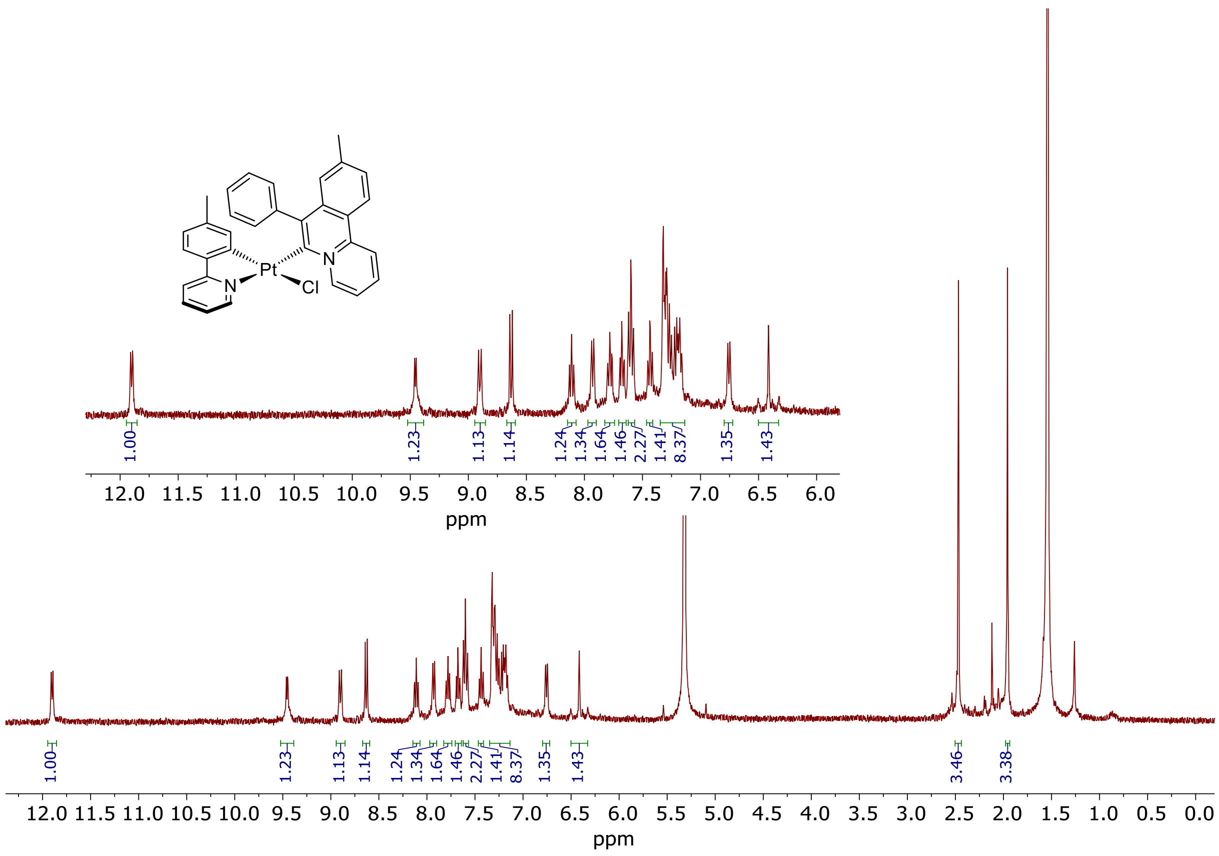

Figure S26. ^{1}H NMR spectrum of complex **7b** (CD_2Cl_2, 400 MHz).

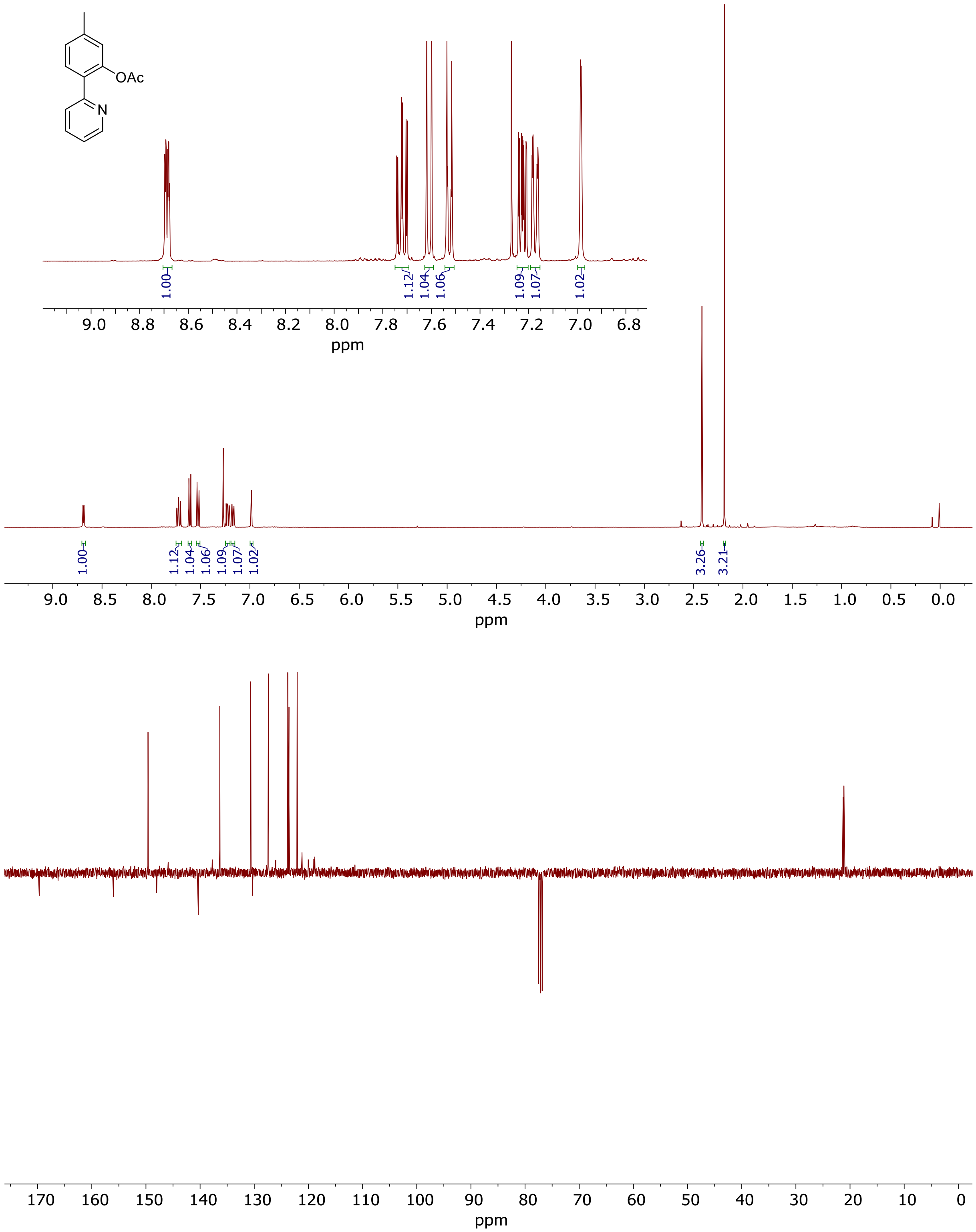

Figure S27. ^{1}H (top) and ^{13}C{^{1}H} APT (bottom) NMR spectra of compound **8** (CD_2Cl_2, 400 and 101 MHz, respectively).

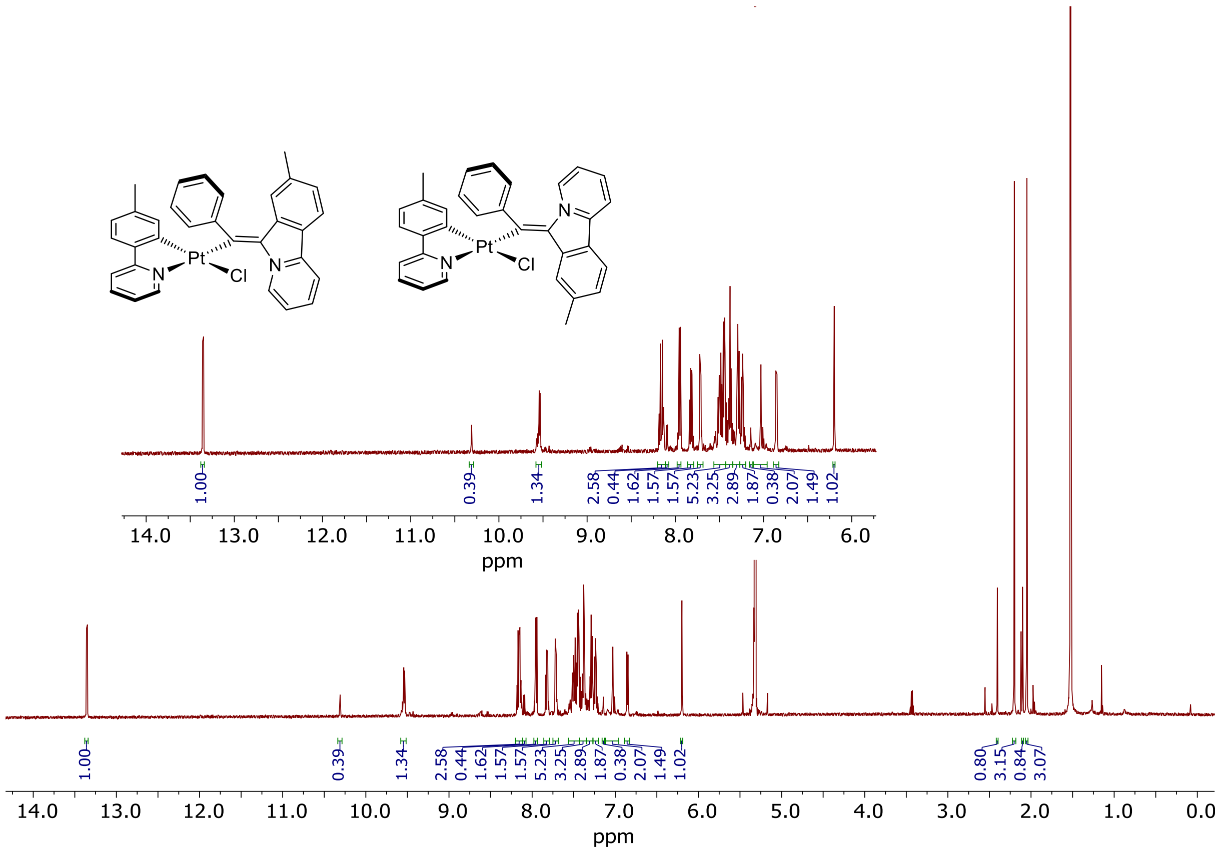

Figure S28. ^{1}H NMR spectrum of the mixture of isomers *(Z/E)*-**9b** (CD_2Cl_2, 600 MHz).

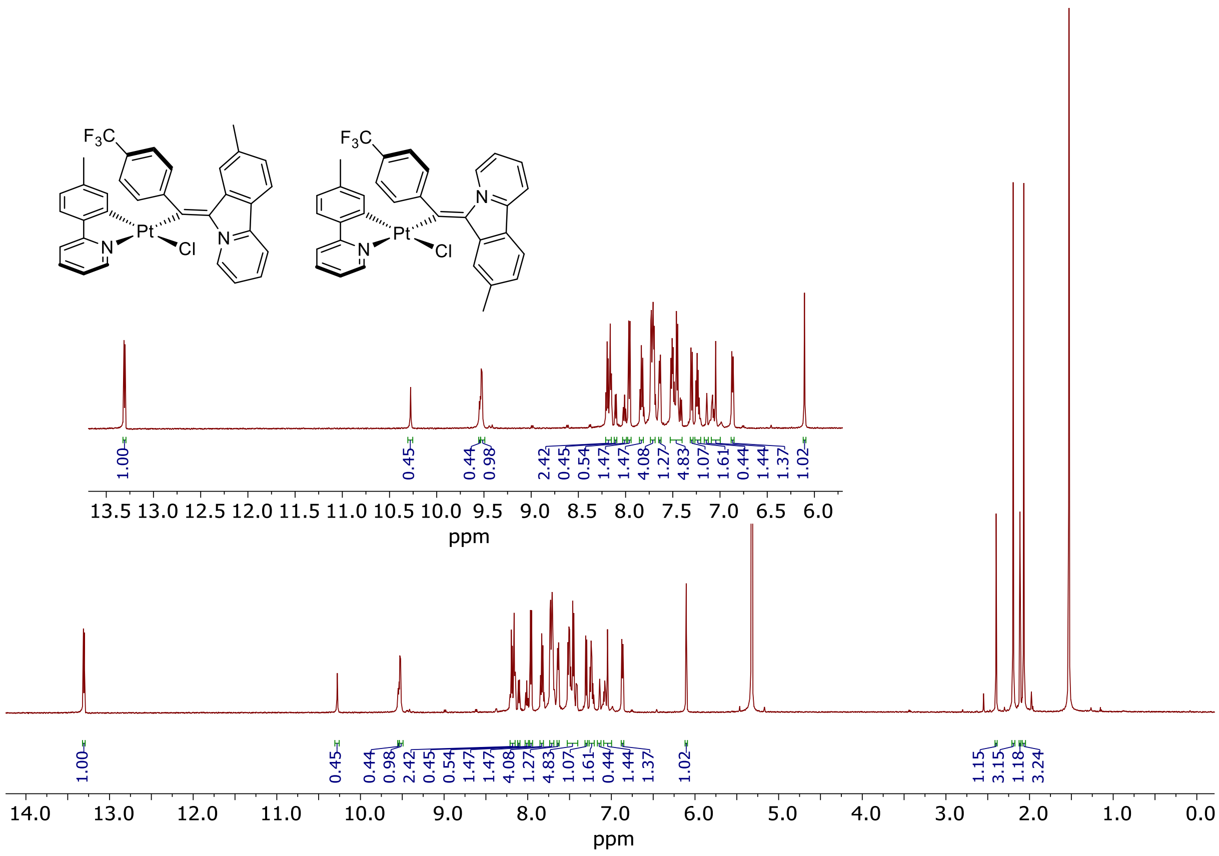

Figure S29. ^{1}H NMR spectrum of the mixture of isomers *(Z/E)*-**9c** (CD_2Cl_2, 600 MHz).

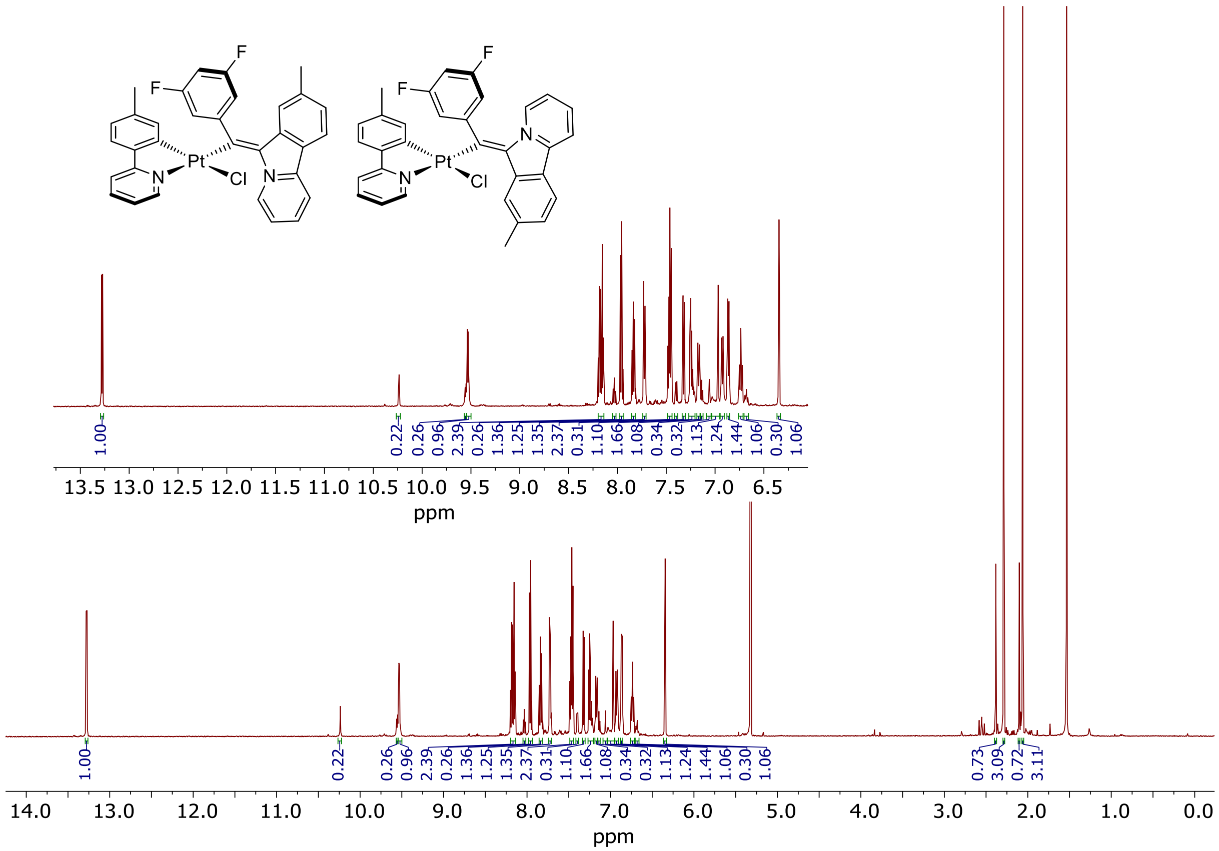

Figure S30. ^{1}H NMR spectrum of the mixture of isomers *(Z/E)*-**9d** (CD_2Cl_2, 400 MHz).

4. NMR spectra of crude reaction mixtures

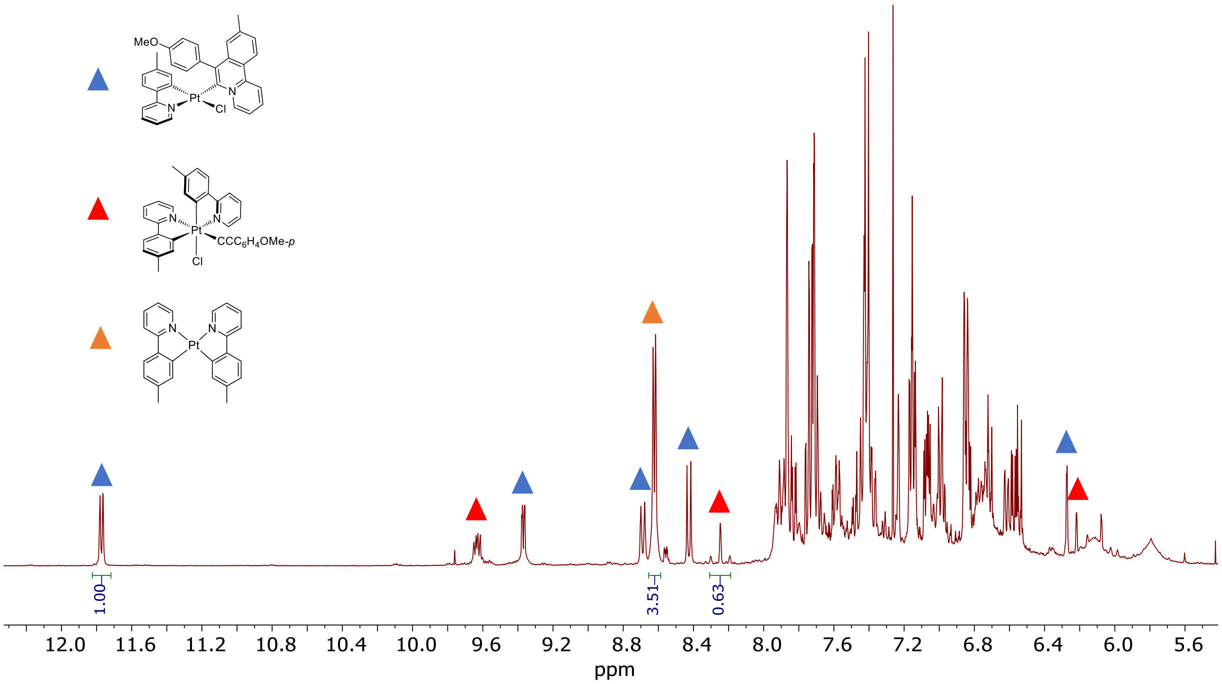

Figure S31. ^{1}H NMR spectrum (aromatic region) of the crude reaction mixture resulting from the irradiation of *mer*-**4a** with a 365 nm LED source after treatment with NH_4Cl ($CDCl_3$, 400 MHz).

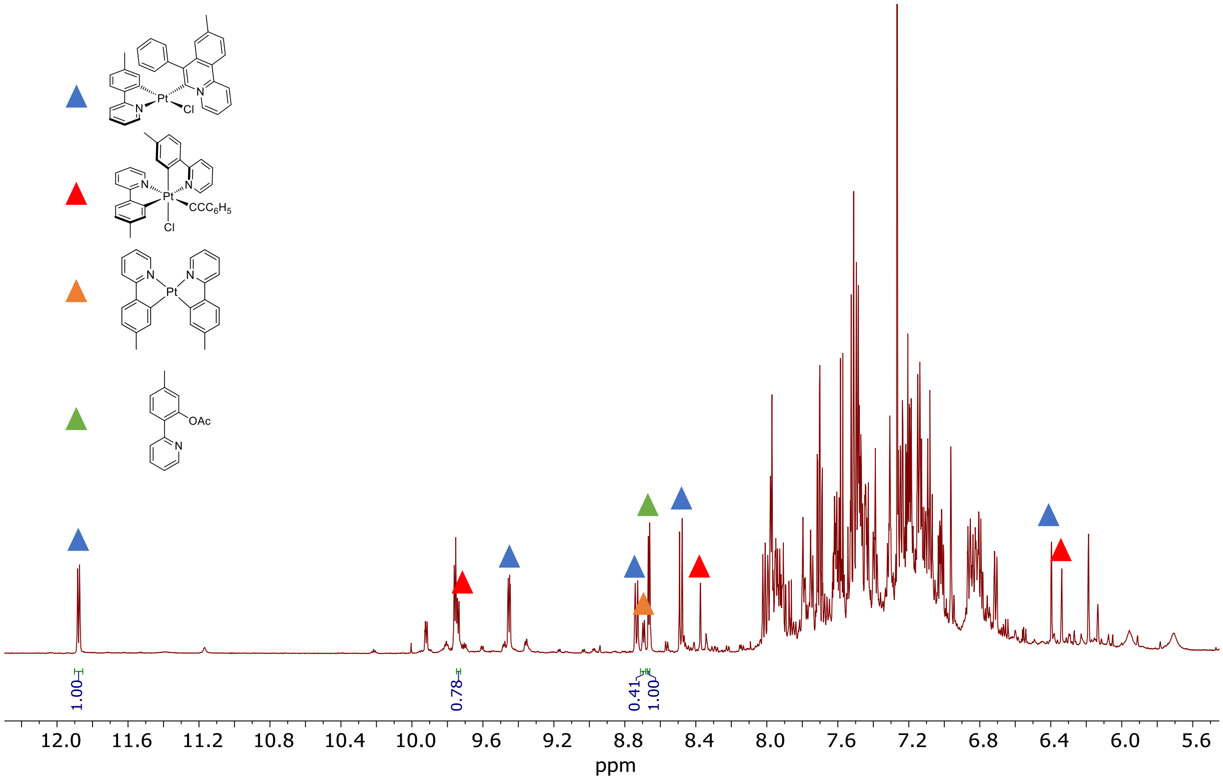

Figure S32. ^{1}H NMR spectrum (aromatic region) of the crude reaction mixture resulting from the irradiation of *mer*-**4b** with a 365 nm LED source after treatment with NH_4Cl ($CDCl_3$, 400 MHz).

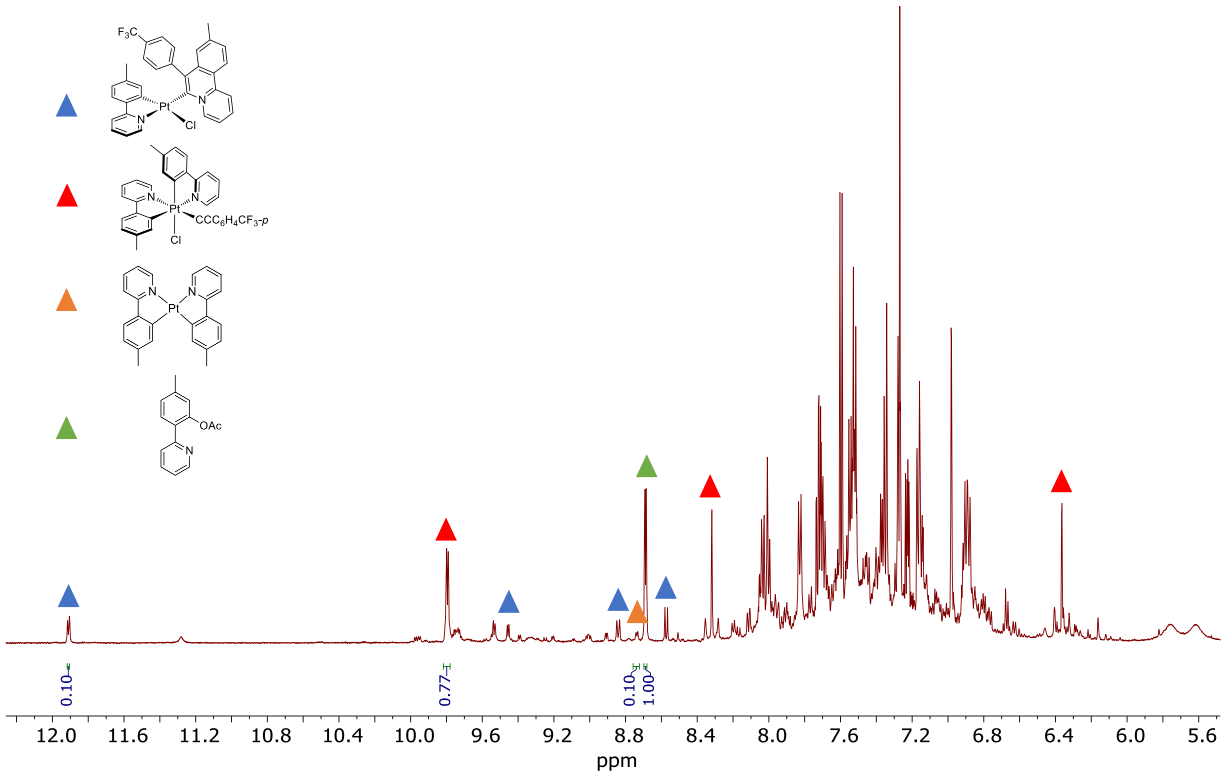

Figure S33. ¹H NMR spectrum (aromatic region) of the crude reaction mixture resulting from the irradiation of *mer*-**4c** with a 365 nm LED source after treatment with NH_4Cl ($CDCl_3$, 400 MHz).

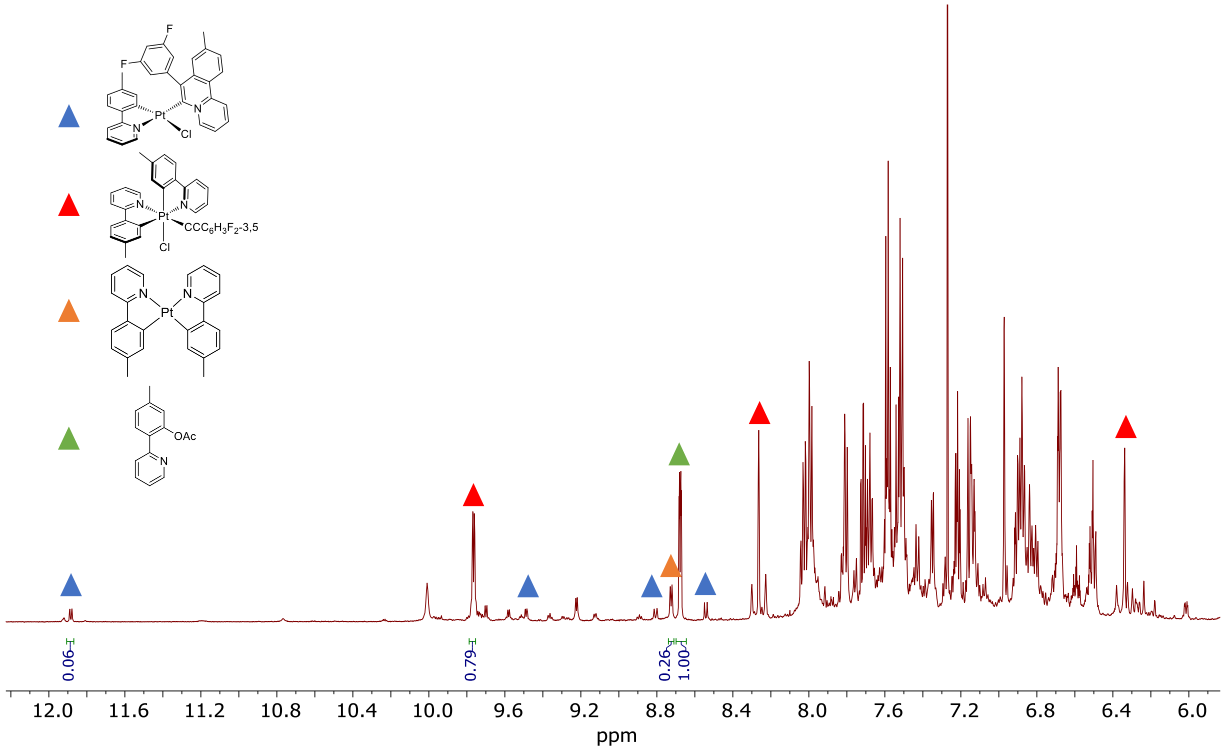

Figure S34. ¹H NMR spectrum (aromatic region) of the crude reaction mixture resulting from the irradiation of *mer*-**4d** with a 365 nm LED source after treatment with NH_4Cl ($CDCl_3$, 400 MHz).

5. Excitation and emission spectra

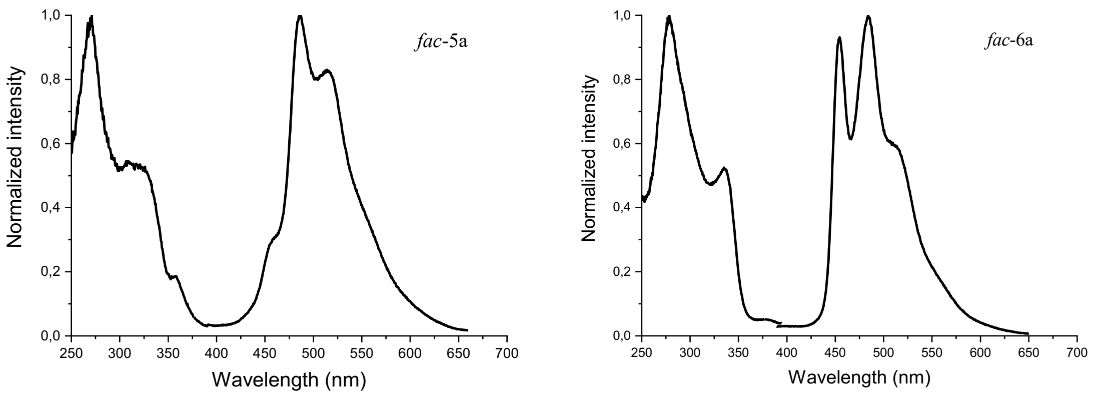

Figure S35. Excitation and emission spectra of complexes *fac*-**5a** and *fac*-**6a** in CH_3CN at 298 K.

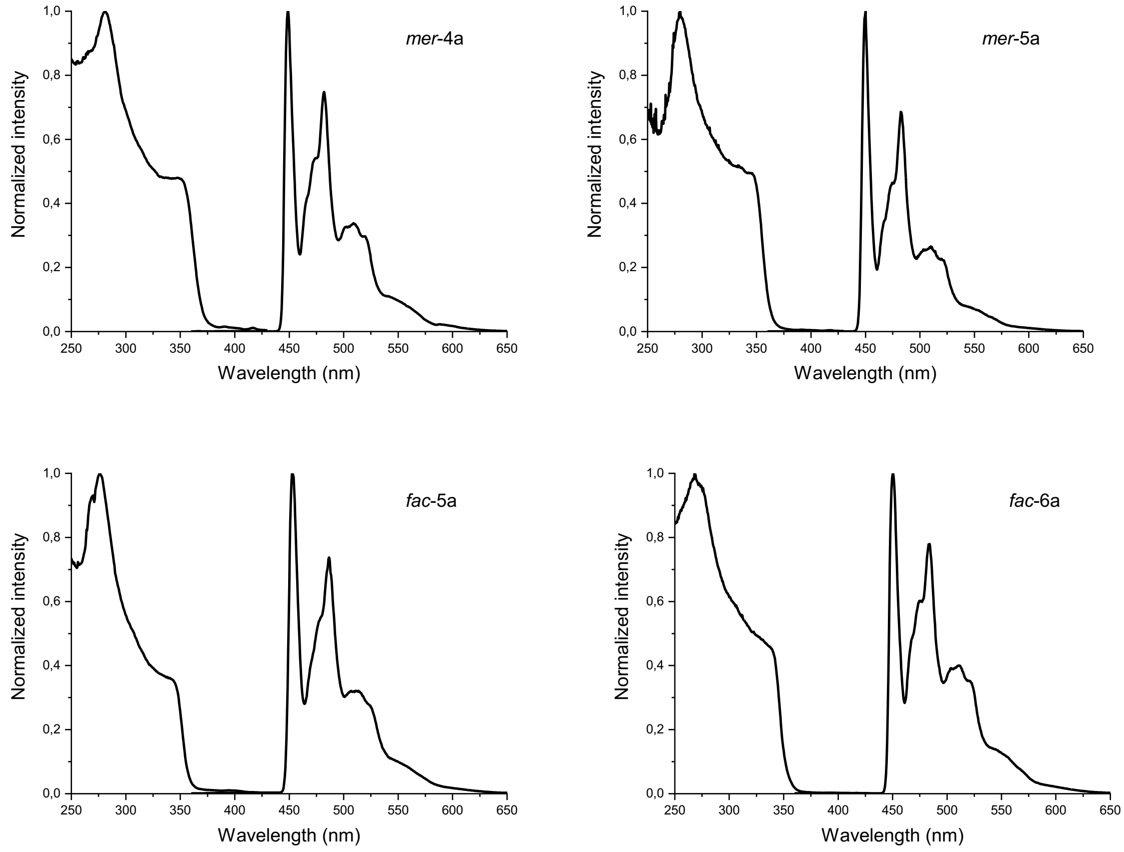

Figure S36. Excitation and emission spectra of complexes *mer*-**4a**, *mer*-**5a**, *fac*-**5a** and *fac*-**6a** in frozen 2-MeTHF at 77 K.

6. Computational methods

DFT calculations were carried out with Gaussian 16,[5] using the B3LYP functional[6,7] together with the 6-31G**[8,9] basis set for the light atoms and the LANL2DZ[10] basis set and effective core potential for the Pt atom. Optimizations were carried out without symmetry restrictions. Vertical excitation energies were obtained from TDDFT calculations at the ground-state optimized geometries. The solvent effect (MeCN) was accounted for in all cases by using the integral equation formalism variant of the polarizable continuum solvation model (IEFPCM).[11] All the optimized structures were confirmed as minima on the potential energy surface by performing frequency calculations (zero imaginary frequencies).

7. Computational data

7.1. Complex *mer*-4a

Table S6. Fragment contributions (%; from atomic orbital contributions) to the frontier orbitals of *mer*-**4a** in MeCN solution.

energy (a.u.)	number	L1	L2	L3	L4	Pt
–0.012	154 (LUMO+5)	4	9	73	0	14
–0.034	153 (LUMO+4)	9	89	0	0	1
–0.046	152 (LUMO+3)	88	11	0	0	1
–0.049	151 (LUMO+2)	29	5	2	16	48
–0.058	150 (LUMO+1)	2	96	0	0	2
–0.067	149 (LUMO)	97	0	0	0	1
–0.197	148 (HOMO)	0	1	95	0	4
–0.224	147 (HOMO–1)	1	74	18	1	6
–0.229	146 (HOMO–2)	62	17	12	5	4
–0.232	145 (HOMO–3)	28	17	39	12	5
–0.242	144 (HOMO–4)	10	41	0	45	3
–0.244	143 (HOMO–5)	67	13	8	8	3

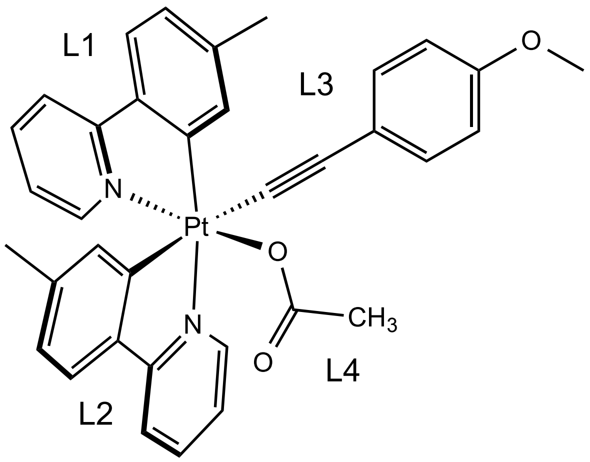

Figure S37. Ligand numbering in complex *mer*-**4a**.

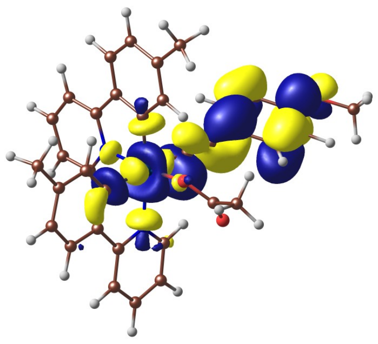

154 (LUMO+5)

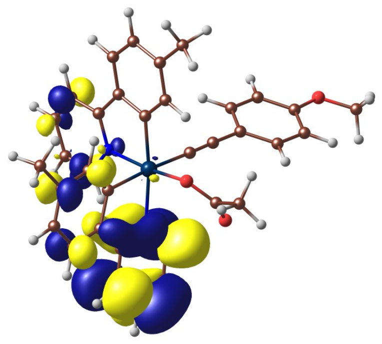

153 (LUMO+4)

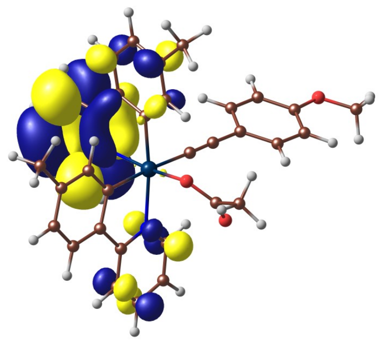

152 (LUMO+3)

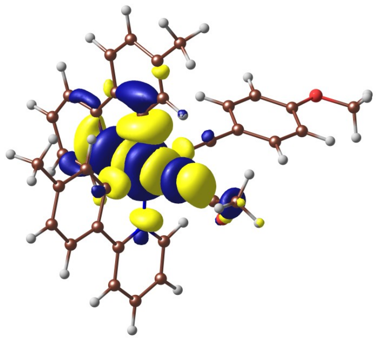

151 (LUMO+2)

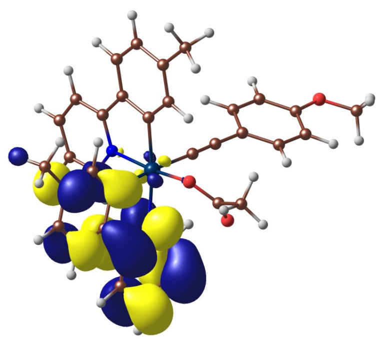

150 (LUMO+1)

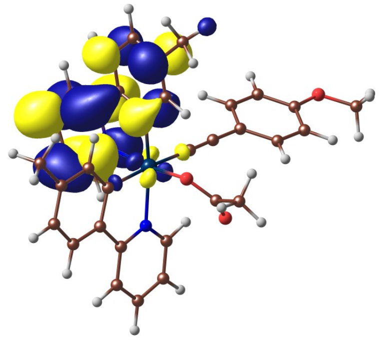

149 (LUMO)

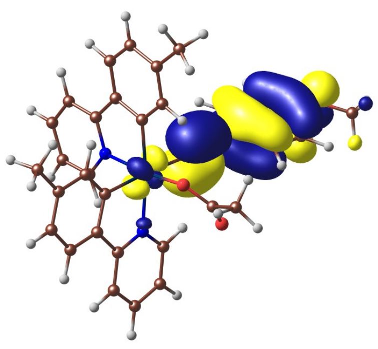

148 (HOMO)

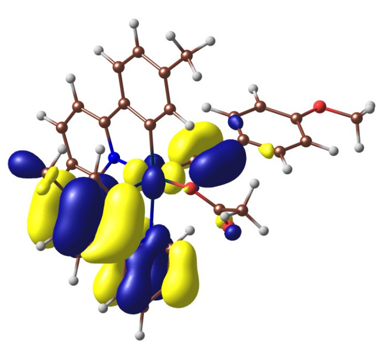

147 (HOMO–1)

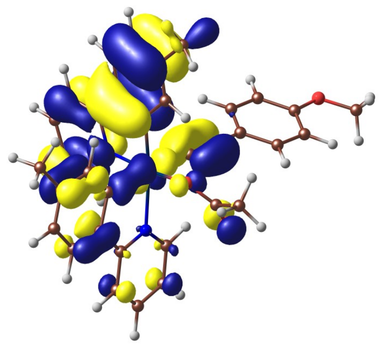

146 (HOMO–2)

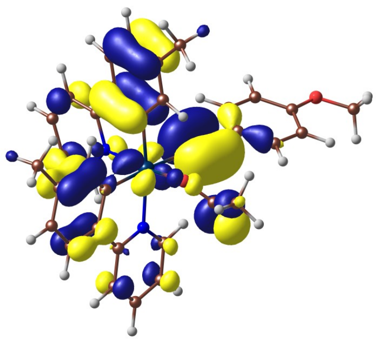

145 (HOMO–3)

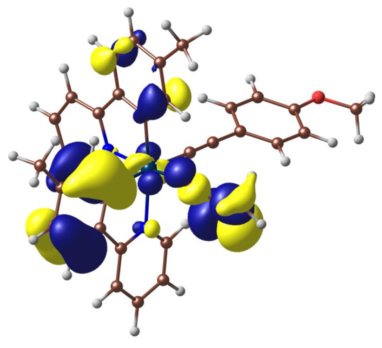

144 (HOMO–4)

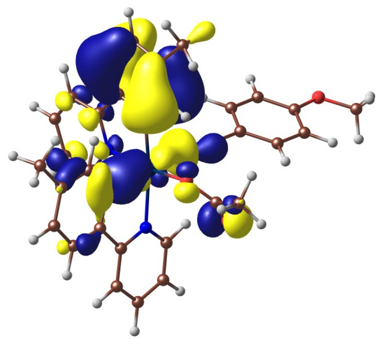

143 (HOMO–5)

Figure S38. Molecular orbital isosurfaces of *mer*-**4a** (0.03 e bohr^{-3}).

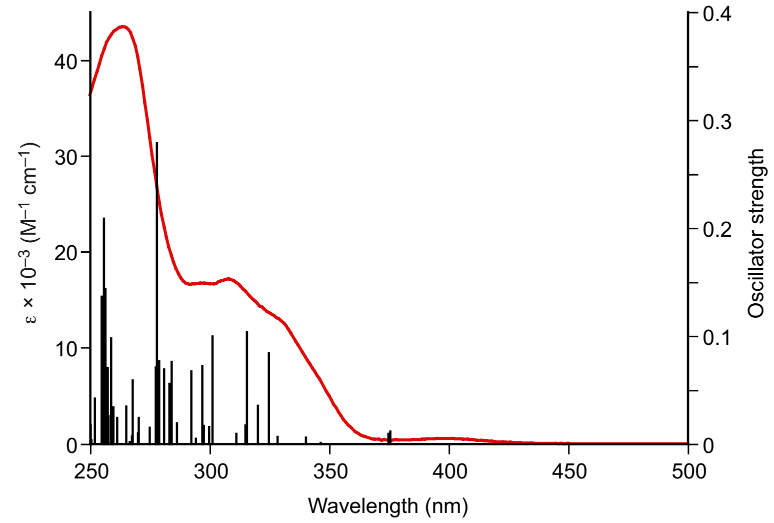

Figure S39. Calculated stick absorption spectrum of *mer*-**4a** compared with the experimental spectrum in MeCN solution (*ca.* 5 × 10^{-5} M) at 298 K.

Table S7. Lowest vertical singlet excitations of *mer*-**4a** from TDDFT calculations at the ground state geometry in MeCN solution.

State	Monoexcitations (Coefficient)	ΔE/eV	λ/nm	Oscillator strength	Main character
S1	148 → 149 (0.704)	2.986	415.2	0.0002	LLCT (L3 → L1)
S2	148 → 150 (0.286)<br>148 → 151 (0.633)	3.302	375.5	0.0117	LMCT (L3 → Pt)/LLCT (L3 → L2)
S3	148 → 150 (0.643)<br>148 → 151 (-0.283)	3.309	374.7	0.0094	LLCT (L3 → L1)/LMCT (L3 → Pt)
S4	148 → 152 (0.703)	3.579	346.5	0.0009	LLCT (L3 → L1)
S5	145 → 149 (-0.113)<br>146 → 149 (-0.171)<br>147 → 149 (0.671)	3.644	340.3	0.006	LLCT (L2 → L1)
S6	145 → 151 (-0.203)<br>146 → 149 (-0.200)<br>146 → 151 (-0.251)<br>147 → 151 (0.563)	3.776	328.3	0.0067	LMCT (L2 → Pt)
S7	143 → 149 (0.129)<br>146 → 149 (0.622)<br>147 → 149 (0.156)<br>147 → 151 (0.193)	3.819	324.7	0.0842	LC (L1)
S8	145 → 149 (0.672)<br>147 → 149 (0.120)	3.872	320.2	0.0355	LC (L1)/LLCT (L3 → L1)
S9	146 → 151 (0.101)<br>147 → 150 (0.595)<br>148 → 153 (-0.268)	3.929	315.6	0.104	LC (L2)
S10	147 → 150 (0.240)<br>148 → 153 (0.649)	3.937	314.9	0.0171	LLCT (L3 → L2)

Table S8. Lowest vertical triplet excitations of *mer*-**4a** from TDDFT calculations at the ground state geometry in MeCN solution.

State	Monoexcitations (Coefficient)	ΔE/eV	λ/nm	Main character
T1	143 → 149 (-0.193)<br>145 → 149 (-0.284)<br>146 → 149 (0.506)<br>146 → 152 (0.154)<br>147 → 149 (-0.102)<br>148 → 149 (-0.122)	2.898	427.8	LC (L1)
T2	145 → 150 (0.190)<br>146 → 150 (0.208)<br>147 → 150 (0.557)<br>147 → 153 (0.161)	2.928	423.5	LC (L2)
T3	148 → 149 (0.690)	2.980	416.1	LLCT (L3 → L1)
T4	141 → 155 (-0.165)<br>148 → 151 (0.393)<br>148 → 154 (0.491)<br>148 → 157 (-0.140)<br>148 → 158 (-0.151)	3.064	404.7	LC (L3)/LMCT (L3 → Pt)
T5	139 → 151 (0.106)<br>141 → 155 (0.107)<br>146 → 151 (-0.132)<br>148 → 150 (0.154)<br>148 → 151 (0.523)<br>148 → 154 (-0.336)	3.231	383.7	LMCT (L3 → Pt)

7.2. Complex *mer*-4b

Table S9. Fragment contributions (%; from atomic orbital contributions) to the frontier orbitals of *mer*-**4b** in MeCN solution.

energy (a.u.)	number	L1	L2	L3	L4	Pt
–0.020	146 (LUMO+5)	1	5	85	0	8
–0.034	145 (LUMO+4)	10	89	0	0	1
–0.047	144 (LUMO+3)	88	11	0	0	1
–0.049	143 (LUMO+2)	29	5	2	16	48
–0.058	142 (LUMO+1)	2	96	0	0	2
–0.068	141 (LUMO)	97	0	0	0	1
–0.210	140 (HOMO)	0	2	92	0	5
–0.224	139 (HOMO–1)	0	78	15	0	5
–0.230	138 (HOMO–2)	69	13	10	5	4
–0.233	137 (HOMO–3)	22	17	41	14	5
–0.243	136 (HOMO–4)	10	42	0	45	3
–0.244	135 (HOMO–5)	68	13	8	8	3

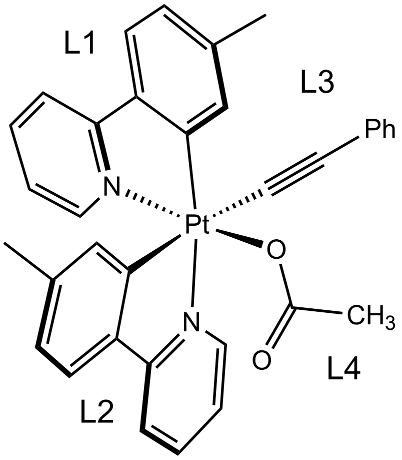

Figure S40. Ligand numbering in complex *mer*-**4b**.

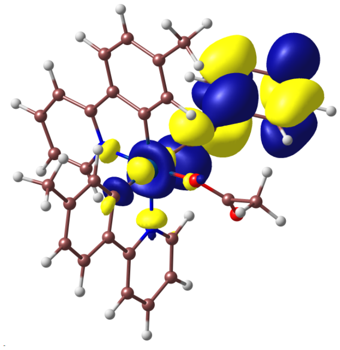

146 (LUMO+5)

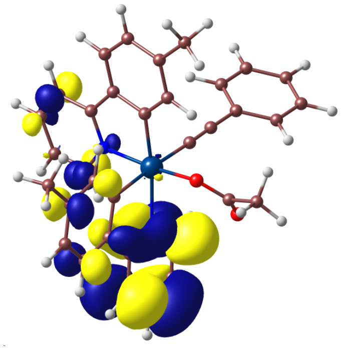

145 (LUMO+4)

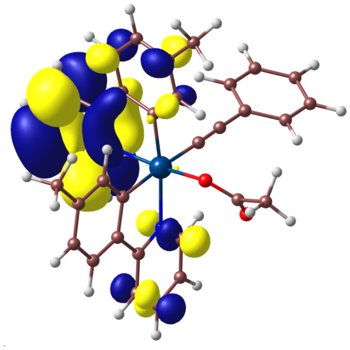

144 (LUMO+3)

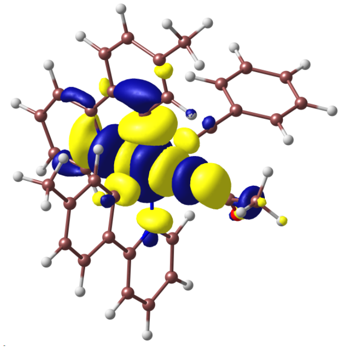

143 (LUMO+2)

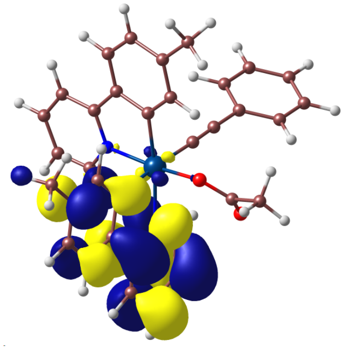

142 (LUMO+1)

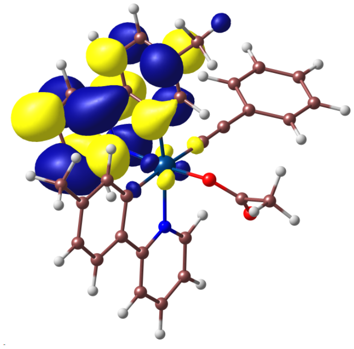

141 (LUMO)

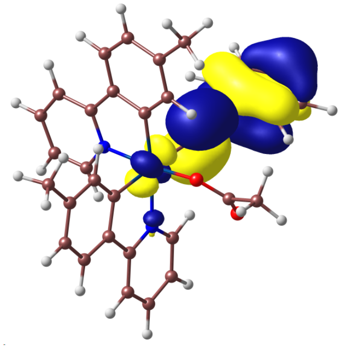

140 (HOMO)

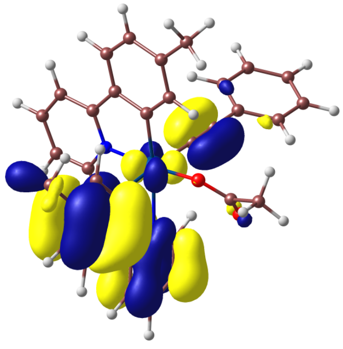

139 (HOMO–1)

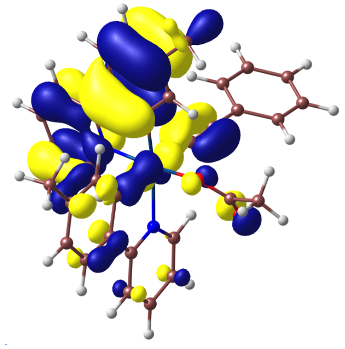

138 (HOMO–2)

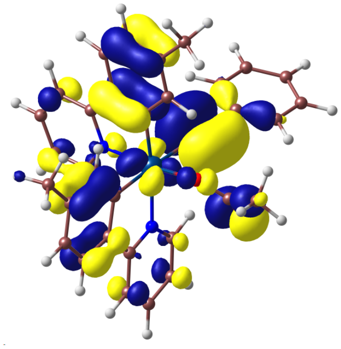

137 (HOMO–3)

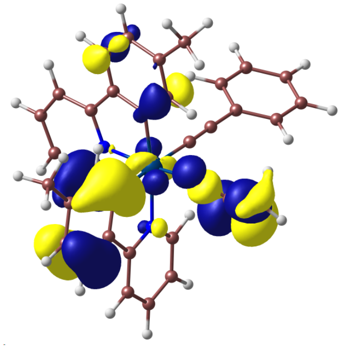

136 (HOMO–4)

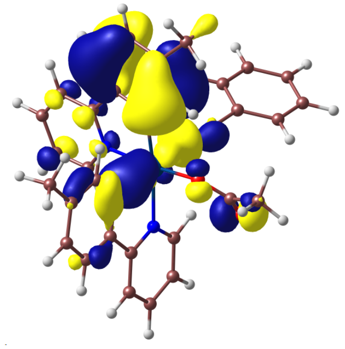

135 (HOMO–5)

Figure S41. Molecular orbital isosurfaces of *mer*-**4b** (0.03 e $bohr^{-3}$).

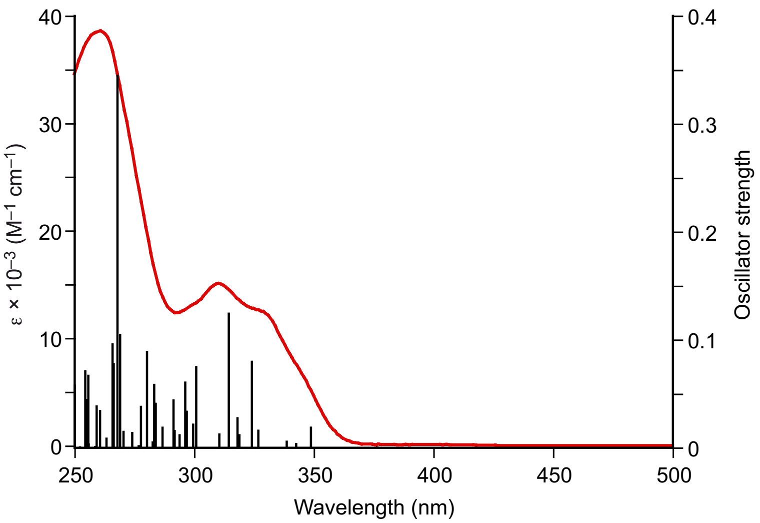

Figure S42. Calculated stick absorption spectrum of *mer*-**4b** compared with the experimental spectrum in MeCN solution (*ca.* 5×10^{-5} M) at 298 K.

Table S10. Lowest vertical singlet excitations of *mer*-**4b** from TDDFT calculations at the ground state geometry in MeCN solution.

State	Monoexcitations (Coefficient)	ΔE/eV	λ/nm	Oscillator strength	Main character
S1	140 → 141 (0.704)	3.294	376.4	0.0006	LLCT (L3 → L1)
S2	138 → 143 (-0.120)<br>140 → 143 (0.678)	3.555	348.7	0.0187	LMCT (L3 → Pt)
S3	140 → 142 (0.702)	3.619	342.6	0.0037	LLCT (L3 → L2)
S4	137 → 141 (-0.118)<br>138 → 141 (-0.163)<br>139 → 141 (0.672)	3.662	338.6	0.0056	LLCT (L2 → L1)
S5	137 → 143 (-0.236)<br>138 → 141 (-0.285)<br>138 → 143 (-0.214)<br>139 → 141 (-0.104)<br>139 → 143 (0.514)<br>140 → 143 (-0.122)	3.795	326.7	0.0157	LMCT (L2 → Pt)
S6	135 → 141 (0.125)<br>138 → 141 (0.585)<br>139 → 141 (0.140)<br>139 → 143 (0.264)	3.825	324.2	0.0798	LC (L1)
S7	137 → 141 (0.536)<br>140 → 144 (-0.430)	3.889	318.8	0.0118	LLCT (L3 → L1)
S8	137 → 141 (0.407)<br>139 → 142 (0.140)<br>140 → 144 (0.543)	3.898	318.1	0.0275	LLCT (L3 → L1)
S9	137 → 142 (-0.112)<br>137 → 143 (-0.102)<br>138 → 143 (0.151)	3.944	314.4	0.1244	LC (L2)

	139 → 142 (0.623)				
S10	135 → 143 (0.133)<br>137 → 143 (-0.270)<br>138 → 143 (0.567)<br>139 → 142 (-0.188)<br>139 → 143 (0.120)<br>140 → 143 (0.101)	3.993	310.5	0.0124	LMCT (L1 → Pt)

Table S11. Lowest vertical triplet excitations of *mer*-**4b** from TDDFT calculations at the ground state geometry in MeCN solution.

**State**	**Monoexcitations (Coefficient)**	**ΔE/eV**	**λ/nm**	**Main character**
T1	135 → 141 (-0.190)<br>137 → 141 (-0.244)<br>138 → 141 (0.539)<br>138 → 144 (0.165)	2.899	427.6	LC (L1)
T2	137 → 142 (0.180)<br>138 → 142 (0.170)<br>139 → 142 (0.573)<br>139 → 145 (0.167)	2.928	423.5	LC (L2)
T3	133 → 148 (-0.213)<br>140 → 143 (-0.236)<br>140 → 146 (0.593)<br>140 → 149 (0.103)	3.116	397.9	LC (L3)
T4	140 → 141 (0.693)	3.286	377.3	LLCT (L3 → L1)
T5	135 → 143 (-0.126)<br>138 → 143 (-0.253)<br>139 → 143 (0.198)<br>140 → 143 (0.533)<br>140 → 146 (0.200)	3.397	365.0	LMCT (L3 → Pt)

7.3. Complex *mer*-4d

Table S12. Fragment contributions (%; from atomic orbital contributions) to the frontier orbitals of *mer*-**4d** in MeCN solution.

energy (a.u.)	number	L1	L2	L3	L4	Pt
–0.031	154 (LUMO+5)	1	5	88	0	6
–0.035	153 (LUMO+4)	10	87	2	0	1
–0.047	152 (LUMO+3)	88	11	0	0	1
–0.051	151 (LUMO+2)	28	5	4	15	48
–0.059	150 (LUMO+1)	2	96	0	0	2
–0.068	149 (LUMO)	97	0	0	0	1
–0.219	148 (HOMO)	0	6	86	0	6
–0.226	147 (HOMO–1)	0	85	11	0	3
–0.231	146 (HOMO–2)	82	5	6	3	3
–0.236	145 (HOMO–3)	10	17	43	25	6
–0.243	144 (HOMO–4)	9	45	0	41	3
–0.245	143 (HOMO–5)	70	14	8	5	3

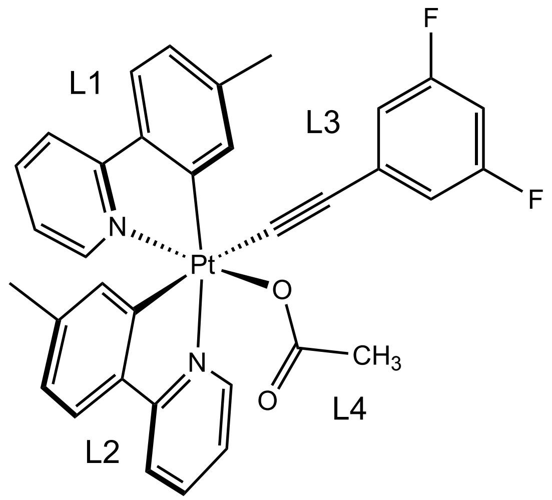

Figure S43. Ligand numbering in complex *mer*-**4d**.

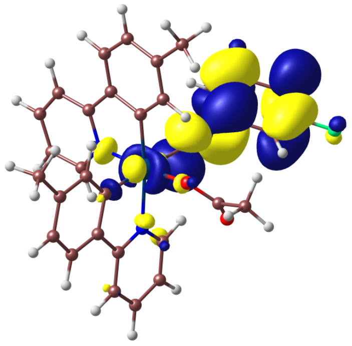

154 (LUMO+5)

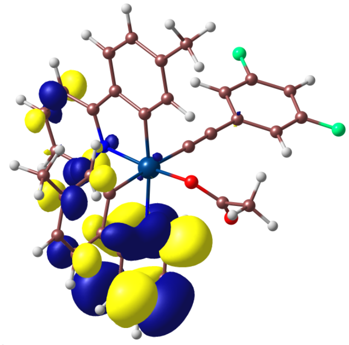

153 (LUMO+4)

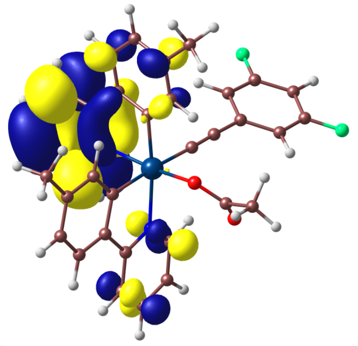

152 (LUMO+3)

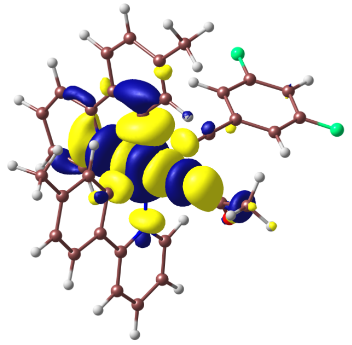

151 (LUMO+2)

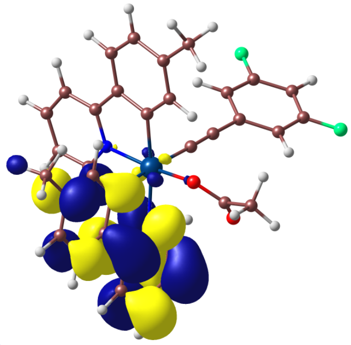

150 (LUMO+1)

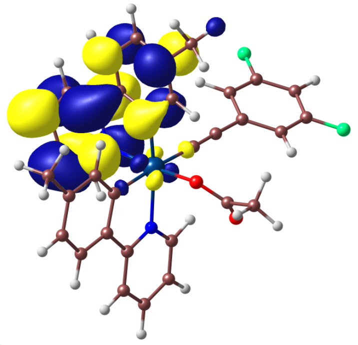

149 (LUMO)

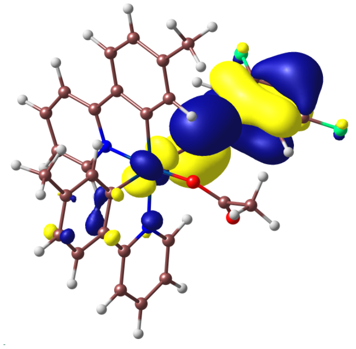

148 (HOMO)

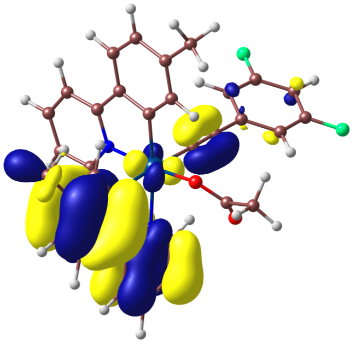

147 (HOMO–1)

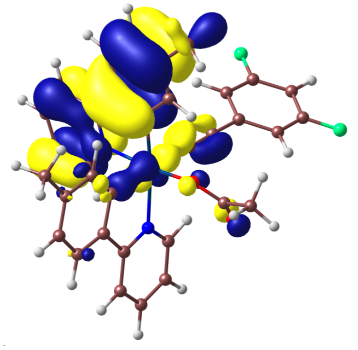

146 (HOMO–2)

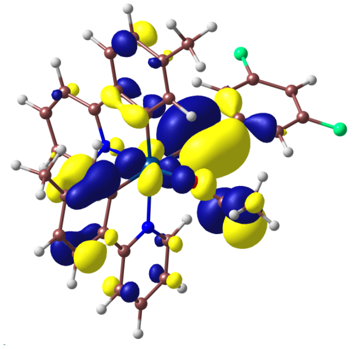

145 (HOMO–3)

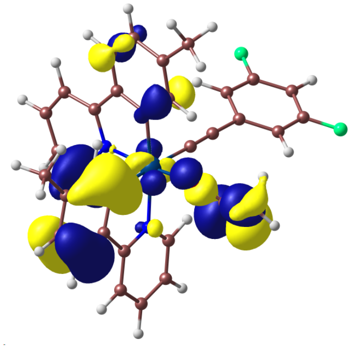

144 (HOMO–4)

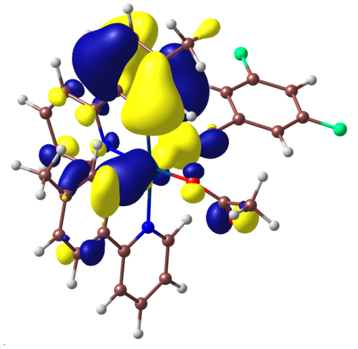

143 (HOMO–5)

Figure S44. Molecular orbital isosurfaces of *mer*-**4d** (0.03 e bohr^{-3}).

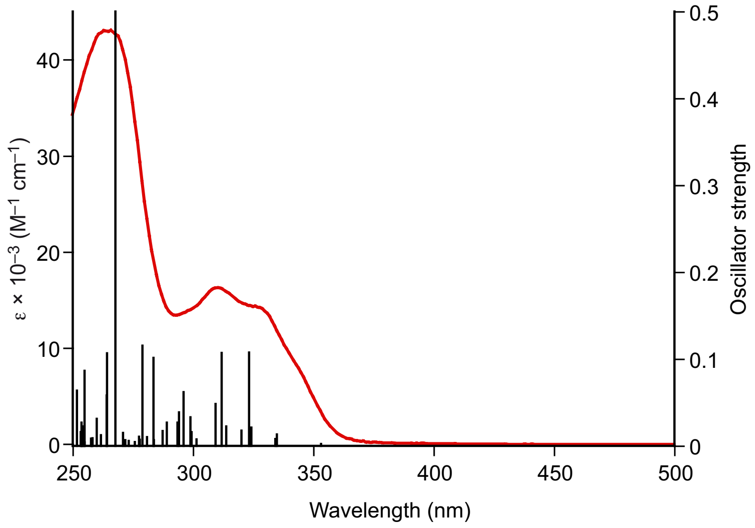

Figure S45. Calculated stick absorption spectrum of *mer*-**4d** compared with the experimental spectrum in MeCN solution (*ca.* 5 × 10^{-5} M) at 298 K.

Table S13. Selected vertical singlet excitations of *mer*-**4d** from TDDFT calculations at the ground state geometry in MeCN solution.

State	Monoexcitations (Coefficient)	ΔE/eV	λ/nm	Oscillator strength	Main character
S1	148 → 149 (0.702)	3.510	353.3	0.0025	LLCT (L3 → L1)
S2	146 → 151 (-0.180)<br>147 → 149 (-0.371)<br>147 → 151 (0.138)<br>148 → 151 (0.528)	3.703	334.9	0.0133	LMCT (L3 → Pt)/LLCT (L2 → L1)
S3	146 → 149 (-0.106)<br>146 → 151 (-0.134)<br>147 → 149 (0.577)<br>148 → 151 (0.323)	3.709	334.3	0.0081	LLCT (L2 → L1)/LMCT (L3 → Pt)
S4	146 → 149 (-0.335)<br>148 → 150 (0.594)	3.824	324.2	0.0212	LLCT (L3 → L2)
S5	143 → 149 (0.117)<br>146 → 149 (0.552)<br>147 → 149 (0.113)<br>148 → 150 (0.361)	3.836	323.3	0.1075	LC (L1)
S6	144 → 151 (0.102)<br>145 → 151 (-0.297)<br>146 → 149 (0.131)<br>147 → 151 (0.548)<br>148 → 151 (-0.226)	3.874	320.1	0.0175	LMCT (L2 → Pt)
S7	144 → 149 (-0.101)<br>145 → 149 (0.665)<br>147 → 149 (0.102)<br>147 → 150 (0.113)	3.952	313.8	0.0222	LLCT (L3 → L1)
S8	143 → 151 (0.107)	3.976	311.8	0.1071	LC (L2)

	145 → 149 (-0.136)<br>146 → 151 (0.325)<br>147 → 150 (0.541)<br>148 → 151 (0.108)				
S9	146 → 151 (0.539)<br>147 → 150 (-0.360)<br>148 → 151 (0.177)	4.007	309.4	0.0484	LMCT (L1 → Pt)
S10	143 → 149 (-0.102)<br>146 → 150 (0.120)<br>148 → 152 (0.676)	4.113	301.5	0.0075	LLCT (L3 → L1)

Table S14. Lowest vertical triplet excitations of *mer*-**4d** from TDDFT calculations at the ground state geometry in MeCN solution.

State	Monoexcitations (Coefficient)	ΔE/eV	λ/nm	Main character
T1	143 → 149 (-0.181)<br>145 → 149 (-0.130)<br>146 → 149 (0.582)<br>146 → 152 (0.180)	2.900	427.6	LC (L1)
T2	145 → 150 (0.130)<br>147 → 150 (0.589)<br>147 → 153 (0.174)<br>148 → 150 (0.156)	2.928	423.5	LC (L2)
T3	142 → 157 (-0.199)<br>147 → 154 (-0.130)<br>148 → 151 (-0.214)<br>148 → 154 (0.594)	3.114	398.2	LC (L3)
T4	143 → 151 (-0.147)<br>145 → 151 (-0.128)<br>146 → 151 (-0.290)<br>147 → 151 (0.180)<br>148 → 149 (0.268)<br>148 → 151 (0.450)<br>148 → 154 (0.131)	3.477	356.6	LMCT (L3 → Pt)
T5	143 → 149 (-0.108)<br>146 → 151 (0.133)<br>146 → 152 (0.104)<br>147 → 151 (-0.102)<br>148 → 149 (0.619)<br>148 → 151 (-0.160)	3.508	353.5	LLCT (L3 → L1)

7.4. Complex *mer*-5b

Table S15. Fragment contributions (%; from atomic orbital contributions) to the frontier orbitals of *mer*-**5b** in MeCN solution.

energy (a.u.)	number	L1	L2	L3	L4	Pt
–0.022	158 (LUMO+5)	2	6	81	0	10
–0.037	157 (LUMO+4)	9	89	0	0	1
–0.049	156 (LUMO+3)	88	10	0	0	1
–0.058	155 (LUMO+2)	27	18	2	11	42
–0.061	154 (LUMO+1)	4	84	0	3	8
–0.071	153 (LUMO)	97	0	0	0	1
–0.212	152 (HOMO)	0	2	93	0	5
–0.227	151 (HOMO–1)	0	85	10	0	4
–0.233	150 (HOMO–2)	83	8	5	0	4
–0.237	149 (HOMO–3)	10	27	54	3	7
–0.247	148 (HOMO–4)	77	10	9	0	3
–0.250	147 (HOMO–5)	3	74	17	4	2

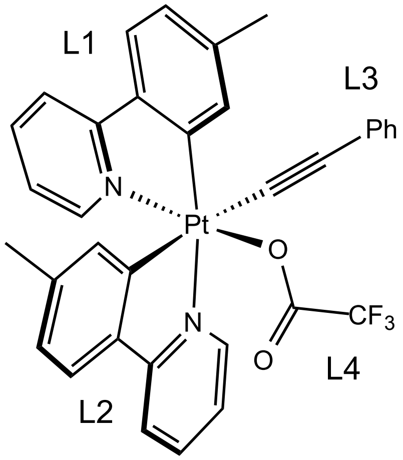

Figure S46. Ligand numbering in complex *mer*-**5b**.

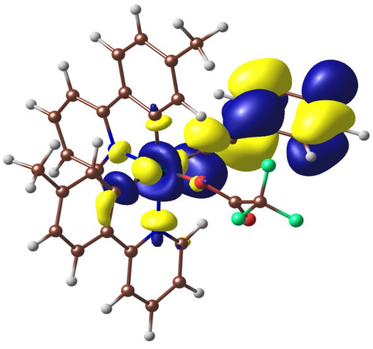

158 (LUMO+5)

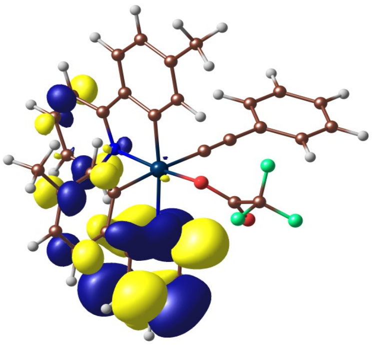

157 (LUMO+4)

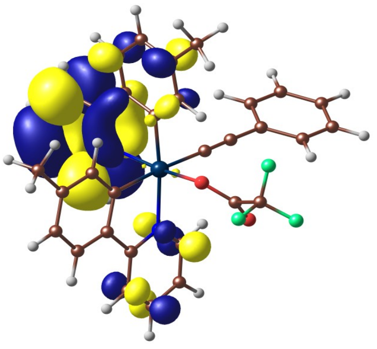

156 (LUMO+3)

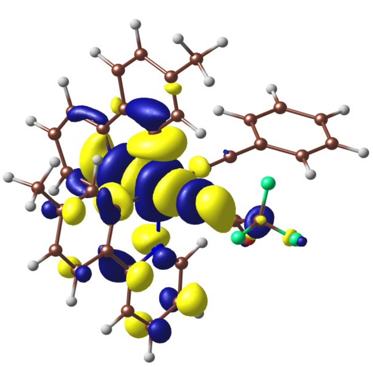

155 (LUMO+2)

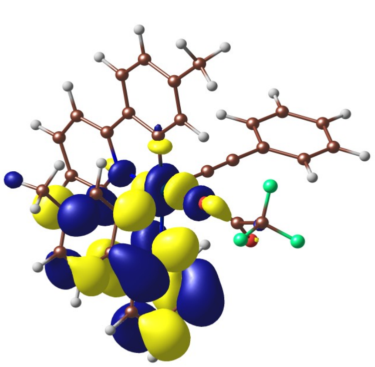

154 (LUMO+1)

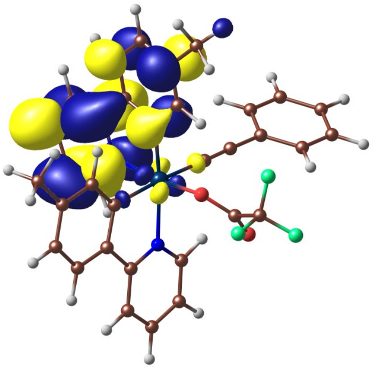

153 (LUMO)

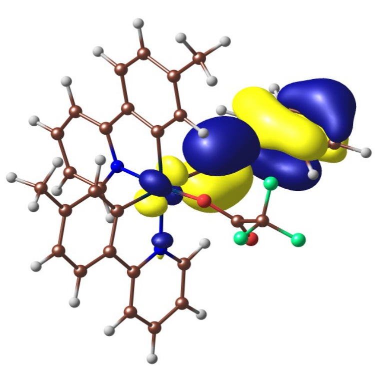

152 (HOMO)

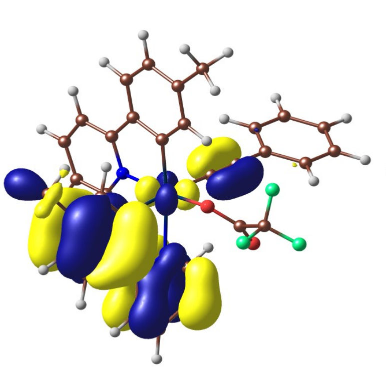

151 (HOMO–1)

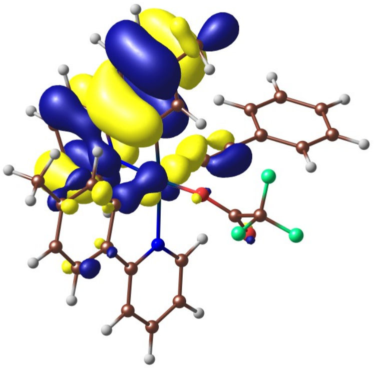

150 (HOMO–2)

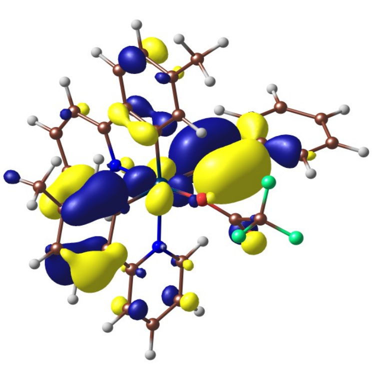

149 (HOMO–3)

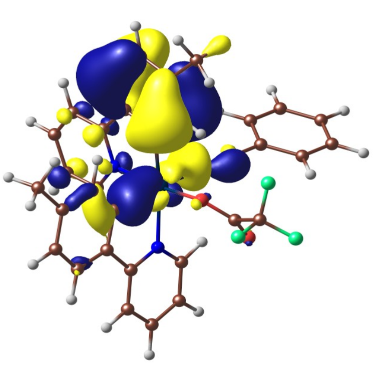

148 (HOMO–4)

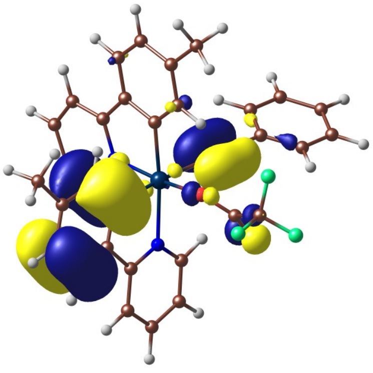

147 (HOMO–5)

Figure S47. Molecular orbital isosurfaces of *mer*-**5b** (0.03 e bohr^{-3}).

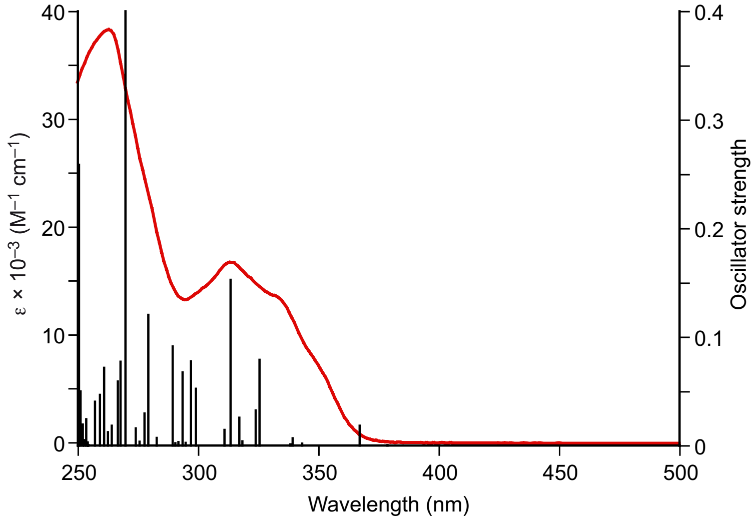

Figure S48. Calculated stick absorption spectrum of *mer*-**5b** compared with the experimental spectrum in MeCN solution (*ca.* 5×10^{-5} M) at 298 K.

Table S16. Lowest vertical singlet excitations of *mer*-**5b** from TDDFT calculations at the ground state geometry in MeCN solution.

State	Monoexcitations (Coefficient)	ΔE/eV	λ/nm	Oscillator strength	Main character
S1	152 → 153 (0.701)	3.274	378.7	0.0004	LLCT (L3 → L1)
S2	152 → 154 (0.328)<br>152 → 155 (0.601)	3.377	367.1	0.0185	LMCT (L3 → Pt)/LLCT (L3 → L2)
S3	152 → 154 (0.619)<br>152 → 155 (-0.320)	3.612	343.3	0.0017	LLCT (L3 → L2)/LMCT (L3 → Pt)
S4	149 → 155 (0.208)<br>150 → 153 (-0.115)<br>150 → 155 (-0.150)<br>151 → 153 (0.416)<br>151 → 154 (0.224)<br>151 → 155 (0.393)	3.654	339.3	0.0068	LLCT (L2 → L3)/LMCT (L2 → Pt)
S5	149 → 155 (-0.158)<br>151 → 153 (0.538)<br>151 → 154 (-0.194)<br>151 → 155 (-0.326)	3.665	338.3	0.0012	LLCT (L2 → L3)
S6	148 → 153 (0.113)<br>148 → 155 (0.106)<br>150 → 153 (0.525)<br>150 → 154 (0.135)<br>150 → 155 (0.341)<br>151 → 153 (0.134)<br>151 → 155 (0.107)	3.809	325.5	0.079	LC (L1)
S7	148 → 155 (0.106)<br>149 → 155 (0.181)<br>150 → 153 (-0.383)	3.828	323.9	0.0323	LMCT (L1 → Pt)/LC (L1)

	150 → 154 (0.229)<br>150 → 155 (0.452)				
S8	149 → 153 (-0.116)<br>152 → 156 (0.688)	3.893	318.5	0.0039	LLCT (L3 → L1)
S9	149 → 153 (0.671)<br>151 → 153 (-0.111)<br>152 → 156 (0.100)	3.911	317.1	0.0258	LLCT (L3,L2 → L1)
S10	149 → 155 (-0.198)<br>150 → 155 (0.139)<br>151 → 154 (0.589)<br>151 → 155 (-0.193)	3.956	313.4	0.1525	LC (L2)

Table S17. Lowest vertical triplet excitations of *mer*-**5b** from TDDFT calculations at the ground state geometry in MeCN solution.

**State**	**Monoexcitations (Coefficient)**	**ΔE/eV**	**λ/nm**	**Main character**
T1	148 → 153 (-0.179)<br>149 → 153 (0.132)<br>150 → 153 (0.583)<br>150 → 156 (0.183)	2.893	428.6	LC (L1)
T2	149 → 154 (-0.156)<br>150 → 154 (0.105)<br>151 → 154 (0.558)<br>151 → 155 (-0.203)<br>151 → 157 (-0.178)	2.921	424.5	LC (L2)
T3	146 → 161 (0.190)<br>152 → 154 (0.195)<br>152 → 155 (0.332)<br>152 → 158 (0.511)<br>152 → 159 (0.121)	3.099	400.1	LC (L3)/LMCT (L3 → Pt)
T4	150 → 155 (0.132)<br>152 → 153 (0.504)<br>152 → 154 (0.137)<br>152 → 155 (0.334)<br>152 → 158 (-0.235)	3.245	382.0	LLCT (L3 → L1)/LMCT (L3 → Pt)
T5	150 → 155 (-0.144)<br>151 → 155 (0.106)<br>152 → 153 (0.487)<br>152 → 154 (-0.139)<br>152 → 155 (-0.312)<br>152 → 158 (0.257)	3.291	376.7	LLCT (L3 → L1)/LMCT (L3 → Pt)

7.5. Complex *fac*-5a

Table S18. Fragment contributions (%; from atomic orbital contributions) to the frontier orbitals of *fac*-**5a** in MeCN solution.

energy (a.u.)	number	L1	L2	L3	L4	Pt
–0.010	167 (LUMO+6)	7	3	84	0	5
–0.022	166 (LUMO+5)	26	17	18	0	38
–0.032	165 (LUMO+4)	22	28	12	6	32
–0.039	164 (LUMO+3)	51	46	0	0	2
–0.046	163 (LUMO+2)	44	53	0	0	2
–0.065	162 (LUMO+1)	20	78	0	0	2
–0.066	161 (LUMO)	74	18	1	1	6
–0.202	160 (HOMO)	0	0	95	0	4
–0.229	159 (HOMO–1)	91	2	0	2	4
–0.233	158 (HOMO–2)	1	94	0	0	3
–0.243	157 (HOMO–3)	21	5	63	0	11
–0.246	156 (HOMO–4)	65	2	14	11	7
–0.252	155 (HOMO–5)	2	87	4	2	5

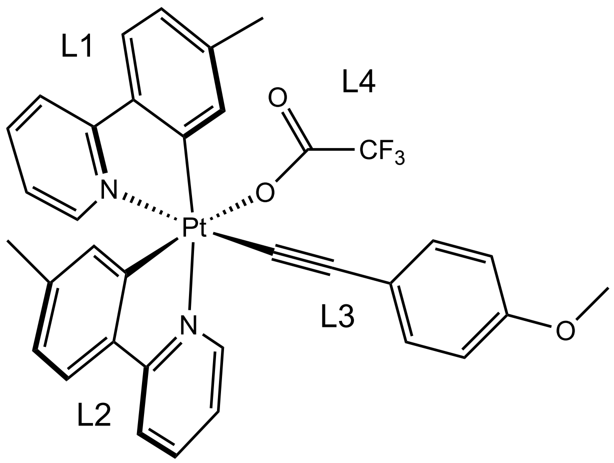

Figure S49. Ligand numbering in complex *fac*-**5a**.

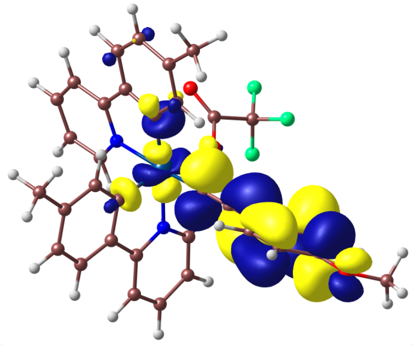

167 (LUMO+6)

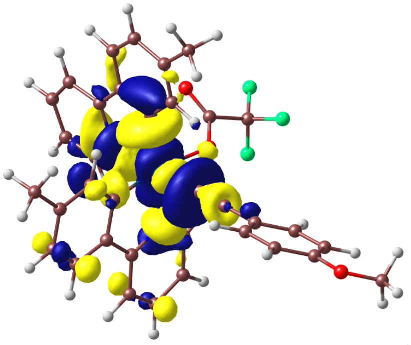

166 (LUMO+5)

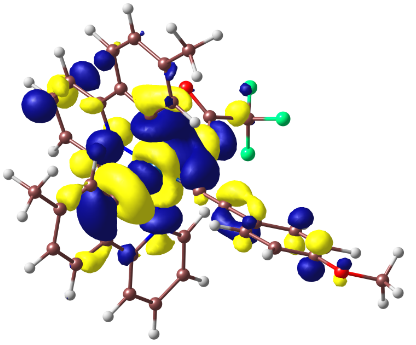

165 (LUMO+4)

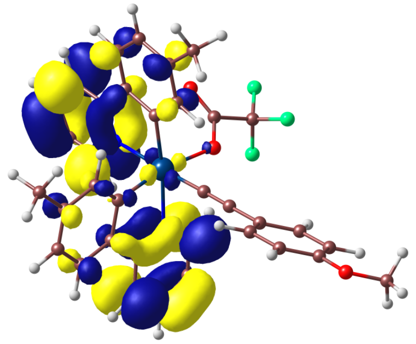

164 (LUMO+3)

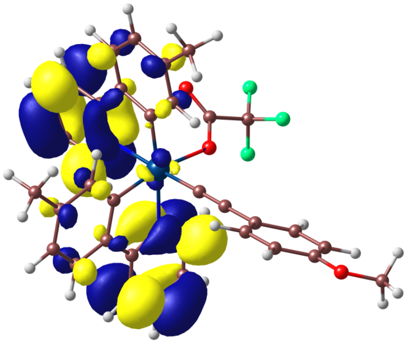

163 (LUMO+2)

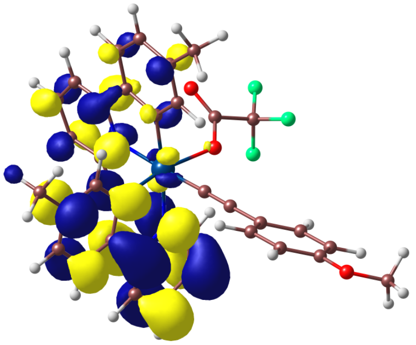

162 (LUMO+1)

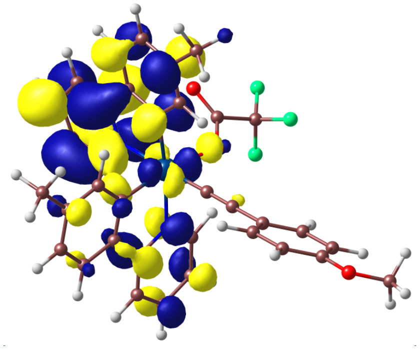

161 (LUMO)

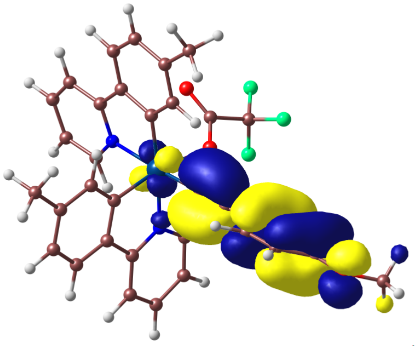

160 (HOMO)

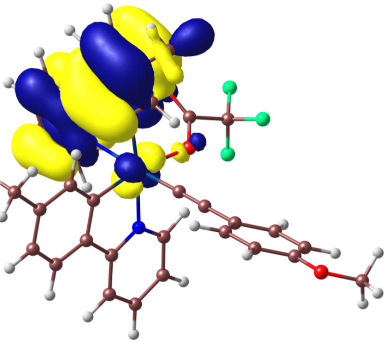

159 (HOMO–1)

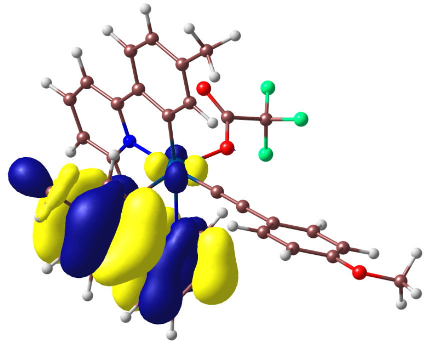

158 (HOMO–2)

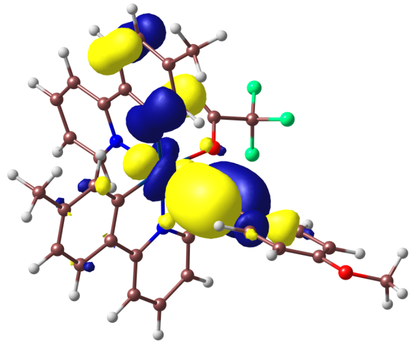

157 (HOMO–3)

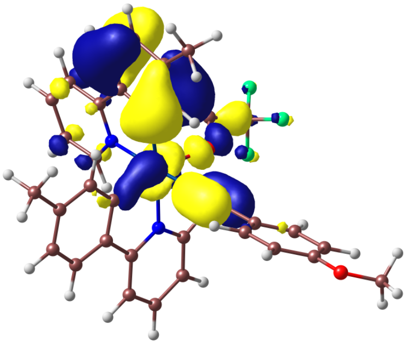

156 (HOMO–4)

Figure S50. Molecular orbital isosurfaces of *fac*-**5a** (0.03 e bohr^{-3}).

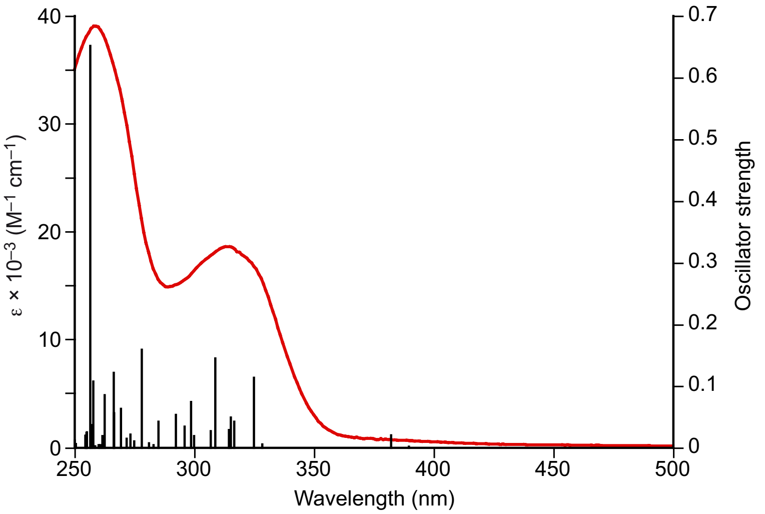

Figure S51. Calculated stick absorption spectrum of *fac*-**5a** compared with the experimental spectrum in MeCN solution (*ca.* 5×10^{-5} M) at 298 K.

Table S19. Selected vertical singlet excitations of *fac*-**5a** from TDDFT calculations at the ground state geometry in MeCN solution.

State	Monoexcitations (Coefficient)	ΔE/eV	λ/nm	Oscillator strength	Main character
S1	160 → 161 (0.498)<br>160 → 162 (0.495)	3.182	389.6	0.0017	LLCT (L3 → L1,L2)
S2	160 → 161 (-0.492)<br>160 → 162 (0.499)	3.244	382.2	0.0207	LLCT (L3 → L2)
S3	159 → 161 (0.102)<br>160 → 163 (0.674)<br>160 → 164 (0.152)	3.776	328.4	0.0056	LLCT (L3 → L1,L2)
S4	156 → 161 (0.121)<br>159 → 161 (0.654)<br>159 → 162 (-0.108)	3.817	324.8	0.1139	LC (L1)
S5	158 → 162 (-0.131)<br>159 → 161 (0.123)<br>159 → 162 (0.659)	3.916	316.6	0.0426	LLCT (L1 → L2)
S6	158 → 161 (0.104)<br>160 → 164 (0.506)<br>160 → 165 (0.438)	3.934	315.2	0.0492	LLCT (L3 → L1,L2)/LMCT (L3 → Pt)
S7	157 → 161 (-0.109)<br>158 → 161 (0.663)<br>160 → 164 (-0.106)	3.944	314.4	0.0289	LLCT (L2 → L1)
S8	157 → 162 (-0.128)<br>158 → 162 (0.633)<br>159 → 162 (0.155)<br>160 → 166 (-0.101)	4.017	308.7	0.1452	LC (L2)
S9	158 → 162 (0.109)<br>160 → 163 (0.162)	4.042	306.7	0.0274	LLCT (L3 → L1)/LMCT (L3 → Pt)

	160 → 164 (-0.428)<br>160 → 165 (0.510)				
S10	152 → 166 (0.105)<br>157 → 161 (-0.161)<br>157 → 162 (-0.184)<br>160 → 166 (0.628)	4.135	299.8	0.0188	LMCT (L3 → Pt)

Table S20. Selected vertical triplet excitations of *fac*-**5a** from TDDFT calculations at the ground state geometry in MeCN solution.

**State**	**Monoexcitations (Coefficient)**	**ΔE/eV**	**λ/nm**	**Main character**
T1	159 → 161 (0.548)<br>159 → 162 (-0.282)<br>159 → 163 (-0.139)<br>159 → 164 (0.146)	2.880	430.6	LC (L1)
T2	155 → 169 (0.119)<br>158 → 161 (0.277)<br>158 → 162 (0.558)<br>158 → 163 (0.120)<br>158 → 164 (0.149)	2.929	423.3	LC (L2)
T3	154 → 168 (-0.151)<br>160 → 161 (0.377)<br>160 → 162 (-0.143)<br>160 → 165 (-0.310)<br>160 → 167 (0.426)	3.067	404.3	LC (L3)
T4	160 → 161 (0.392)<br>160 → 162 (0.566)	3.168	391.3	LLCT (L3 → L2)
T5	154 → 168 (0.117)<br>160 → 161 (0.426)<br>160 → 162 (-0.389)<br>160 → 165 (0.124)<br>160 → 167 (-0.331)	3.286	377.4	LLCT (L3 → L1)
T11	155 → 161 (0.131)<br>155 → 162 (0.283)<br>159 → 162 (-0.189)<br>160 → 164 (-0.236)<br>160 → 165 (-0.283)<br>160 → 166 (0.343)<br>160 → 167 (-0.107)	3.840	322.9	LMCT (L3 → Pt)/LC (L2)
T15	152 → 166 (0.111)<br>160 → 164 (0.327)<br>160 → 165 (0.263)<br>160 → 166 (0.450)<br>160 → 167 (0.200)<br>160 → 168 (0.137)	3.980	311.6	LMCT (L3 → Pt)

7.6. Complex *fac*-5b

Table S21. Fragment contributions (%; from atomic orbital contributions) to the frontier orbitals of *fac*-**5b** in MeCN solution.

energy (a.u.)	number	L1	L2	L3	L4	Pt
–0.019	159 (LUMO+6)	15	7	64	0	14
–0.024	158 (LUMO+5)	19	17	31	1	33
–0.033	157 (LUMO+4)	20	26	20	5	29
–0.040	156 (LUMO+3)	51	46	0	0	2
–0.046	155 (LUMO+2)	44	53	0	0	2
–0.066	154 (LUMO+1)	19	78	0	0	2
–0.067	153 (LUMO)	74	18	0	1	6
–0.217	152 (HOMO)	1	1	92	0	6
–0.230	151 (HOMO–1)	92	1	0	2	4
–0.234	150 (HOMO–2)	1	95	0	0	3
–0.245	149 (HOMO–3)	39	5	44	1	10
–0.247	148 (HOMO–4)	47	4	29	11	9
–0.253	147 (HOMO–5)	2	85	7	3	3

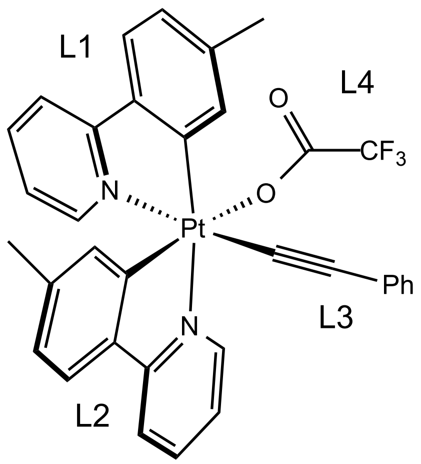

Figure S52. Ligand numbering in complex *fac*-**5b**.

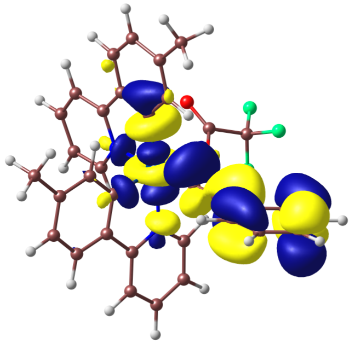

159 (LUMO+6)

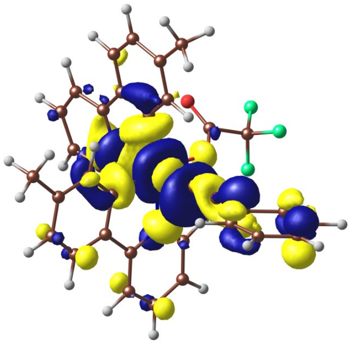

158 (LUMO+5)

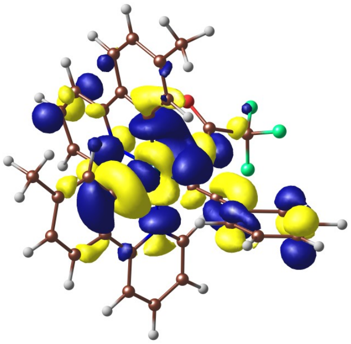

157 (LUMO+4)

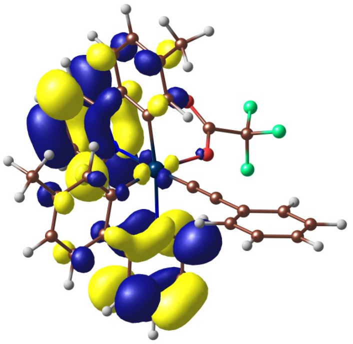

156 (LUMO+3)

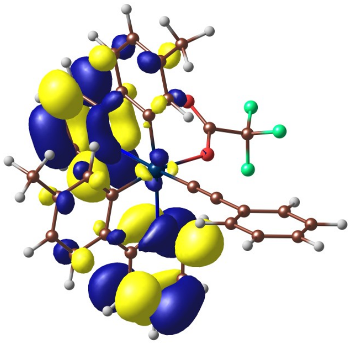

155 (LUMO+2)

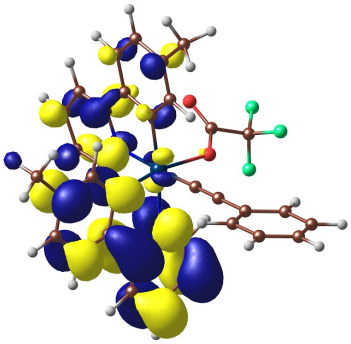

154 (LUMO+1)

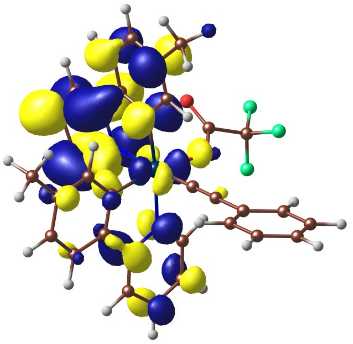

153 (LUMO)

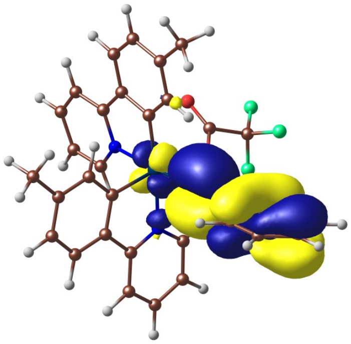

152 (HOMO)

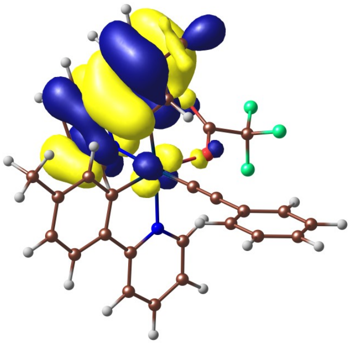

151 (HOMO–1)

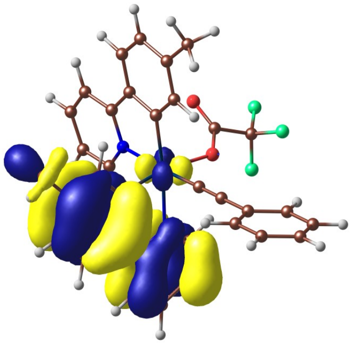

150 (HOMO–2)

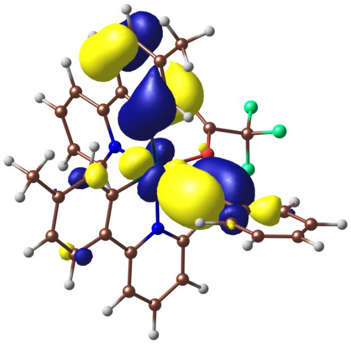

149 (HOMO–3)

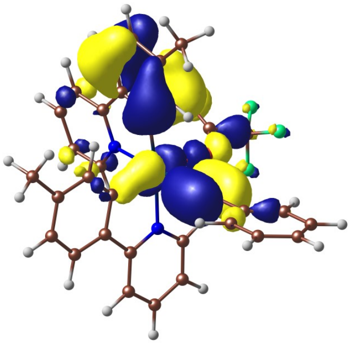

148 (HOMO–4)

Figure S53. Molecular orbital isosurfaces of *fac*-**5b** (0.03 e bohr^{-3}).

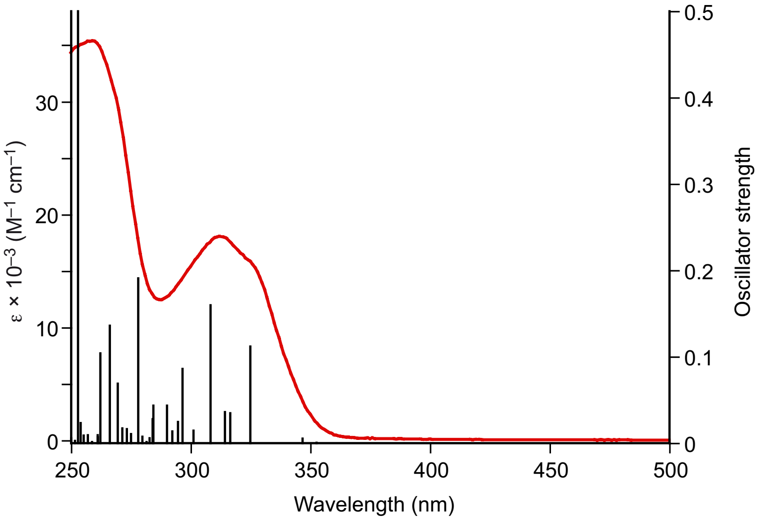

Figure S54. Calculated stick absorption spectrum of *fac*-**5b** compared with the experimental spectrum in MeCN solution (*ca.* 5×10^{-5} M) at 298 K.

Table S22. Selected vertical singlet excitations of *fac*-**5b** from TDDFT calculations at the ground state geometry in MeCN solution.

State	Monoexcitations (Coefficient)	ΔE/eV	λ/nm	Oscillator strength	Main character
S1	152 → 153 (0.512)<br>152 → 154 (0.481)	3.516	352.6	0.0005	LLCT (L3 → L1,L2)
S2	152 → 153 (-0.475)<br>152 → 154 (0.513)	3.576	346.7	0.0053	LLCT (L3 → L2)
S3	148 → 153 (0.117)<br>151 → 153 (0.665)<br>151 → 154 (-0.103)	3.817	324.9	0.1121	LC (L1)
S4	150 → 154 (-0.129)<br>151 → 153 (0.125)<br>151 → 154 (0.667)	3.917	316.5	0.0346	LLCT (L1 → L2)
S5	150 → 153 (0.678)	3.945	314.3	0.0362	LLCT (L2 → L1)
S6	149 → 154 (-0.114)<br>150 → 154 (0.653)<br>151 → 154 (0.150)	4.021	308.3	0.1598	LC (L2)
S7	152 → 155 (0.673)<br>152 → 156 (0.149)<br>152 → 157 (-0.122)	4.117	301.2	0.0145	LLCT (L3 → L1,L2)
S8	148 → 154 (0.163)<br>149 → 153 (0.633)<br>150 → 153 (0.110)	4.181	296.6	0.0861	LLCT (L3 → L1)/LC (L1)
S9	148 → 153 (0.430)<br>148 → 154 (0.108)<br>149 → 154 (0.488)<br>151 → 155 (-0.140)	4.207	294.7	0.0246	LLCT (L3 → L1,L2)
S10	148 → 153 (0.161)	4.243	292.2	0.0136	LLCT (L3 → L1,L2)/LMCT (L3 → Pt)

	148 → 154 (-0.116)<br>149 → 154 (-0.108)<br>152 → 156 (0.403)<br>152 → 157 (0.484)<br>152 → 158 (0.148)				

Table S23. Selected vertical triplet excitations of *fac*-**5b** from TDDFT calculations at the ground state geometry in MeCN solution.

**State**	**Monoexcitations (Coefficient)**	**ΔE/eV**	**λ/nm**	**Main character**
T1	151 → 153 (0.555)<br>151 → 154 (-0.275)<br>151 → 155 (-0.140)<br>151 → 156 (0.146)	2.880	430.5	LC (L1)
T2	147 → 160 (0.121)<br>150 → 153 (0.268)<br>150 → 154 (0.563)<br>150 → 155 (0.120)<br>150 → 156 (0.150)	2.929	423.4	LC (L2)
T3	146 → 161 (0.230)<br>152 → 153 (0.106)<br>152 → 157 (-0.369)<br>152 → 158 (0.261)<br>152 → 159 (0.448)	3.165	391.7	LC (L3)
T4	152 → 153 (0.542)<br>152 → 154 (0.428)<br>152 → 158 (-0.110)	3.486	355.7	LLCT (L3 → L2)/LMCT (L3 → Pt)
T5	152 → 153 (-0.402)<br>152 → 154 (0.547)<br>152 → 158 (0.106)<br>152 → 159 (0.116)	3.561	348.2	LLCT (L3 → L1)/LMCT (L3 → Pt)
T15	152 → 153 (0.146)<br>152 → 155 (-0.286)<br>152 → 156 (0.157)<br>152 → 157 (0.322)<br>152 → 158 (0.457)	4.099	302.5	LMCT (L3 → Pt)

Table S24. Energies, free energies, enthalpies and entropies of the optimized structures in MeCN solution.[a]

Complex	E_0 [b]	ZPE [c]	G [d]	H [e]	S [f]
mer-**4a**	-1806.169525	-1805.608262	-1805.686352	-1805.568622	247.783
mer-**4b**	-1691.644529	-1691.115831	-1691.190351	-1691.078809	234.761
mer-**4d**	-1890.106360	-1889.594287	-1889.671134	-1889.555571	243.222
mer-**5b**	-1989.351617	-1988.845544	-1988.922650	-1988.806662	244.116
fac-**5a**	-2103.900205	-2103.361467	-2103.442128	-2103.319896	257.258
fac-**5b**	-1989.374924	-1988.868700	-1988.945459	-1988.829779	243.468

[a] Thermal corrections from vibrational calculations at 298.15 K. [b] Electronic energy (Hartrees). [c] Sum of electronic and zero-point energies (Hartrees). [d] Free Energy (Hartrees). [e] Enthalpy (Hartrees). [f] Entropy (cal mol^{-1} K^{-1}).

Table S25. Cartesian coordinates (Å) of the optimized structures.

mer-**4a**

Pt	-0.434058572	-0.015561415	0.153554709
C	-0.117857922	1.176856324	1.752733603
C	-0.221298502	0.804906547	3.089125368
C	0.007075339	1.735484864	4.114958659
C	0.338863484	3.057021698	3.768291241
C	0.446062306	3.440532241	2.437152274
C	0.219412265	2.504129709	1.414016845
C	0.298762784	2.802730407	-0.012307958
C	0.628215803	4.041216592	-0.580325067
C	0.672173584	4.184205990	-1.960431228
C	0.387728972	3.084309501	-2.774667196
C	0.066804809	1.878285308	-2.171174818
H	-0.487448621	-0.217831511	3.335391791
H	0.512387693	3.788960274	4.552265583
H	0.700256613	4.469087614	2.199694567
H	0.849364146	4.882392011	0.065025905
H	0.927152339	5.142788165	-2.400077370
H	0.412673000	3.152282678	-3.855562041
H	-0.160152163	0.992315082	-2.748387651
N	0.022845574	1.747855885	-0.833484997
C	-2.465212130	0.402709096	-0.015323359
C	-3.233145629	1.201204076	0.833755608
C	-4.608191427	1.403193090	0.632779841
C	-5.221428821	0.771369944	-0.459096977
C	-4.481316615	-0.028906214	-1.322024799
C	-3.104241142	-0.221782957	-1.114383277
C	-2.282438240	-1.063424535	-2.000824984
C	-2.752593107	-1.755603849	-3.128044534
C	-1.870623424	-2.510336847	-3.892483098
C	-0.523375182	-2.577726644	-3.527757723
C	-0.114221887	-1.883812225	-2.396822437
H	-2.762685279	1.682429918	1.686948393
H	-6.285805705	0.906741247	-0.632619230
H	-4.988242077	-0.505777054	-2.155276593
H	-3.798270735	-1.700470354	-3.404163725
H	-2.231231875	-3.043204642	-4.766768275
H	0.192738851	-3.156785680	-4.099163052
H	0.908469524	-1.899479619	-2.040014152
N	-0.967839566	-1.147996441	-1.668613391
C	-0.077561836	1.320257178	5.563762346
H	-0.708371064	0.436189298	5.690266031
H	0.916501673	1.073118993	5.957162027
H	-0.479672216	2.124874995	6.186876071
C	-5.403491068	2.296372409	1.554547261
H	-5.486024250	3.310790690	1.143977001
H	-6.422543942	1.922257201	1.693531628
H	-4.930332999	2.380175304	2.537048974
C	1.580384065	-0.525090352	0.161885672
C	2.778368796	-0.782625274	0.144031880
C	4.170727332	-1.111363718	0.119134556
C	4.619116339	-2.412446105	0.414455761
C	5.148792020	-0.143271028	-0.203457806
C	5.974208466	-2.745350321	0.393034052
H	3.889751368	-3.176724687	0.665187486
C	6.498676302	-0.463742694	-0.228302384
H	4.834448277	0.869799653	-0.435604089
C	6.924907409	-1.768301722	0.069831107
H	6.273522072	-3.759930319	0.627561846
H	7.245561199	0.283896418	-0.476930269
O	-0.985832846	-1.571382649	1.406493363
C	-0.665279563	-2.832880324	1.219983095
C	-1.153937985	-3.697806165	2.380103394
O	-0.052569398	-3.308941639	0.270880845
H	-0.593893960	-3.447238991	3.286839107
H	-1.005190923	-4.752779114	2.146066136
H	-2.210812285	-3.507793193	2.585260297
O	8.272253752	-1.981208991	0.018016543
C	8.759396759	-3.287556687	0.312555044
H	9.844129314	-3.233580629	0.213491421
H	8.503784251	-3.590820522	1.334733181
H	8.368046159	-4.030874527	-0.392070736

mer-**4b**

Pt	-0.433217111	-0.014776004	0.151427640
C	-0.117157593	1.177426655	1.751477299
C	-0.219238061	0.804259559	3.087516082
C	0.009322406	1.734374745	4.113813128
C	0.339814207	3.056323951	3.767652164
C	0.445718578	3.440854483	2.436671162

C	0.218962251	2.505009800	1.413082900
C	0.297127531	2.804545242	-0.013163900
C	0.624929507	4.043709287	-0.580585549
C	0.667831531	4.187611071	-1.960646386
C	0.384016532	3.087984785	-2.775448817
C	0.064719981	1.881230523	-2.172560908
H	-0.484449617	-0.218838704	3.333301986
H	0.513438007	3.787891336	4.551939356
H	0.699011221	4.469747980	2.199765096
H	0.845573374	4.884729863	0.065128658
H	0.921493270	5.146753227	-2.399821915
H	0.408167987	3.156711071	-3.856308732
H	-0.161713943	0.995417231	-2.750237675
N	0.021821206	1.749956708	-0.834909712
C	-2.463735212	0.402817324	-0.016030704
C	-3.230928525	1.200883844	0.833953917
C	-4.606130149	1.402775416	0.633792961
C	-5.219878522	0.771400732	-0.458020562
C	-4.480244877	-0.028468020	-1.321730841
C	-3.103077153	-0.221395623	-1.114964330
C	-2.281656672	-1.062824088	-2.001805644
C	-2.752429962	-1.755186207	-3.128603377
C	-1.870953261	-2.510078291	-3.893409470
C	-0.523535851	-2.577502511	-3.529367316
C	-0.113686747	-1.883423603	-2.398832655
H	-2.760020321	1.681745311	1.687047402
H	-6.284346181	0.906779523	-0.630895552
H	-4.987583230	-0.505011908	-2.154893595
H	-3.798283711	-1.700135940	-3.404033600
H	-2.232061767	-3.043111951	-4.767371944
H	0.192269434	-3.156746574	-4.100942664
H	0.909283759	-1.899947681	-2.043113972
N	-0.966807437	-1.147203083	-1.670247538
C	-0.073729499	1.318034406	5.562362990
H	-0.706388004	0.435330543	5.689171163
H	0.920459707	1.068095222	5.953665276
H	-0.472784448	2.123030214	6.186915125
C	-5.400898893	2.295499483	1.556421850
H	-5.481376222	3.310775450	1.147577455
H	-6.420643772	1.922709780	1.693732096
H	-4.928527070	2.376809033	2.539509727
C	1.581732012	-0.522328459	0.161625899
C	2.779624830	-0.779555004	0.144578538
C	4.171316121	-1.111147263	0.121713836
C	4.607786204	-2.417328077	0.431227493
C	5.144658585	-0.144932455	-0.210700426
C	5.963197769	-2.740748951	0.408539792
H	3.869840820	-3.171253492	0.688130342
C	6.498821826	-0.474643721	-0.232241610
H	4.825170467	0.864742084	-0.450799341
C	6.915692158	-1.772632726	0.076821191
H	6.277313416	-3.752373144	0.650261534
H	7.231567583	0.284670798	-0.491090433
O	-0.984398321	-1.571588621	1.402953061
C	-0.658056675	-2.831879046	1.216936404
C	-1.147654228	-3.699332545	2.374610947
O	-0.039727273	-3.304473700	0.269809652
H	-0.591776510	-3.447291258	3.283492071
H	-0.994091139	-4.753554671	2.140357774
H	-2.205947383	-3.513356053	2.576061646
H	7.971402251	-2.026971012	0.059573021

mer-**4d**

Pt	-0.433743119	-0.015237711	0.148176618
C	-0.116624588	1.178315006	1.748085251
C	-0.216538930	0.804717689	3.083952772
C	0.013417721	1.735048446	4.109903058
C	0.342997878	3.056942861	3.762966749
C	0.446832871	3.441555828	2.431782622
C	0.218730410	2.505689364	1.408581311
C	0.294710165	2.805013971	-0.017930011
C	0.620772370	4.044236342	-0.586065936
C	0.661518759	4.187800183	-1.966264534
C	0.377268636	3.087892306	-2.780519203
C	0.059718651	1.881001606	-2.177004288
H	-0.481181066	-0.218425645	3.330209516
H	0.517672236	3.788624871	4.546888545
H	0.699584457	4.470484055	2.194547630
H	0.841701135	4.885607827	0.059076353
H	0.913831302	5.147035718	-2.405987094
H	0.399717099	3.156516064	-3.861410752
H	-0.167055885	0.994908236	-2.754172664
N	0.019087978	1.750147397	-0.839217177
C	-2.462528313	0.402904512	-0.016799266
C	-3.226881271	1.203399220	0.833109451
C	-4.602086283	1.406917694	0.634162898
C	-5.217935108	0.774895492	-0.455984470
C	-4.480621503	-0.027501704	-1.319355576
C	-3.103619136	-0.222427872	-1.113925581
C	-2.284517817	-1.066972366	-1.999680029
C	-2.758010146	-1.762604182	-3.123213252
C	-1.878776331	-2.521141757	-3.886949502
C	-0.530879078	-2.589053259	-3.524881565
C	-0.118176653	-1.891589554	-2.397534800
H	-2.754224777	1.684838537	1.684790049
H	-6.282340494	0.911736337	-0.627916104
H	-4.989572074	-0.504340502	-2.151315087
H	-3.804346320	-1.707560075	-3.396743718
H	-2.242010379	-3.056797522	-4.758398114
H	0.183264381	-3.171259233	-4.095481793
H	0.905453166	-1.909476819	-2.044211030
N	-0.969082661	-1.151458372	-1.670033417
C	-0.067193519	1.318630672	5.558521851
H	-0.704214790	0.439314271	5.687046569
H	0.926913331	1.062978962	5.946320825
H	-0.459751714	2.125548976	6.184639239
C	-5.394126589	2.302402103	1.556389830
H	-5.465105239	3.319585468	1.150549933
H	-6.417116859	1.936711408	1.688115615
H	-4.925271191	2.377061149	2.541694981
C	1.582278149	-0.519943780	0.160268962
C	2.780364541	-0.774874410	0.145299482
C	4.168677839	-1.106776005	0.125775032
C	4.590341924	-2.424928052	0.401637172

C	5.139045845	-0.126052135	-0.169164785
C	5.945225481	-2.719950808	0.375509525
H	3.870625971	-3.201497998	0.630971579
C	6.479146064	-0.484077464	-0.180203057
H	4.850830351	0.895756057	-0.385070492
C	6.924200587	-1.774322489	0.087841128
O	-0.985923182	-1.572547135	1.397942043
C	-0.644406849	-2.829894404	1.216725924
C	-1.140969015	-3.701970086	2.367607869
O	-0.008345769	-3.295418102	0.277944922
H	-0.602077564	-3.442525583	3.284569926
H	-0.971392504	-4.754439635	2.136676203
H	-2.204336457	-3.528309967	2.552631503
H	7.976533524	-2.029123853	0.073519321
F	7.400677445	0.463260081	-0.464454046
F	6.338681096	-3.985570916	0.641144567

mer-**5b**

Pt	-0.439551231	-0.053979129	0.071029812
C	-0.043858316	1.058538085	1.710307558
C	-0.107387166	0.625350557	3.030333956
C	0.175746272	1.507626755	4.085223433
C	0.520786699	2.837035106	3.783570138
C	0.592551559	3.277101526	2.467726865
C	0.312085655	2.390022584	1.414512202
C	0.358210585	2.738113981	-0.000983432
C	0.700020568	3.986728804	-0.537575144
C	0.712978161	4.173829088	-1.912942264
C	0.385037363	3.107790508	-2.755566400
C	0.051722819	1.889500204	-2.185442400
H	-0.376545508	-0.403869349	3.244929057
H	0.735431247	3.530320803	4.592004519
H	0.862029643	4.309327627	2.266042525
H	0.955427078	4.800964740	0.129178549
H	0.977868602	5.140765968	-2.327519787
H	0.385338958	3.210917502	-3.833861324
H	-0.209482697	1.027965732	-2.784469622
N	0.038100864	1.717795924	-0.851078431
C	-2.459027805	0.434492928	-0.035388865
C	-3.172994289	1.245556744	0.846645408
C	-4.545604348	1.496864433	0.686369335
C	-5.208838599	0.902899532	-0.397112221
C	-4.520863571	0.091239239	-1.292167894
C	-3.146365169	-0.151728065	-1.125969475
C	-2.378288994	-1.006386530	-2.047140323
C	-2.904232996	-1.672252617	-3.164913033
C	-2.070492692	-2.446113901	-3.963773830
C	-0.715205230	-2.558741780	-3.643219372
C	-0.248422458	-1.887502147	-2.521128105
H	-2.662828442	1.697420779	1.692877930
H	-6.272119118	1.077027068	-0.539247180
H	-5.066182408	-0.354315289	-2.118304524
H	-3.956126642	-1.583728079	-3.406333976
H	-2.475224651	-2.959376610	-4.830348021
H	-0.036004956	-3.154427545	-4.241783136
H	0.785115842	-1.937544666	-2.199912514
N	-1.055891728	-1.133035278	-1.758939322
C	0.135426681	1.032024691	5.517043404
H	-0.496997938	0.147089635	5.627110049
H	1.140343760	0.763534930	5.866213550
H	-0.240298534	1.812231901	6.185939639
C	-5.283509207	2.402982801	1.642231074
H	-5.306492508	3.433870719	1.266549063
H	-6.322110598	2.084816658	1.773964247
H	-4.802387126	2.425484376	2.624215198
C	1.555879682	-0.633520863	0.017401092
C	2.741054132	-0.938456718	-0.034162467
C	4.116937761	-1.326241786	-0.096815909
C	4.506098827	-2.651974710	0.190717648
C	5.119327971	-0.397199816	-0.447248579
C	5.845935372	-3.030415936	0.129339317
H	3.744437454	-3.376947774	0.461166213
C	6.457641782	-0.782153851	-0.507470325
H	4.835282060	0.626775219	-0.670561657
C	6.828232759	-2.098975086	-0.219947886
H	6.124432627	-4.056117462	0.354580374
H	7.214147491	-0.051381146	-0.779565328
H	7.871723514	-2.396223652	-0.267286670
O	-1.012607834	-1.650319405	1.276246913
C	-0.692738491	-2.888167143	1.082571951
C	-1.194113720	-3.730407468	2.289898010
O	-0.111165830	-3.437020398	0.163391320
F	-0.680249564	-3.266262330	3.451195639
F	-0.840370101	-5.018849329	2.172150711
F	-2.539606789	-3.678448845	2.391144942

fac-**5a**

Pt	-0.610508760	0.178291252	0.099827169
C	-0.204328591	1.302719418	1.739557888
C	-0.149197585	0.858209624	3.058189640
C	0.144302162	1.737108962	4.115008026
C	0.372932577	3.088402549	3.819586032
C	0.322978737	3.548730382	2.507557635
C	0.037256312	2.667178104	1.453852557
C	-0.013740457	3.079050193	0.048429124
C	0.196288243	4.379222575	-0.429490118
C	0.139275314	4.632985852	-1.794554735
C	-0.130527095	3.585696673	-2.678595066
C	-0.340140186	2.318540522	-2.152339945
H	-0.341714841	-0.186393817	3.277836600
H	0.592721049	3.785303263	4.623649234
H	0.509201876	4.600098449	2.311878948
H	0.405781412	5.183321576	0.264842071
H	0.303716265	5.638925519	-2.166973670
H	-0.181493816	3.741313472	-3.749577946
H	-0.557235878	1.468807085	-2.787755676
N	-0.283998276	2.078701819	-0.832685479
C	-2.608940550	0.516937052	0.088894194
C	-3.324167423	1.238872939	1.038939535
C	-4.710194914	1.436443139	0.915930336
C	-5.369830209	0.878146837	-0.187144734
C	-4.669023881	0.147525832	-1.140649170
C	-3.283698101	-0.048315405	-1.021021207
C	-2.499784476	-0.820287830	-1.997553393

C	-3.011933928	-1.475824271	-3.125856466
C	-2.149384479	-2.173498478	-3.964926844
C	-0.782835257	-2.215470351	-3.675989076
C	-0.328200477	-1.549698622	-2.544378666
H	-2.811249537	1.658175756	1.897402524
H	-6.441888387	1.013947303	-0.298390391
H	-5.210696635	-0.274666451	-1.980968570
H	-4.072310010	-1.443003317	-3.343267325
H	-2.541273888	-2.683276417	-4.839214058
H	-0.084417169	-2.750640078	-4.308516086
H	0.715983575	-1.533830591	-2.250455175
N	-1.167613561	-0.875916620	-1.743586107
C	-0.919562180	-1.501424655	1.103859196
C	-1.119460140	-2.545975697	1.703575153
C	-1.341703192	-3.769087854	2.411708903
C	-0.499647283	-4.881325862	2.231075550
C	-2.417844819	-3.900391975	3.318050563
C	-0.710209140	-6.077313573	2.918150914
H	0.334903610	-4.806926716	1.540637006
C	-2.635904237	-5.085569219	4.004936412
H	-3.083672805	-3.057724196	3.476798403
C	-1.784108289	-6.185889738	3.811813678
H	-0.036997228	-6.909274773	2.749881259
H	-3.463683195	-5.183681824	4.700239852
O	1.480768856	-0.498065570	-0.210063474
C	2.526224987	0.224403407	-0.376705734
C	3.712157086	-0.654378893	-0.871996522
O	2.711516681	1.425609822	-0.219791909
C	0.232139358	1.226858002	5.533007623
H	-0.464761100	0.401234151	5.704615624
H	1.239660069	0.850687020	5.750752101
H	0.016335144	2.017873916	6.256828118
C	-5.460368150	2.251910894	1.940702770
H	-5.410480547	3.321390888	1.700675754
H	-6.517398699	1.974048927	1.975689472
H	-5.035884862	2.125071536	2.940931576
F	4.001260609	-1.636588314	0.009617014
F	3.405537929	-1.250987442	-2.051420218
F	4.832719810	0.061410401	-1.064603314
O	-2.084046716	-7.303172356	4.533460963
C	-1.252080087	-8.450532456	4.379260533
H	-1.668079001	-9.213639970	5.037869457
H	-1.262769991	-8.818284291	3.346562871
H	-0.218312067	-8.239463020	4.676882074

fac-**5b**

Pt	-0.582240175	0.174578656	0.133450347
C	-0.176213097	1.341666260	1.743569734
C	-0.095141507	0.926708486	3.070413803
C	0.193649135	1.834067103	4.104237719
C	0.390215161	3.183109978	3.776972351
C	0.314347708	3.613876007	2.456221964
C	0.033963480	2.703997755	1.425404522
C	-0.041845490	3.083506114	0.012114526
C	0.138100123	4.376173080	-0.497202463
C	0.060624575	4.597942386	-1.866845967
C	-0.199073540	3.526089423	-2.724071728
C	-0.378465079	2.267431467	-2.167252858
H	-0.263120206	-0.116522112	3.315440486
H	0.605394823	3.901644982	4.563022931
H	0.475984332	4.664413091	2.235771305
H	0.340656646	5.199340634	0.176516744
H	0.201639774	5.598062981	-2.263673933
H	-0.264861169	3.656435892	-3.797605745
H	-0.585608515	1.399351703	-2.780697986
N	-0.303214746	2.058713006	-0.843267812
C	-2.587052414	0.477174834	0.141902216
C	-3.302783969	1.205827672	1.086344551
C	-4.693751419	1.375398664	0.977998732
C	-5.357286380	0.782178003	-0.104317927
C	-4.655689758	0.044683559	-1.051881988
C	-3.265525345	-0.123386441	-0.946938647
C	-2.480519466	-0.901764473	-1.917422966
C	-2.995075479	-1.590321284	-3.024732302
C	-2.130977520	-2.291351866	-3.859412441
C	-0.760460780	-2.303937738	-3.586942658
C	-0.303404899	-1.605901341	-2.475986856
H	-2.786556766	1.652340000	1.928937964
H	-6.433042766	0.895950143	-0.203850040
H	-5.200445943	-0.404953563	-1.875793227
H	-4.058467056	-1.580425796	-3.229256954
H	-2.524732151	-2.826678483	-4.717425200
H	-0.060765272	-2.841063573	-4.216371325
H	0.743894823	-1.566007129	-2.195798875
N	-1.144434549	-0.928816601	-1.679586776
C	-0.845103629	-1.488410863	1.176412910
C	-1.012678839	-2.523545635	1.801297395
C	-1.192771383	-3.738255637	2.536329896
C	-0.163101666	-4.699787008	2.605583619
C	-2.404622357	-4.001748368	3.208328280
C	-0.342497677	-5.881356063	3.322571705
H	0.774370776	-4.508512182	2.092573111
C	-2.577068802	-5.185887589	3.922871498
H	-3.204952198	-3.269538715	3.162479860
C	-1.548504814	-6.130483927	3.984019977
H	0.461977311	-6.610100869	3.364947648
H	-3.517498918	-5.371607980	4.433921151
H	-1.685525319	-7.052180676	4.541743365
O	1.516186853	-0.470858124	-0.187313000
C	2.545621984	0.267198126	-0.384160273
C	3.741666904	-0.600567684	-0.874412687
O	2.709837379	1.474826730	-0.256550911
C	0.310297812	1.356712383	5.531549687
H	-0.359623527	0.514373463	5.727384957
H	1.330859572	1.015960473	5.746838942
H	0.077577287	2.156068353	6.240848499
C	-5.445527797	2.198162170	1.995710851
H	-5.421779434	3.262701231	1.731000891
H	-6.495849967	1.899342308	2.053089418
H	-5.003620899	2.102831968	2.991860547
F	4.064241983	-1.551868804	0.029195860
F	3.429671904	-1.235546552	-2.032065074
F	4.844005076	0.132314420	-1.103930016

8. References

(1) Juliá, F.; González-Herrero, P. Aromatic C–H Activation in the Triplet Excited State of Cyclometalated Platinum(II) Complexes Using Visible Light. *J. Am. Chem. Soc.* **2016**, *138*, 5276–5282.

(2) Li, Z.; Sun, K.; Cai, C. Nickel-Catalyzed Regioselective C–H Oxygenation: New Routes for Versatile C–O Bond Formation. *Org. Chem. Front.* **2019**, *6*, 637–642.

(3) Sheldrick, G. M. A Short History of SHELX. *Acta Crystallogr., Sect. A Found. Crystallogr.* **2008**, *64*, 112–122.

(4) Sheldrick, G. M. SHELXT – Integrated Space-Group and Crystal-Structure Determination. *Acta Crystallogr. Sect. A Found. Adv.* **2015**, *71*, 3–8.

(5) Frisch, M. J.; Trucks, G. W.; Schlegel, H. B.; Scuseria, G. E.; Robb, M. A.; Cheeseman, J. R.; Scalmani, G.; Barone, V.; Petersson, G. A.; Nakatsuji, H.; et al. Gaussian 16 (Revision A.03); Gaussian Inc.: Wallingford CT, 2016.

(6) Becke, A. Density Functional Thermochemistry III The Role of Exact Exchange. *J. Chem. Phys.* **1993**, *98*, 5648–5652.

(7) Lee, C. T.; Yang, W. T.; Parr, R. G. Development of The Colle-Salvetti Correlation-Energy Formula into a Functional of the Electron-Density. *Phys. Rev. B* **1988**, *37*, 785–789.

(8) Hariharan, P. C.; Pople, J. A. Influence of Polarization Functions on Molecular-Orbital Hydrogenation Energies. *Theor. Chim. Acta* **1973**, *28*, 213–222.

(9) Francl, M. M.; Pietro, W. J.; Hehre, W. J.; Binkley, J. S.; Gordon, M. S.; Defrees, D. J.; Pople, J. A. Self-Consistent Molecular-Orbital Methods. 23. A Polarization-Type Basis Set for 2nd-Row Elements. *J. Chem. Phys.* **1982**, *77*, 3654–3665.

(10) Hay, P. J.; Wadt, W. R. Ab Initio Effective Core Potentials for Molecular Calculations–Potentials for K to Au Including the Outermost Core Orbitals. *J. Chem. Phys.* **1985**, *82*, 299–310.

(11) Tomasi, J.; Mennucci, B.; Cammi, R. Quantum Mechanical Continuum Solvation Models. *Chem. Rev.* **2005**, *105*, 2999–3093.
